# Novel Ruthenacarborane–NSAID Conjugates

**DOI:** 10.3390/molecules30214153

**Published:** 2025-10-22

**Authors:** Sonam Sonam, Marija Mojić, Vuk Gordić, Markus Laube, Jonas Schädlich, Jens Pietzsch, Adrian Nicoara, Luiza Gaina, Sanja Mijatović, Danijela Maksimović-Ivanić, Goran N. Kaluđerović, Evamarie Hey-Hawkins

**Affiliations:** 1 Institute of Bioanalytical Chemistry Centre for Biotechnology and Biomedicine (BBZ), Faculty of Chemistry, Leipzig University, Deutscher Platz 5, 04103 Leipzig, Germany; oi25amoz@studserv.uni-leipzig.de; 2 Department of Engineering and Natural Sciences, University of Applied Sciences Merseburg, Eberhard-Leibnitz-Str. 2, 06217 Merseburg, Germany; 3 Institute for Biological Research “Siniša Stanković”, National Institute of Republic of Serbia, University of Belgrade, 11060 Belgrade, Serbiavuk.gordic@ibiss.bg.ac.rs (V.G.); sanjamama@ibiss.bg.ac.rs (S.M.); nelamax@ibiss.bg.ac.rs (D.M.-I.); 4 Department of Radiopharmaceutical and Chemical Biology, Institute of Radiopharmaceutical Cancer Research, Helmholtz-Zentrum Dresden-Rossendorf (HZDR), Bautzner Landstraße 400, 01328 Dresden, Germany; m.laube@hzdr.de (M.L.); j.schaedlich@hzdr.de (J.S.); j.pietzsch@hzdr.de (J.P.); 5 Faculty of Chemistry and Food Chemistry, School of Science, Technische Universität Dresden, Mommsenstraße 4, 01062 Dresden, Germany; 6 Faculty of Chemistry and Chemical Engineering, Babeş-Bolyai University, Str. Arany Janos Nr. 11, RO-400028 Cluj-Napoca, Romania; adrian.nicoara@ubbcluj.ro (A.N.); ioana.gaina@ubbcluj.ro (L.G.)

**Keywords:** non-steroidal anti-inflammatory drugs (NSAIDs), cyclooxygenase (COX), ruthenacarborane, cyclic voltammetry (CV)

## Abstract

The significant side effects associated with platinum-based anticancer agents have driven the continuous pursuit of novel, non-platinum-based metal compounds. Ruthenium-based organometallic compounds have emerged as promising alternatives, owing to their distinctive and adaptable biochemical properties. The research efforts are focused on the development of ruthenacarborane-based anticancer drugs. The combination of ruthenium(II) complexes, recognized for their inherent anticancer potential, with carboranes, boron-rich clusters possessing unique chemical and physical characteristics, and NSAIDs, known to inhibit COX, an enzyme overexpressed in tumors, offers a novel approach for cancer therapy. Consequently, combining these three moieties into a single molecule represents a compelling strategy to develop drugs with a dual mode of action. Herein, we report the synthesis of a series of ruthenacarborane-(*η*^6^*-p*-cymene)–NSAID conjugates (**4a**, **4b**, **5b**, and **6b**) by linking NSAIDs (flurbiprofen, fenoprofen, and ibuprofen) to ruthenacarborane complexes using methylene and ethylene spacers, while maintaining the integrity of the sensitive ester groups present in the system. The synthesized conjugates were thoroughly characterized using multinuclear (^1^H, ^11^B, and ^13^C) NMR spectroscopy. Notably, the conjugates demonstrated low COX inhibition and no cytotoxic potential against different cancer cell lines, probably due to oxidative deactivation confirmed by cyclic voltammetry (CV). This indicates that the conjugation of this type of ruthenacarborane with NSAIDs does not result in novel anticancer drugs.

## 1. Introduction

Cancer remains a leading cause of mortality [1], with platinum-based anticancer agents, such as cisplatin, carboplatin, and oxaliplatin, serving as the cornerstone of current clinical practice [2,3]. Despite their broad-spectrum activity against various malignancies, the clinical utility of these heavy metal compounds is significantly hampered by severe adverse effects, including gastrointestinal toxicities (nausea, vomiting, diarrhea, abdominal pain), nephrotoxicity, neuromuscular complications, and ototoxicity. Furthermore, the emergence of both primary and acquired resistance in numerous tumor types limits their long-term efficacy [4]. This necessitates a continuous pursuit for novel, non-platinum-based metal compounds capable of expanding the therapeutic landscape of metal-based drugs [5]. Organometallic complexes of Fe^II^, Rh^III^, Ir^III^, Ru^II^, and Os^II^ have demonstrated alternative mechanisms of action compared to cisplatin and its analogs, where DNA is the primary target [6]. Notably, ruthenium-based organometallic compounds have emerged as promising alternatives to conventional platinum-based chemotherapies due to their distinctive and adaptable biochemical properties, although comprehensive data on only a limited number of these agents are currently available [7]. Over the past three decades, extensive research has focused on the synthesis and evaluation of numerous ruthenium-containing agents for their potential antitumor activity [8].

Initial hypotheses regarding ruthenium agents suggested a direct interaction with DNA, similar to platinum; however, significant differences in their mechanisms of action have been observed [9]. Ruthenium possesses three key characteristics that render it theoretically advantageous for medicinal applications. Firstly, its ability to mimic iron in binding to specific biological molecules allows for preferential accumulation in neoplastic tissues over normal tissues [10], potentially facilitated by transferrin-mediated uptake into tumor cells expressing high levels of transferrin receptors [11]. Secondly, the multiple accessible oxidation states (II, III, and IV) of ruthenium enable selective tumor targeting. Ruthenium typically exists in its relatively inert Ru^III^ state systemically but undergoes reduction to the more reactive Ru^II^ state within the tumor microenvironment, which is characterized by reducing properties and increased acidity [12]. This “activation-by-reduction” mechanism not only enhances tumor selectivity but may also confer cytotoxic activity against hypoxic tumors, which are often not accessible by conventional chemotherapy and radiotherapy [13]. Re-oxidation is also theoretically possible for the activated Ru^II^ species to the inactive Ru^III^ form, leading to reduced or no activity.

Finally, certain ruthenium agents have exhibited greater efficacy against cancer metastases compared to primary tumors [14]. This antimetastatic effect is likely mediated through the inhibition of crucial steps in the metastatic cascade, including tumor cell detachment, invasion/migration, and re-adhesion at secondary sites [15]. Given these unique properties, ruthenium is anticipated to exhibit distinct patterns of antitumor activity and clinical toxicity compared to platinum-based drugs. To date, the clinical evaluation of ruthenium-based anticancer agents has seen two prominent candidates reach human trials: NAMI-A, which has demonstrated promising metastasis-inhibitory effects in preclinical models despite limited direct cytotoxicity [16,17], and KP1019, which has shown direct antitumor activity against a broad spectrum of primary human tumor explants through the induction of apoptosis [18,19]. These advancements underscore the growing interest and potential of ruthenium complexes in the development of novel cancer therapeutics.

Conversely, boron, situated to the left of carbon in the periodic table, shares carbon’s capacity for unlimited covalent self-bonding, catenation, leading to the formation of stable molecular structures. Notably, the twelve-vertex *closo*-dicarbadodecaborane (carborane, C_2_B_10_H_12_) in its *ortho*-, *meta*-, or *para*-isomeric forms, represents one of the most chemically and biologically stable molecular compounds known, and its metal complexes are well-established scaffolds in medicinal inorganic chemistry [20]. The most extensively investigated application of carboranes in medicine is their role as high-boron delivery agents for boron neutron capture therapy (BNCT) [21,22], followed by their use as pharmacophores in drug design [23]. Metallacarboranes, including neutral half-sandwich complexes [M(C_2_B_9_H_11_)L] (M = metal ion, where L resembles anionic (M = M^3+^) and/or neutral ligands (M = M^2+^)) and anionic sandwich metallabisdicarbollides [M(C_2_B_9_H_11_)_2_]^−^ (M = M^3+^), are derivatives of boron hydrides that incorporate carbon and metal atoms within their structure [20]. A wide array of metals has been integrated as cluster vertices in metallacarboranes, preserving the chemical and self-assembly properties of the *closo*-carborane clusters while incorporating the redox properties of the metal center.

**Figure 1:**
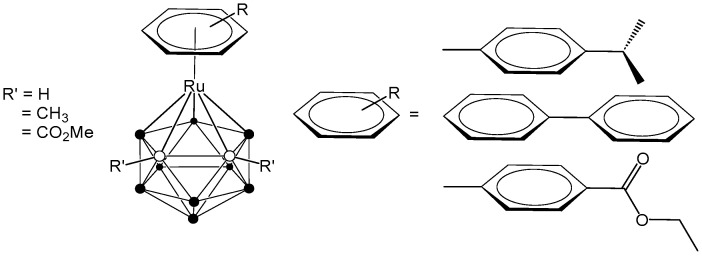
Structure of arene-ruthenacarboranes [24,25,26].

The combination of ruthenium complexes, recognized for their inherent anticancer potential, and carboranes, boron-rich clusters with unique chemical and physical characteristics, presents several promising avenues for the development of innovative cancer therapies. Thus, significant and ongoing research focuses on the development of ruthenacarborane-based anticancer drugs. One example is the coordination of the bulky, electron-poor carborane ligand 7,8-dicarba-*nido*-undecaborane(1-) to an arene-ruthenium metal complex fragment, which has led to the isolation of stable 16-electron complexes [3-(*η*^6^-arene)-1,2-R’_2_-3,1,2-RuC_2_B_9_H_11_] [24,25,26]. Our research group has focused on the synthesis of ruthenacarborane-based complexes, systematically varying the arene ligands coordinated to ruthenium and incorporating either non-polar (R’ = Me) or polar (R’ = CO_2_Me) substituents at the cluster carbon atoms, with the aim of exploring their potential for medical applications (Figure 1) [25,26].

**Figure 2:**
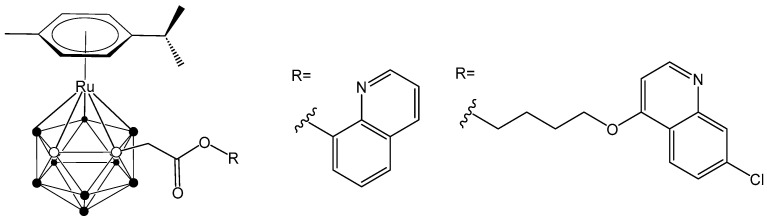
Structure of quinoline-conjugated ruthenacarboranes [24,27].

Furthermore, novel hybrid molecules were designed, containing a ruthenacarborane fragment conjugated with a known modulator of autophagy, namely a quinoline derivative (Figure 2), which showed a dual mode of action against the human glioblastoma cell line LN229 [24]. These encouraging results have inspired our current efforts to develop new conjugates incorporating various anti-inflammatory agents, aiming to further enhance their therapeutic potential.

**Scheme 1:**
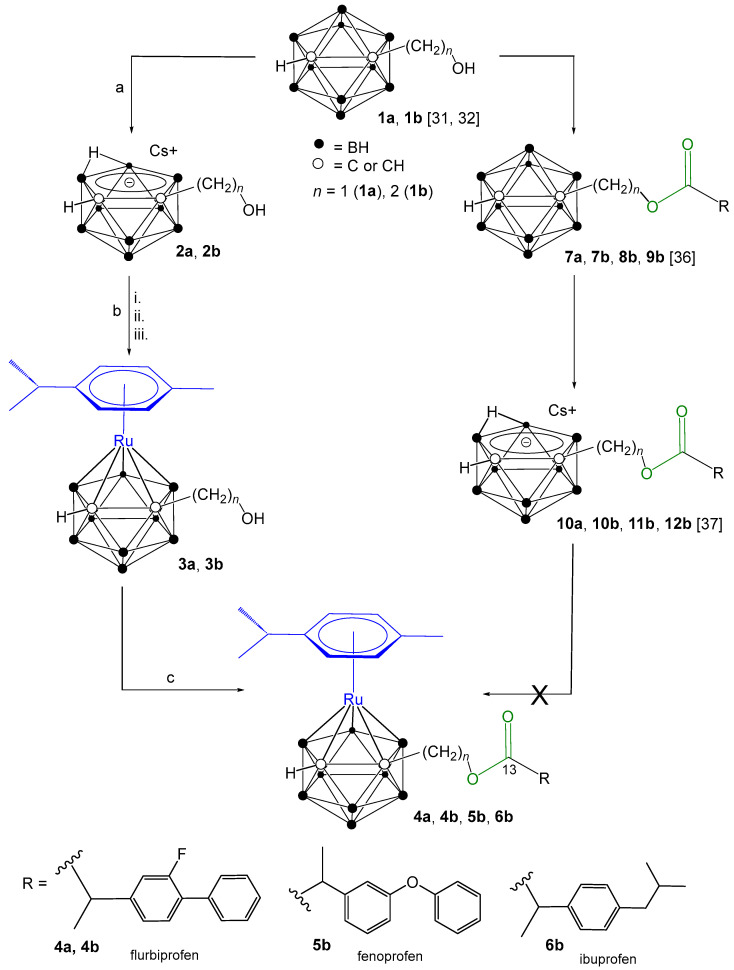
Synthetic approaches to target ruthenacarborane-(*η*^6^*-p*-cymene)–NSAID conjugates **4a**, **4b**, **5b** and **6b**, from *closo*-carborane derivatives (**1a**, **1b**) [31,32]. (a) CsF (2 equiv.), ethanol/THF (2:1 *v*/*v*), reflux, 21 h. (b) (i) TlOEt (3.0 equiv.), THF, −35 °C to rt, 2 h; (ii) [{(*η*^6^*-p*-cymene)RuCl(*μ*-Cl)}_2_] (0.5 equiv.), dry DCM, −65 °C to rt, 18 h; (iii) HCl_aq_. (c) NSAIDs (1 equiv.), *O*-(7-azabenzotriazol-1-yl)-*N*,*N*,*N*′,*N*′-tetramethyluronium hexafluorophosphate (HATU) (2 equiv.), *N*,*N*-diisopropylethylamine (DIPEA) (3.8 equiv.), dry DCM, rt, 48 h.

Non-steroidal anti-inflammatory drugs (NSAIDs) represent a prevalent class of therapeutics widely employed for the management of fever, pain, and inflammation [28,29]. Following the introduction of ibuprofen (Scheme 1), the first compound of the propionic acid sub-series, in 1969, flurbiprofen (Scheme 1) was subsequently developed [30], demonstrating anti-inflammatory, antipyretic, and peripheral analgesic effects across a spectrum of rheumatic conditions.

Fenoprofen (Scheme 1) emerged as a potential therapeutic alternative for patients exhibiting intolerance to aspirin or other anti-inflammatory agents [33,34]. In the 1970s, Vane and colleagues elucidated the mechanism of action of these drugs, revealing their ability to inhibit prostaglandin (PG) biosynthesis through the blockade of cyclooxygenase (COX) [35]. The COX-inhibiting properties of NSAIDs offer a promising strategy for the selective targeting and destruction of COX-overexpressing cancer cells while minimizing off-target effects on healthy tissues.

Our prior research detailed the synthesis of conjugates comprising *ortho*-carborane [36] and *nido*-carborane [37] linked to the NSAIDs ibuprofen, flurbiprofen, and fenoprofen via ester linkages. Building upon this foundation, the current report describes the synthesis of novel ruthenacarborane-(*η*^6^*-p*-cymene)–NSAID (flurbiprofen, fenoprofen and ibuprofen) conjugates designed to elicit a dual mode of action. Specifically, NSAIDs were conjugated through the electron-withdrawing carbon atoms of the carborane cage to the ruthenacarborane-(*η*^6^*-p*-cymene) moiety via ester bonds incorporating methylene or ethylene spacers. The resulting conjugates were then evaluated for their cyclooxygenase (COX) inhibition potential and their antitumor activity against a panel of human cancer cell lines.

## 2. Results and Discussion

### 2.1. Synthesis and Characterization of Ruthenacarborane-(η^6^-p-cymene)–NSAID Conjugates

A series of ruthenacarborane-(*η*^6^*-p*-cymene)–NSAID conjugates (**4a**, **4b**, **5b** and **6b**, Scheme 1) were synthesized by linking NSAIDs (flurbiprofen, fenoprofen, and ibuprofen) to ruthenacarborane complexes via methylene and ethylene spacers. *closo*-Carborane-hydroxyalkyl precursors (**1a**, **1b**), synthesized according to established procedures [31,32], served as starting materials. An initial synthetic strategy, involving direct conjugation of NSAIDs to *closo*-carboranes (**7a**, **7b**, **8b**, **9b**) followed by conversion to *nido*-carborane–NSAIDs (**10a**, **10b**, **11b**, **12b**) with CsF, was attempted. Both *closo*- and *nido*-carborane–NSAID conjugates are reported in our publications [36,37]. However, during the reaction of *nido*-carborane–NSAID conjugates (**10a**, **10b**, **11b**, **12b**) with [{(*η*^6^*-p*-cymene)RuCl(*μ*-Cl)}_2_], the ester linkage in the *nido*-carborane conjugates proved to be unstable, leading to ester bond cleavage (Scheme S1, Supporting Information). Alternative synthetic routes were explored, but they were unsuccessful (see Supporting Information for details, Scheme S2). Another synthetic route employed cesium 7-hydroxymethyl- (**2a**) or cesium 7-hydroxyethyl-*nido*-carborane (**2b**), which were synthesized from **1a**, **1b** via deboronation using CsF in ethanol/THF (2:1 *v*/*v*), achieving high yields (94–96%) [31,32]. Ruthenacarborane complexes (**3a**, **3b**) were subsequently obtained via salt-metathesis from the dithallium salts of **2a**, **2b** and [{(*η*^6^*-p*-cymene)RuCl(*μ*-Cl)}_2_], albeit with low yields (24–33%) following extensive column chromatographic purification. Interestingly, both the *closo*-carborane derivatives **1a**, **1b** and ruthenacarborane complexes **3a**, **3b**, which are both classified as *closo* species, could be activated under analogous conditions (Scheme 1), to form an ester bond. The conjugation of **3a** and **3b** with NSAIDs was accomplished using the coupling reagent HATU (*O*-(7-azabenzotriazol-1-yl)-*N*,*N*,*N′*,*N′*-tetramethyluronium hexafluorophosphate) and *N*,*N*-diisopropylethylamine (DIPEA) as base, yielding the desired products (**4a**, **4b**, **5b**, and **6b**) in moderate yields (52–62%). Purity of all compounds (>95%) was confirmed by high-performance liquid chromatography (HPLC). Structural elucidation of all synthesized compounds was achieved using multinuclear (^1^H, ^11^B, ^13^C) NMR spectroscopy (Figures S1–S24). The conversion of **1a**, **1b** to **2a**, **2b** was evidenced by downfield shifts in the ^1^H NMR spectra and the appearance of a characteristic resonance at −2.58 to −2.85 ppm, confirming *nido*-carborane formation. In the ^11^B{^1^H} NMR spectra of **2a** and **2b**, two broad singlets which shifted from −1.9 to −33.2 ppm and from −5.4 to −37.4 ppm, along with a broad multiplet between −11.2 and −22.9 ppm, further proved the formation of the *nido*-carborane conjugates. The ^13^C{^1^H} NMR spectra of **2a** and **2b** showed shielding of the cluster carbon atoms (C_cluster_), as they gain electron density in the *nido*-caborate precursors. Consequently, both signals move upfield relative to the *closo*-carborane analogues, further confirming the *nido*-cluster formation. Reversion to the *closo* structure in ruthenacarboranes **3a** and **3b** was confirmed by upfield shifts in both the ^11^B{^1^H} and ^13^C{^1^H} NMR spectra, a major and clearly visible upfield shift of the resonance at 209 ppm to 55 ppm (C_cluster_) in the ^13^C{^1^H} NMR spectrum. For the final ruthenacarborane-(*η*^6^*-p*-cymene)–NSAID conjugates (**4a**, **4b**, **5b**, and **6b**), all resonances of the NSAID moieties flurbiprofen (**4a** and **4b**), fenoprofen (**5b**), and ibuprofen (**6b**) were observed in the ^1^H and ^13^C{^1^H} NMR spectra; the carbonyl carbon atom of the ester moiety was observed between 172.5 and 174.6 ppm and hence shifted by ~7 ppm in comparison to the free carboxylic acid of NSAIDs (i.e., ~180 ppm [38]) confirming the formation of ester bonds. The BH signals can be observed in the range between 1.5 and 3.5 ppm in the ^1^H NMR spectra, but, as usually observed for carboranes, are partially not completely resolved due to extensive overlap caused by the large couplings to the boron isotopes ^10^B (*I* = 3, 20%) and ^11^B (*I* = 3/2, 80%).

### 2.2. Cyclic Voltammetry

**Figure 3:**
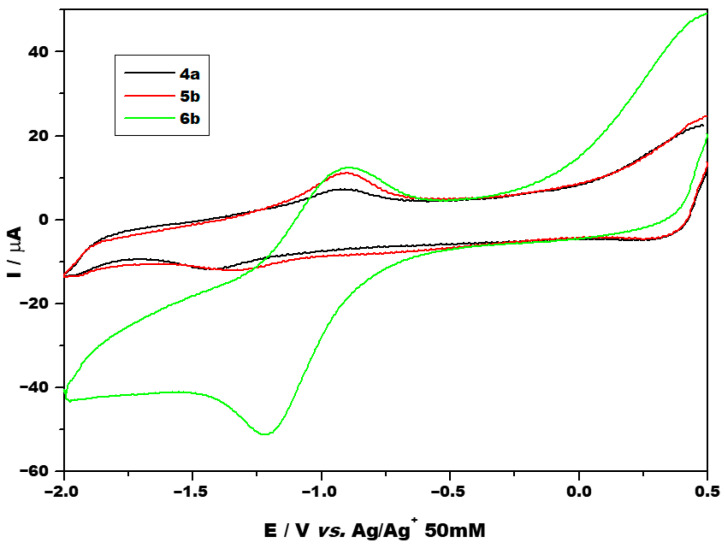
CV of investigated compounds at 0.1 V/s between the potential range of −2 and 0.5 V.

Cyclic voltammetry was performed at various scan rates ranging from 0.05 to 0.25 V/s using different potential ranges. Figure 3 shows the voltammograms obtained using a narrower potential range for the investigated compounds **4a**, **5b** and **6b** at a scan rate of 0.1 V/s. Voltammograms obtained at other scan rates and using a larger potential range are presented in the Supporting Information (Figures S30 and S31). Selected cyclic voltammetry parameters of the complexes are summarized in Table 1, and the half-wave potential was approximated by averaging the peak potentials.

All three compounds showed one redox couple, at potentials close to −1 V, which was attributed to the Ru^II^/Ru^III^ system. Additional anodic and cathodic processes were also present as irreversible chemical peaks, all of which were ascribed to ligand redox activity. No evidence of a Ru^III^/Ru^IV^ redox couple was obtained. In the literature, oxidation of Ru^III^ has been reported to take place at potentials greater than 2 V compared to the oxidation of Ru^II^ [39]. As peak separations (ΔE_p_) exceed 212 mV, peaks are considered to be irreversible electrochemically. This proves a slow charge transfer at the metal core, estimated values of the standard rate constant (k^o^) are of the order of magnitude of 10^−5^ cm/s. As mentioned in the introduction, one reason for selecting ruthenium as the metal core is its potential prodrug behavior. Under physiological conditions, ruthenium typically exists in the relatively biologically inert Ru^III^ oxidation state, and more biologically active Ru^II^ forms within the tumor microenvironment [12]. However, for the investigated compounds, Ru^II^ undergoes oxidation to the less biologically active Ru^III^ state, reduction takes place at a too negative potential, impeding the formation of the biologically active Ru^II^ state. As comparison, a platinum(IV) compound with good antitumor activity (iproplatin) exhibits a cathodic peak at approximately 0.51 V vs. Ag/Ag^+^ 50 mM corresponding to the formation of biologically active Pt^2+^, while a poorly performing platinum(IV) prodrug (tetraplatin) exhibits a cathodic peak at −0.43 V vs. Ag/Ag^+^ 50 mM [40].

### 2.3. Stability Studies

The stability of the conjugate **4a** was monitored with ^1^H NMR spectroscopy in DMSO-*d_6_* over 72 h (Figure S26, Supporting Information). No degradation products could be detected. Dynamic Light Scattering (DLS) analysis showed no differences between dissolved conjugates **4a**, **4b**, **5b**, and **6b**, and the solvent mixture (DMSO+PBS) (Figure S29, Supporting Information), indicating that no aggregation occurred. Furthermore, comparison with 200 nm standard particles revealed that particle counts for all conjugates and the solvent solution were within the background noise range, thus confirming also the absence of aggregates.

The ester group was selected as a linker between carborane and NSAIDs to enhance drug selectivity by exploiting the pH differences between tumor and healthy tissues. Tumor microenvironments typically exhibit a lower pH (4.0–6.5) compared to normal tissues (pH 7.3–7.5) [41,42], allowing for ester bond cleavage and selective drug release. Ester bonds offer sufficient stability under physiological conditions while being susceptible to hydrolysis in acidic environments, thereby enabling targeted release within tumor sites [43,44].

The stability of compounds **4a**, **5b**, and **6b** was evaluated at pH 4 and pH 7 using UV–Vis spectroscopy over 24 and 48 h (Figure S27, Supporting Information). Compounds **4a** and **5b** demonstrated stable ester linkages at pH 7, while significant degradation was observed at pH 4, consistent with the expected acid-labile behavior. In contrast, compound **6b** displayed an unexpected degradation profile, with similar spectral changes observed at both pH 4 and pH 7. This suggests that, unlike the other compounds, **6b** undergoes ester bond hydrolysis even under neutral conditions, indicating atypical instability where stability at pH 7 would normally be anticipated.

In order to corroborate the results obtained, we have conducted further stability studies using UPLC-MS as means of detection. When we conducted the experiments at relevant concentrations of the analytes (final concentration of 100 µM), UV-detection on UPLC at 254 nm was insufficiently sensitive for assessment of the portion of intact compound due to the need of protein precipitation from the media and concomitant further dilution as well as low recovery rate from the media. The latter can be attributed to adhesion of the conjugates to the plastic walls of the Eppendorf tubes in which the incubations took place. Therefore, a gradient to safely separate alcohols **3a** and **3b**, the expected hydrolysis products, from the conjugates **4a**, **5b**, and **6b** was chosen for analysis (see Figures S35–S37 in the Supporting Information). Further, the total ion count chromatogram of each sample was filtered for the molecular ion peaks of the intact compound and the respective alcohol. None of the conditions led to hydrolysis of the conjugates as no molecular ion peak of the corresponding alcohol was detected. The retention time as well as the mass spectrum support this assignment.

While both methods (UV-Vis and UPLC-MS) gave similar results for **4a** and **5b**, a difference in stability of **6b** was observed showing stronger evidence for hydrolysis based on the more intense UV-Vis signal change than observed by weak signals in LC-MS. However, because UV-Vis is not specific, this remains to be elucidated using a more elaborate and quantitative analysis technique like targeted LC-MS/MS. This should be employed in future studies, which was however not in the scope of this study.

### 2.4. COX Inhibitory Potential

The COX inhibition potential and selectivity against ovine COX-1 and human recombinant COX-2 was evaluated for conjugates **4a**, **4b**, **5b**, and **6b** using a fluorescence-based COX assay (Table 2). Initial screening at a concentration of 100 µM revealed unexpectedly low inhibitory activity for all four conjugates. Detailed analysis showed that the starting materials 1-hydroxyalkyl-*closo*-carborane (**1a**, **1b**) exhibited no inhibition against either COX enzyme. Upon conversion to cesium 7-hydroxyalkyl-*nido*-carborane (**2a**, **2b**), a modest increase in inhibition was observed, with a slight preference for COX-2. Additionally, the compound with the longer ethylene spacer (**2b**) demonstrated greater inhibitory potential than its shorter methylene spacer analogue (**2a**). Reconversion of the *nido*-carborane precursors to the *closo* structure, namely formation of ruthenacarboranes **3a** and **3b**, resulted in a decrease in inhibition compared to the *nido* forms, though activity remained above that of the initial *closo* compounds (**1a**, **1b**). The trend of the longer ethylene spacer (**3b**) exhibiting higher inhibition than the shorter methylene spacer (**3a**) was maintained in this series. Following conjugation with NSAIDs, a minimal increase in inhibition was noted. These conjugates unexpectedly displayed more selectivity towards COX-1 rather than COX-2. Furthermore, the longer ethylene spacer (**4b**) showed slightly higher inhibition compared to the shorter methylene spacer (**4a**), a reversal of the activity trend observed with their respective *closo*-carborane–NSAID conjugates (where the shorter methylene spacer (**7a**) was more active than the longer ethylene spacer (**7b**) [36]. Notably, the ruthenacarborane-(*η*^6^-*p*-cymene)−fenoprofen conjugate with an ethylene spacer (**5b**) exhibited no inhibition against either COX enzyme. Overall, the *nido*-carborane–NSAIDs conjugates (**10a**, **10b**, **11b**, and **12b**) demonstrated the highest inhibitory activity among the evaluated compounds [37].

### 2.5. Cytotoxicity, Toxicity and Mechanism of Action

To assess the cytotoxic potential of *nido*-carborane precursors (**2a**, **2b**), ruthenacarborane complexes using methylene and ethylene spacers (**3a**, **3b**) and ruthenacarborane-(*η*^6^-*p*-cymene)–NSAID conjugates (**4a**, **4b**, **5b**, and **6b**), in vitro studies using A375 human melanoma, A549 human lung carcinoma, HCT116 and HT29 human colorectal cancer and MDA-MB-231 breast carcinoma were performed. To define the selectivity toward a cancer phenotype, primary transformed lung embryonal fibroblasts of human origin (MRC-5) were used. The cells were exposed to a wide range of concentrations of each compound. After 72 h treatment, cell viability was measured by two different assays, 3-(4,5-dimethylthiazol-2-yl)-2,5-diphenyltetrazolium bromide (MTT) and crystal violet (CV). Results are presented in Table 3 and Figures S32–S34 (Supporting Information). While the *nido*-carborane precursors (**2a** and **2b**) were not active in the tested dose range, their ruthenacarborane counterparts (**3a** and **3b**) exerted significant cytotoxic potential with similar IC_50_ values in all cell lines despite differences in their origin and intracellular characteristics. Selectivity toward malignant phenotype was moderate and, even in the case of the most sensitive cancer cell lines to the applied treatments, did not reach statistical significance. Ruthenacarborane-(*η*^6^-*p*-cymene)–NSAID (flurbiprofen, fenoprofen and ibuprofen) conjugates (**4a**, **4b**, **5b**, and **6b**) were completely inactive in terms of cytotoxicity against the same cell lines and under the same experimental setting.

Results obtained during cyclic voltammetry show that Ru^III^ reduction occurs at a more negative potential than is ideal for forming biologically active Ru^II^. This may result in a reduction in, or even complete loss of, the pharmacological activity of the conjugated drugs (Table 1). Thus, it can be concluded that ligation with NSAIDs neutralizes the cytotoxic potential of ruthenacarborane complexes **3a** and **3b**, while the COX inhibitory potential of ruthenacarborane-(*η*^6^*-p*-cymene)–NSAID conjugates was also compromised. A likely explanation for this inactivity lies in the structural differences when compared to previously reported active hybrids by Gozzi et al. (2019) [24]. The first conjugate in Figure 2, which demonstrated potent cytotoxicity, featured a direct aryl ester linkage between the ruthenacarborane and the quinoline moiety, enabling both stability and efficient intracellular release. In contrast, the NSAIDs in our conjugates exhibit a CHMe group between the ester group and aromatic moiety of the NSAID, which may reduce target affinity, interfere with cellular uptake, or hinder efficient intracellular release. This hypothesis is further supported by the inactivity of the second conjugate (Figure 2) in Gozzi’s study [24], where the aromatic quinoline moiety is attached to a carborane-CH_2_CO_2_ moiety via an alkylene chain showing diminished activity compared to the directly connected analogue. Taken together, these observations emphasize that not only the choice of pharmacophore, but also the nature and length of the connecting spacer are crucial design parameters in the development of ruthenacarborane-based hybrid drugs.

The comparative evaluation of biological activity between the synthesized *nido*-carborane–NSAID conjugates (**10a**, **10b**, **11b**, and **12b**) and their corresponding free NSAIDs revealed a remarkable and somewhat unexpected trend: the *nido*-carborane derivatives outperformed the ruthenacarborane-(*η*^6^*-p*-cymene)–NSAID conjugates in terms of inhibitory potency, not only in vitro inhibition potential of investigated cancer cells but also as COX inhibitors [36,37]. This finding suggests that the incorporation of a *nido*-carborane cage, even in the absence of a transition metal complex fragment, can impart significant pharmacological advantages.

One possible explanation lies in the intrinsic properties of the *nido*-carborane cluster, which is known to exhibit a net charge of −1, a hydrophobic character, a unique three-dimensional geometry, and enhanced stability under physiological conditions [20,45]. These features may promote stronger interactions with the hydrophobic channel of the COX-2 active site as well as positively charged amino acids like arginine-120, thereby increasing inhibitory efficiency. In addition, the observed higher selectivity for COX-2 over COX-1 is of particular therapeutic importance, as COX-2 selective inhibitors are associated with reduced gastrointestinal side effects compared to non-selective NSAIDs [46,47].

The superior activity of *nido*-carborane–NSAID conjugates over their Ru^II^-based counterparts could also be attributed to differences in steric and electronic effects. The presence of the Ru^II^ center in *η*^6^-*p*-cymene complexes may impose conformational restrictions or influence the electronic distribution in a way that is less favorable for COX-2 binding. In contrast, the *nido*-carborane scaffold provides a compact platform that may better preserve the pharmacophore alignment of the NSAID moiety. These results align with recent studies reporting that metallation of bioactive molecules does not always lead to improved activity [24], and in some cases, purely organoboron-based systems can be equally or more effective.

## 3. Materials and Methods

(*R/S*)-2-[4-(2-methylpropyl)phenyl]propanoic acid (ibuprofen), (*R/S*)-2-(3-phenoxyphenyl)propanoic acid (fenoprofen), (*R/S*)-2-(2-fluorobiphenyl-4-yl)propanoic acid (flurbiprofen), cesium fluoride, and [{(*η*^6^*-p*-cymene)RuCl(μ-Cl)}_2_] were purchased from Sigma Aldrich, thallium(I) ethoxide was purchased from Thermo fFisher Scientific and *ortho*-carborane (C_2_B_10_H_12_) was purchased from Katchem spol. s r. o. 2-(3-Phenoxyphenyl)propanoic acid (fenoprofen) and thallium(I) ethoxide were stored in a refrigerator at 2 °C to 8 °C, protected from light. All other chemical reagents were stored at ambient temperature in a dry environment. Tetrahydrofuran (THF) and dichloromethane (DCM) were dried according to the published procedure [48], and stored over molecular sieve (3 Å) under a nitrogen atmosphere at room temperature. Ethanol was degassed with nitrogen, dried over magnesium, freshly distilled prior to use and stored under nitrogen atmosphere over molecular sieve (4 Å). All other solvents were used without further purification. All syntheses were carried out in a protective atmosphere of argon gas using standard Schlenk techniques; workup was performed in air. Caution: All manipulations involving thallium(I) compounds were performed wearing personal protective equipment as prescribed in the material safety data sheet and thallium(I)-containing waste was disposed according to regulations.

1-(1′-Hydroxymethyl)- and 1-(2′-hydroxyethyl)-1,2-dicarba-*closo*-dodecaborate (**1a**, **1b**) were prepared using established procedures [31,32]; the corresponding *closo*-carborane (**7a**, **7b**, **8b**, **9b**) and *nido*-carborane conjugates (**10a**, **10b**, **11b**, **12b**) with NSAIDs (ibuprofen, flurbiprofen and fenoprofen) were synthesized according to our previously reported procedure [36,37]. As described above, during the reaction of the *nido*-carborane conjugates with [{(*η*^6^*-p*-cymene)RuCl(*μ*-Cl)}_2_], the ester linkage proved to be unstable, resulting in cleavage of the ester bond. Consequently, alternative synthetic approaches were developed, as outlined in Scheme 1. The structures of all synthesized compounds were confirmed by ^1^H, **^11^**B and ^13^C NMR spectroscopy. Reaction progress was monitored using thin-layer chromatography (TLC) on pre-coated silica plates with a fluorescence indicator (Merck Silica Gel 60 F_254_), using ethyl acetate and *n*-hexane in varying volume ratios as the mobile phase. Visualization of the compounds was performed under UV light or by dipping the TLC plates in a PdCl_2_ solution (1 wt% in MeOH), followed by gentle heating with a heat gun. High-performance liquid chromatography (HPLC) at 254 and 220 nm confirmed that compounds except **3a** and **3b** exhibited a purity of >95% (Table S1, Supporting Information). NMR spectra were recorded at room temperature using a Bruker AVANCE III HD 400 spectrometer. ^1^H (400.13 MHz) and ^13^C (100.16 MHz) NMR spectra were referenced to tetramethylsilane (TMS) as an internal standard. **^11^**B (128.38 MHz) NMR spectra were referenced according to the unified Ξ-scale [49]. The numbering scheme for NMR assignments of compounds **2a**, **2b**, **3a**, **3b**, **4a**, **4b**, **5b**, and **6b** is provided in the Supporting Information (Figures S1–S24).

### 3.1. General Synthesis of Nido-Carborane Precursors (***2a*** and ***2b***)

A reflux reaction was conducted for 24 h by combining *closo*-carborane–hydroxyalkyl **1a** or **1b** (1.0 mmol, 1 equiv.) with CsF (304 mg, 2.0 mmol, 2 equiv.) in 30 mL of an ethanol/THF mixture (2:1 *v*/*v*). After completion of the reaction, the mixture was allowed to cool to room temperature, and the precipitated cesium salt was separated via filtration. The remaining solvent was evaporated, yielding the first fraction of crude compounds as white solids. The crude product was subsequently suspended in acetone, followed by filtration and three successive washings with acetone. The collected acetone washings were evaporated to obtain a second fraction of *nido*-carborane precursor as white solid. Further purification was achieved by washing with a small amount of DCM to remove any residual impurities. The final purified product was dried under vacuum. The resulting compounds, **2a** or **2b** (white solids), were found to be soluble in acetone and DMSO but insoluble in DCM, chloroform, and *n*-hexane.

**Cesium 1-(1′-hydroxymethyl)-*nido*-carborane (2a):** Yield 278 mg (94%). ^1^H NMR (400 MHz, acetone-d_6_): δ 3.64 (d, *J* = 11.3 Hz, 2H, H_1_), 1.83 (s, 1H, *OH*), −2.58 (s, 1H, C_cluster_-H). ^13^C{^1^H} NMR (101 MHz, acetone-d_6_): δ 209.2 (C*_cluster_*), 69.3 (C*_cluster_*-H), 64.8 (C_1_). ^11^B{^1^H} NMR (128 MHz, acetone-d_6_): δ −10.5 (s, 1B), −11.5 (s, 1B), −15.4 (s, 1B), −17.4 (s, 1B), −18.8 (s, 2B), −22.5 (s, 1B), −33.2 (s, 1B), −37.5 (s, 1B).

**Cesium 1-(2′-hydroxyethyl)-*nido*-carborane (2b):** Yield 297 mg (96%). ^1^H NMR (400 MHz, acetone-d_6_): δ 3.47 (dm, *J* = 38.5 Hz, 2H, H_2_), 1.85 (m, 2H, H_1_), 1.5 (1H, s, *OH*) −2.59 (s, C_cluster_-H). ^13^C{^1^H} NMR (101 MHz, acetone-d_6_): δ 208.7 (C*_cluster_*), 68.3 (C*_cluster_*-H), 63.1 (C_2_), 42.7 (C_1_). ^11^B{^1^H} NMR (128 MHz, acetone-d_6_): δ −11.1 (s, 2B), −14.2 (s, 1B), −16.5 (s, 1B), −18.9 (s, 2B), −21.9 (s, 1B), −33.3 (s, 1B), −37.4 (s, 1B).

### 3.2. General Synthesis of Ruthenacarborane ([Ru(η^6^-p-cymene)-closo-1-(CH_2_)_n_OH-C_2_B_9_H_10_], (n = 1, 2))

#### 3.2.1. Deprotonation of the *Nido*-Carborane Precursors

A light-protected setup was established, and *nido*-carborane precursors **2a** or **2b** (0.66 mmol, 1.0 equiv.) were dissolved in dry THF (10 mL) under an argon atmosphere. The solution was then cooled to −35 °C, followed by the addition of TlOEt (0.14 mL, 1.99 mmol, 3.0 equiv.) in a single portion, leading to the immediate precipitation of a bright yellow solid. The reaction mixture was stirred at −35 °C for 30 min and then allowed to reach room temperature, where stirring continued for an additional 1.5 h. Upon completion, stirring was halted, and the supernatant THF solution was filtered under an argon atmosphere. The resulting residue was washed sequentially with dry ethanol (8 mL) and *n*-hexane (5 mL). The bright yellow solid was subsequently dried under vacuum for 3 h and used directly without further purification.

#### 3.2.2. Formation of Ruthenacarborane (**3a** and **3b**)

The dicarbollide derivatives synthesized according to Section 3.2.1. (1.40 mmol, 1.0 equiv.) were subsequently combined with [{(*η*^6^*-p*-cymene)RuCl(*μ*-Cl)}_2_] (283 mg, 0.46 mmol, 0.33 equiv.) at −65 °C under an argon atmosphere, protected from light. Dry DCM (8 mL) was added to the reaction mixture, resulting in a red-orange solution, which was stirred for 18 h while gradually warming to room temperature. Following this, aqueous HCl (20 vol %, 0.1 mL) was introduced, and the mixture was stirred for an additional 40 min. Celite was then added, and the volatiles were removed under reduced pressure. The crude residue was purified by column chromatography on silica gel (column dimensions: length = 5 cm, diameter = 2.5 cm) under ambient conditions. A solvent gradient of *n*-hexane/ethyl acetate/acetic acid (1:1:0.01 (*v*/*v*/*v*) to 1:3:0.01 (*v*/*v*/*v*) to acetone) was used for elution. Compounds **3a** and **3b** were obtained from two separate reactions and purified individually by column chromatography. For compound **3a**, a yellow band (R_f_ = 0.32) was eluted using an *n*-hexane/ethyl acetate (1:3, *v*/*v*) system and contained the pure product. For compound **3b**, a similar yellow band (R_f_ = 0.28) was collected using the same solvent system. In both cases, a slower-moving red-orange band (R_f_ ≈ 0.09) was also observed, containing a mixture of the *nido*-carborane precursor and [{(*η*^6^-*p*-cymene)RuCl(*μ*-Cl)}_2_].The fraction containing **3a** or **3b** was obtained as an air-stable yellow-brown solid. exhibiting good solubility in acetone, THF, acetonitrile, DMSO, chloroform, and dichloromethane.

**[Ru(*η*^6^-*p*-cymene)-*closo*-1-(CH_2_)OH-C_2_B_9_H_10_] (3a):** (Yield 24%, 135 mg). ^1^H NMR (400 MHz, CDCl_3_): δ 5.97 (dd, *J* = 2.5 Hz, 4H, H_1_-H_6_), 3.95 (d, 2H, H_11_), 3.91 (s, 1H, C_Cluster_-H), 2.97–2.77 (m, 1H, H_7_), 2.34 (s, 3H, H_10_), 1.36–1.21 (m, 6H, H_8_ and H_9_), 3.15–1.15 (b, 9H, B_9_H_9_). ^13^C{^1^H} NMR (101 MHz, CDCl_3_): δ 111.7 (C_1_), 101.7 (C_4_), 89.3 (C_2_ and C_6_), 86.4 (C_3_ and C_5_), 72.0 (C_cluster_-H), 47.5 (C_cluster_), 31.6 (C_11_), 29.7 (C_7_), 22.8 (C_8_ and C_9_), 19.1 (C_10_). ^11^B{^1^H} NMR (128 MHz, CDCl_3_): δ 1.8 (s, 1B), 0.8 (s, 1B), −3.7 (s, 1B), −4.8 (s, 1B), −9.8 (s, 1B), −10.9 (s, 1B), −16.8 (s, 1B), −18.7 (s, 1B), −20.0 (s, 1B).

**[Ru(*η*^6^-*p*-cymene)-*closo*-1-(CH_2_)*_2_*OH-C_2_B_9_H_10_] (3b):** (Yield 33%, 190 mg). ^1^H NMR (400 MHz, CDCl_3_): δ 5.98–5.82 (m, 4H, H_1–6_), 3.95 (s, 1H, C_cluster_-H), 3.82–3.68 (m, 2H, H_12_), 2.94 (hept, 1H, *J* = 6.9 Hz, H_7_), 2.65 (dd, 2H, *J* = 7.7, 6.6 Hz, H_11_), 2.41 (s, 3H, H_10_), 1.36 (dd, 6H, *J* = 6.9, 5.0 Hz, H_8_ and H_9_), 3.15–1.15 (b, 9H, B_9_H_9_). ^13^C{^1^H} NMR (101 MHz, CDCl_3_): δ 112.1 (C_1_), 102.1 (C_4_), 92.2, 91.0 (C_2_, C_6_), 88.9, 88.6 (C_3_, C_5_), 71.6 (C_cluster_-H), 61.9 (C_12_), 56.5 (C_cluster_), 49.2 (C_11_), 31.9 (C_7_), 23.6 (C_8_ or C_9_), 20.7 (C_8_ or C_9_), 18.8 (C_10_). ^11^B{^1^H} NMR (128 MHz, CDCl_3_): δ 1.8 (s, 1B), 1.0 (s, 1B), −4.1 (s, 1B), −7.8 (s, 2B), −10.2 (s, 1B), −16.4 (s, 1B), −18.3 (s, 1B), −19.5 (s, 1B).

### 3.3. General Synthesis of Ruthenacarborane-(η^6^-p-cymene)–NSAID Conjugates ([Ru(η^6^-p-cymene)-closo-C_2_B_9_H_10_-(CH_2_)_n_-NSAID] (n = 1, 2)) (***4a***, ***4b***, ***5b***, and ***6b***)

A turbid solution of NSAIDs (ibuprofen, flurbiprofen or fenoprofen) (0.13 mmol, 1 equiv.) and coupling agent HATU (103 mg, 0.27 mmol, 2 equiv.) in dry DCM was prepared at room temperature. The addition of DIPEA (90 μL, 0.51 mmol, 3.8 equiv.) as a base caused the solution to turn pale yellow. After allowing the mixture to stir for 5 min, **3a** or **3b** (1.32 mmol, 1.0 equiv.) was introduced. The reaction mixture was then stirred at room temperature for 48 h to achieve maximum conversion. The precipitate derived from the coupling reagent was removed by filtration. The crude product was purified via column chromatography using a gradient of 10–30% EtOAc in *n*-hexane. Subsequent solvent evaporation yielded compounds **4a**, **4b**, **5b**, and **6b** as air-stable yellow solids. These products demonstrated good solubility in acetone, THF, acetonitrile, DMSO, chloroform, and dichloromethane.

**[Ru(*η*^6^*-p*-cymene)-1-{methyl (*R/S*)-2-[4-(2-fluorobiphenyl-4-yl)propanoic acid ester]-*closo*-C_2_B_9_H_10_}] (4a):** (Yield 61%, 50 mg). ^1^H NMR (400 MHz, CDCl_3_): δ 7.48–7.03 (m, 8H, H_16–27(biphenyl)_), 5.85–5.58 (m, 4H, H_1–6_), 4.32 (dddd, *J* = 15.4, 12.0 Hz, 2H, H_11_), 3.83 (s, 1H, C_Cluster_-H), 3.72 (q, *J* = 6.9 Hz, 1H, H_14_), 2.75 (m, 1H, H_7_), 2.23 (d, *J* = 7.2 Hz, 3H, H_10_), 1.50 (dd, *J* = 7.1, 1.5 Hz, 3H, H_15_), 1.28–1.16 (m, 6H, H_8_ and H_9_), 3.80–0.09 (b, 9H, B_9_H_9_). ^13^C{^1^H} NMR (101 MHz, CDCl_3_): δ 173.0 (C_13_), 160.9 (C_18_), 158.5 (C_22_), 141.4 (C_16_), 135.3 (C_19_), 131.4 (C_20_), 128.9 (C_23_ or C_27_), 128.4 (C_24_ or C_26_), 127.8 (C_25_), 123.7 (C_21_), 115.5 (C_17_), 112.4 (C_1_), 102.4 (C_4_), 91.1 (C_2_ or C_6_), 90.4 (C_2_ or C_6_), 88.2 (C_3_ or C_5_), 87.9 (C_3_ or C_5_), 70.7 (C_cluster_-H), 56.0 (C_cluster_), 49.6 (C_11_), 44.9 (C_14_), 31.5 (C_7_), 22.7 (C_8_ or C_9_), 22.8 (C_8_ or C_9_), 18.7 (C_10_), 18.1 (C_15_). ^11^B{^1^H} NMR (128 MHz, CDCl_3_): δ −2.4 (s, 1B), −2.7 (s, 1B), −7.8 (s, 1B), −9.0 (s, 1B), −10.3 (s, 1B), −16.8 (s, 1B), −18.1 (s, 1B), −19.4 (s, 1B), −22.2 (s, 1B).

**[Ru(*η*^6^*-p*-cymene)-2-{ethyl (*R/S*)-2-[4-(2-fluorobiphenyl-4-yl)propanoic acid ester]-*closo*-C_2_B_9_H_10_}] (4b):** (Yield 62%, 51 mg). ^1^H NMR (400 MHz, CDCl_3_): δ 7.56–7.07 (m, 8H, H_16–27(biphenyl)_), 6.01–5.88 (m, 4H, H_1–6_) 4.30 (dtd, *J* = 18.7, 10.5, 5.7 Hz, 1H, H_12_), 4.03 (tdd, *J* = 10.5, 8.1, 5.6 Hz, 2H, H_12_), 3.83 (s, 1H, C_Cluster_-H), 3.73 (q, *J* = 7.1 Hz, 1H, H_14_), 2.86 (hept, *J* = 6.8 Hz, 1H, H_7_), 2.68–2.41 (m, 2H, H_11_), 2.32 (s, 3H, H_10_), 1.53 (d, *J* = 1.4 Hz, 3H, H_15_), 1.28 (dd, *J* = 6.6 Hz, 6H, H_8_ and H_9_), 3.80–0.80 (b, 9H, B_9_H_9_). ^13^C{^1^H} NMR (101 MHz, CDCl_3_): δ 174.1 (C_13_), 160.9 (C_18_), 158.5 (C_22_), 141.8 (C_16_), 135.0 (C_19_), 130.9 (C_20_), 128.8 (C_23_ and C_27_), 128.4 (C_24_ and C_26_), 127.9 (C_25_), 124.0 (C_21_), 115.0 (C_17_), 111.9 (C_1_), 101.9 (C_4_), 91.9 (C_2_), 90.7 (C_6_), 88.1, 88.1 (C_3_, C_5_), 74.7 (C_cluster_-H), 64.4 (C_12_), 55.3 (C_cluster_), 45.5 (C_11_), 43.8 (C_14_), 31.5 (C_7_), 23.0 (C_8_), 22.4 (C_9_), 18.7 (C_10_), 18.6 (C_15_). ^11^B{^1^H} NMR (128 MHz, CDCl_3_): δ 1.1 (s, 2B), −4.4 (s, 1B), −7.7 (s, 2B), −10.0 (s, 1B), −16.4 (s, 2B), −18.4 (s, 1B).

**[Ru(*η*^6^*-p*-cymene)-2-{ethyl (*R/S*)-2-(3-phenoxyphenyl)propanoic acid ester]-*closo*-C_2_B_9_H_10_}] (5b):** (Yield 56%, 46 mg). ^1^H NMR (400 MHz, CDCl_3_): δ 7.33–6.78 (m, 9 H, H_16–27-(phenoxybenzene)_), 5.97–5.79 (m, 4H, H_1–6_), 4.21 (ddt, *J* = 26.9, 10.5, 5.6 Hz, 2H, H_12_), 3.94 (dq, *J* = 17.0, 5.2 Hz, 1H, H_14_), 3.83 (s, 1H, C_Cluster_-H), 2.80 (h, *J* = 7.0 Hz, 1H, H_7_), 2.27 (s, 3H, H_10_), 1.43 (d, *J* = 7.2 Hz, 3H, H_15_), 1.23 (dt, *J* = 19.8, 6.8 Hz, 6H, H_8_ and H_9_), 4.60–0.50 (b, 9H, B_9_H_9_). ^13^C{^1^H} NMR (101 MHz, CDCl_3_): δ 174.6 (C_13_), 157.7 (C_18_), 157.1 (C_22_), 142.4 (C_16_), 130.2 (C_20_), 130.0 (C_24_ and C_26_), 123.6 (C_21_), 122.4 (C_25_), 119.0 (C_23_ or C_27_), 118.9 (C_19_), 117.7 (C_17_), 112.3 (C_1_), 102.3 (C_4_), 92.3 (C_2_), 91.3 (C_6_), 89.1, 88.8 (C_3_, C_5_), 70.7 (C_cluster_-H), 63.6 (C_12_), 60.7 (C_cluster_), 55.2 (C_11_), 45.5 (C_14_), 31.7 (C_7_), 23.1 (C_8_), 22.8 (C_9_), 19.0 (C_10_), 18.4 (C_15_). ^11^B{^1^H} NMR (128 MHz, CDCl_3_): δ 1.7 (s, 1B), −7.2 (s, 1B), −8.3 (s, 1B), −9.6 (s, 1B), −10.6 (s, 1B), −13.2 (s, 1B), −14.1 (s, 1B), −18.0 (s, 1B), −19.1 (s, 1B).

**[Ru(*η*^6^*-p*-cymene)-2-{ethyl (*R/S*)-2-[4-(2-propylphenyl)propanoic acid ester]-*closo*-C_2_B_9_H_10_}] (6b):** (Yield 52%, 40 mg). ^1^H NMR (400 MHz, CDCl_3_): δ 7.17 (ddd, *J* = 32.2, 8.1, 2.4 Hz, 4H, H_16–19_), 6.03–5.89 (m, 4H, H_1–6_), 4.34–4.23 (m, 2H, H_12_), 4.07–3.96 (m, 2H, H_12_), 3.84 (s, 1H, C_Cluster_-H), 3.70 (q, *J* = 7.0 Hz, 1H, H_14_), 2.88 (pd, *J* = 7.0, 4.9 Hz, 1H, H_7_), 2.64–2.52 (m, 1H, H_11_), 2.47 (d, *J* = 7.2 Hz, 2H, H_22_), 2.34 (s, 3H, H_10_), 1.87 (dqd, *J* = 12.0, 7.1, 3.7 Hz, 1H, H_23_), 1.51 (dd, *J* = 7.2, 1.6 Hz, 3H, H_15_), 1.36–1.27 (m, 6H, H_8_ and H_9_), 0.91 (d, *J* = 2.0 Hz, 6H, H_24_ and H_25_), 4.02–0.70 (b, 9H, B_9_H_9_). ^13^C{^1^H} NMR (101 MHz, CDCl_3_): δ 171.7 (C_13_), 140.7 (C_19_), 136.7 (C_16_), 129.3 (C_20_ and c_18_), 127.2 (C_17_ and C_21_), 112.1 (C_1_), 102.0 (C_4_), 92.2 (C_2_), 91.5 (C_6_), 89.0, 88.6 (C_3_, C_5_), 71.3 (C_cluster_-H), 63.2 (C_12_), 61.8 (C_cluster_), 55.0 (C_11_), 45.0 (C_22_), 44.8 (C_14_), 31.6 (C_7_), 30.1 (C_23_), 23.1 (C_8_), 22.3 (C_9_), 18.8 (C_10_), 18.5 (C_15_). ^11^B{^1^H} NMR (128 MHz, CDCl_3_): δ 1.1 (s, 2B), −4.4 (s, 1B), −7.8 (s, 2B), −10.1 (s, 1B), −16.3 (s, 1B), −18.6 (s, 2B).

### 3.4. Purity Analysis by High Performance Liquid Chromatography (HPLC)

Analytical HPLC was performed on an Agilent 1200 Series (Agilent Technologies, Santa Clara, CA, USA) system consisting of interface 35900E, quaternary pump G1311A, degasser G1322A, autosampler G1329A, thermostatted column compartment G1316A, and diode array detector G1315D, equipped with a gamma detector GABI (Elysia-raytest GmbH, Straubenhardt, Germany) and Purospher^®^ RP-18e (5 µm) LiChroCART^®^ 125-3 column. A binary gradient system of 0.1% *v*/*v* CF_3_COOH in H_2_O (A) and CH_3_CN (B) at 40 °C was used. Data was processed using OpenLAB CDS ChemStation Edition version C.01.07 SR1. Gradients (%B): *t*_0 min_ 5–*t*_0.5 min_ 5–*t*_7 min_ 95–*t*_8.5 min_ 95–*t*_9.5 min_ 5–*t*_13 min_ 5, flow rate 0.75 mL/min (ibuprofen, fenoprofen, flurbiprofen, **2a**, **2b**); *t*_0 min_ 5–*t*_3 min_ 5–*t*_28 min_ 95–*t*_29 min_ 95–*t*_35 min_ 5–*t*_40 min_ 5, flow rate 0.75 mL/min (**3a**, **3b**, **4a**, **4b**, **5b**, **6b**). Compounds were monitored at wavelengths of 220 and 254 nm. Copies of HPLC chromatograms are given in the Supporting Information (Table S1). Purity was determined at a wavelength of 254 nm.

### 3.5. Evaluation for COX Inhibition

The COX inhibition activity against ovine COX-1 and human recombinant COX-2 was determined using the fluorescence-based COX assay COX Fluorescent Inhibitor Screening Assay Kit (Cayman Chemical Company, Ann Arbor, MI, USA) according to the manufacturer’s instructions as reported [50].

### 3.6. Cyclic Voltammetry Methodology

Cyclic voltammetry (CV) measurements were performed using a Princeton Applied Research computer-controlled potentiostat (PARSTAT 2273 (Berwyn, PA, USA)). For this purpose, a conventional three-electrode cell was employed, equipped with a Pt electrode (A = 0.961 cm^2^) as the working electrode and the reference electrode is Ag/AgNO_3_ (50 mM). Before the voltammetric experiments, the working electrode was polished, rinsed with water, sonicated in water and rinsed successively with water and ethanol, and finally dried under argon gas. The electrochemical measurements were performed in a 0.5 mM solution of each ruthenium complex, dissolved in 0.1 M [*n*-Bu_4_N][PF_6_]/DMSO, which was purged with argon for 30 min before each measurement, in order to remove the oxygen.

### 3.7. Stability Studies Methodology

The stability of compounds **4a**, **5b**, and **6b** was analyzed in sodium ascorbate buffer (150 mM, pH 4.0), PBS (pH 7.4), DMEM (without and with 10% FBS), and RPMI (without and with 10% FBS) using UPLC-MS. The compounds were used to prepare a 10 mM solution in DMSO, and 1 µL of this stock solution was added to the medium (final concentration of 100 µM). After storage at room temperature and protected from light, samples were taken after 24 and 48 h. The samples were diluted with 4 volume parts of cold acetonitrile and in the case of DMEM and RPMI, centrifuged at 4 °C and 12,700 rcf for 5 min. An aliquot of the supernatant was analyzed using the following UPLC-MS system: Waters ACQUITY UPLC I class system including an ACQUITY UPLC PDA e λ detector coupled to a Xevo TQ-S mass spectrometer and equipped with an ACQUITY™ Premier Peptide BEH C18 column (100 mm × 2.1 mm, 1.7 μm, 300 Å) along with an ACQUITY™ Premier Peptide BEH C18 VanGuard Pre-column (5 mm × 2.1 mm, 1.7 μm, 300 Å). A binary linear gradient system of 0.1% CH_3_COOH/H_2_O (A) and 0.1% CH_3_COOH in CH_3_CN/CH_3_OH (1:1, *v*/*v*, B) at a flow rate of 0.4 mL/min and a column temperature of 50 °C served as the eluent. MassLynx (v4.2 SCN986) was used for data processing. Gradient (%B): *t*_0 min_ 45–*t*_0.5 min_ 45–*t*_5.5 min_ 95–*t*_7 min_ 95–*t*_8 min_ 45–*t*_8.5 min_ 45. ESI^+^ mode was used for ionization. A UV–Vis spectrophotometer (ECCU MCS621 UV–Vis CLD600) was used to determine the stability of compounds **4a**, **5b**, and **6b** in the wavelength range 185–715 nm at defined time intervals (0, 24 and 48 h) in sodium ascorbate buffer solutions (pH 4) and PBS (pH 7).

### 3.8. In Vitro Assays

#### 3.8.1. Reagents

RPMI-1640 and DMEM culture medium, fetal bovine serum (FBS), phosphate-buffered saline (PBS), and 100 × Penicillin/Streptomycin mix were purchased from Capricorn Scientific GmbH (Ebsdorfergrund, Germany). Trypsin, dimethyl sulfoxide (DMSO), and crystal violet (CV) were all from Sigma–Aldrich (St. Louis, MO, USA). 3-(4,5-Dimethythiazol-2-yl)-2,5-diphenyltetrazolium bromide (MTT) was purchased from AppliChem (St. Louis, MO, USA). Paraformaldehyde (PFA) was obtained from Serva (Heidelberg, Germany).

Cell culture grade DMSO was used to prepare the stocks of tested compounds (100 mM). The stocks were stored at −20 °C for no longer than a month. Working solutions were made from the stock solutions in the culture media immediately before treatment.

#### 3.8.2. Cell Lines

For the biological part of the study, the following cell lines were used: human melanoma A375, human colorectal adenocarcinoma HT29, human colorectal carcinoma HCT116, human lung carcinoma A549, human mammary carcinoma (MDA-MB-231), and fetal lung fibroblast MRC5. The A375, HCT116, HT29, MDA-MB-231, and MRC5 cell lines were propagated in the HEPES-buffered RPMI-1640 medium, while the A549 cell line was cultivated in the DMEM medium. Both mediums were supplemented with 10% heat-inactivated FBS, 2 mM L-glutamine, 0.01% sodium pyruvate, and antibiotics (penicillin (100 units/mL) and streptomycin (100 μg/mL)). All cells were grown under standard conditions in a humidified atmosphere with 5% CO_2_ at 37 °C.

#### 3.8.3. Viability Assays: MTT and Crystal Violet (CV)

To perform viability tests, cells were seeded in 96-well plates at the following densities: 4 × 10^3^ (A375), 5 × 10^3^ (HCT116), 8 × 10^3^ (HT29), 2 × 10^3^ (A549), 3 × 10^3^ (MDA-MB-231), and 1 × 10^4^ (MRC5) cells/well. All cell lines were treated for 72 h with a range of concentrations of the tested compounds: **3a** and **3b** (3.13–100 µM); **4a**, **4b**, **5b**, **6b** (37.5–200 µM). After treatment, cell viability was assessed using colorimetric MTT and CV assays to evaluate the potential cytotoxicity of the compounds. For the MTT assay, cells were incubated with MTT solution in cell culture medium (0.5 mg/mL) for 30–90 min at 37 °C. The resulting formazan crystals were dissolved in DMSO. For the CV assay, the cells were first fixed with 4% PFA for 10 min at room temperature (RT), and then stained with a 0.02% CV solution for 15 min, also at RT. After staining, the cells were washed with tap water, air-dried, and the bound dye was dissolved in 33% acetic acid. For both assays, absorbance was measured using an automated microplate reader at 540/670 nm. Cell viability was expressed as a percentage relative to untreated control cells, which were arbitrarily set at 100%. IC_50_ values were calculated using a four-parameter logistic function and are presented as mean ± standard deviation (SD). All experiments were performed in triplicate.

#### 3.8.4. Statistical Analysis

The obtained data were expressed as mean ± SD based on at least three independent experiments. Statistical comparisons were performed using Student’s *t*-test, and differences between groups were considered significant at *p* < 0.05.

## 4. Conclusions

A series of ruthenacarborane-(*η*^6^*-p*-cymene)–NSAID conjugates (**4a**, **4b**, **5b**, and **6b**) were successfully synthesized using methylene or ethylene spacers and ester linkers to attach NSAIDs (ibuprofen, flurbiprofen, and fenoprofen) to carborane. These conjugates were fully characterized by multinuclear NMR spectroscopy (^1^H, ^11^B, and ^13^C), confirming their structural integrity. Biological evaluation revealed that the *nido*-carborane precursors (**2a**, **2b**) exhibit a higher inhibition for COX-1 and COX-2 than their *closo*-ruthenacarborane analogues (**3a**, **3b**), with a slight preference for COX-2, while the potential to reduce cell viability was better for **3a** and **3b**. Among both series, the derivatives with a longer ethylene spacer (**2b**, **3b**) showed greater inhibition for COX-2 compared to those with a shorter methylene spacer (**2a**, **3a**), suggesting the importance of linker length in modulating activity. Upon final conjugation with NSAIDs, a slight increase in inhibitory activity was observed; however, this enhancement was not substantial. These conjugates also displayed higher selectivity toward COX-1 over COX-2, indicating limited therapeutic selectivity for inflammation or cancer-related targets. Comparative analysis with the corresponding free NSAID ligands demonstrated that *nido*-carborane–NSAID conjugates (**10a**, **10b**, **11b**, and **12b** [37]) were unexpectedly more active than the ruthenacarborane-(*η*^6^*-p*-cymene)–NSAID conjugates and, importantly, showed higher selectivity toward COX-2 than COX-1. The *closo*-ruthenacarborane complexes (**3a** and **3b**) exhibited significant cytotoxicity, with similar IC_50_ values across multiple cancer cell lines. However, their selectivity for malignant cells over normal cells was moderate and did not reach statistical significance, even in the most responsive cancer cell lines. In contrast, the ruthenacarborane-(*η*^6^*-p*-cymene)–NSAID conjugates displayed no measurable cytotoxic effects across the tested cancer cell lines. Electrochemical studies using CV revealed that Ru^II^ undergoes oxidation to Ru^III^, shows unexpected redox behavior, which explains the reduced or complete loss of the pharmacological activity of the conjugated drugs. Notably, compound **6b** showed similar degradation patterns at both pH 4 and pH 7, as confirmed by UV–Vis studies, indicating non-selective hydrolysis, while UPLC-MS studies showed no hydrolysis of the conjugates. In contrast to conjugates reported by Gozzi et al. [24], the chiral part of the NSAIDs in the conjugates studied in this work may reduce target affinity, interfere with cellular uptake, or hinder efficient intracellular release, which emphasizes that not only the choice of pharmacophore, but also the nature and length of the connecting spacer are crucial design parameters in the development of ruthenacarborane-based hybrid drugs. These findings suggest that conjugation of ruthenacarborane with NSAIDs does not necessarily enhance their anticancer potential.

## Figures and Tables

**Table 1:** Selected CV parameters (at 100 mV/s) for the compounds **4a**, **5b** and **6b**.

		4a	5b	6b
Ru center	E_pc_ [V vs. Ag/Ag^+^]	−1.42	−1.316	−1.216
	E_pa_ [V vs. Ag/Ag^+^]	−0.941	−0.918	−0.93
	E_1/2_ [V vs. Ag/Ag^+^]	−1.18	−1.12	−1.07
	ΔE_p_ [V]	0.479	0.398	0.286
	k° [cm/s]	8.4×10^−6^	1.2×10^−5^	5.2×10^−5^
Ligand	E_pc_ [V vs. Ag/Ag^+^]	−2.43	−2.21	-
	E_pa_ [V vs. Ag/Ag^+^]	−1.91	-	-

**Table 2:** COX inhibitory potential of *nido*-carborane (**2a**, **2b**), ruthenacarborane (**3a**, **3b**) and ruthenacarborane-(*η*^6^*-p*-cymene)–NSAID conjugates (**4a**, **4b**, **5b**, and **6b**) compared with their respective *closo*-carborane and *nido*-carborane conjugates to celecoxib and SC-560.

	% Inhibition @ 100 µM		IC_50_ [µM]		SI ***
	COX-1	COX-2	COX-1	COX-2	
**1a**	n.i. *	n.i.	- **	-	-
**1b**	n.i.	n.i.	-	-	-
**2a**	6	38	-	-	-
**2b**	23	56	-	84.1	>1.2
**3a**	18	14	-	-	-
**3b**	21	25	-	-	-
**4a**	33	18	-	-	-
**4b**	40	21	-	-	-
**5b**	n.i.	n.i.	-	-	-
**6b**	30	11	-	-	-
**7a** [36]	63	37	30.6	>100	<0.3
**7b** [36]	n.i.	23	-	-	-
**8b** [36]	n.i.	11	-	-	-
**9b** [36]	15	n.i.	-	-	-
**10a** [37]	105	104	1.645	7.845	0.21
**10b** [37]	106	104	3.132	4.509	0.69
**11b** [37]	104	105	7.173	4.004	1.8
**12b** [37]	104	103	10.75	2.879	3.7
**Ibuprofen**	n.i.	27	-	-	-
**Flurbiprofen**	82	97	0.85	0.07	11.2
**Fenoprofen**	5	26	-	-	-
**Celecoxib**	-	-	-	0.138	-
**SC-560**	-	-	0.013	-	-

* n.i. = no inhibition (%inhibition below 5%); ** - = not determined; Celecoxib served as reference for COX-2: pIC_50_(pIC_50_ = −log_10_(IC_50_[M])); SC-560 served as reference for COX-1; *** SI = Selectivity Index, SI = IC_50_ (COX-1)/IC_50_ (COX-2); literature values for compound **7a**–**12b** [36,37].

**Table 3:** IC_50_ [μM] values ± standard deviations of *nido*-carborane (**2a**, **2b**), ruthenacarborane (**3a**, **3b**) and ruthenacarborane-(*η*^6^-*p*-cymene)–NSAID conjugates (**4a**, **4b**, **5b**, and **6b**) determined by MTT and CV viability assays after 72 h of treatment.

	A375		A549		HCT116		HT29		MDA-MB-231		MRC5	
	MTT	CV	MTT	CV	MTT	CV	MTT	CV	MTT	CV	MTT	CV
**2a**	>200	>200	>200	191.7 ± 11.8	>200	>200	>200	>200	>200	>200	>200	>200
**2b**	>200	>200	>200	176.1 ± 1.1	>200	>200	>200	>200	>200	187.7 ± 5.9	>200	>200
**3a**	20.5 ± 1.8	25.1 ± 2.6	20.6 ± 1.3	21.4 ± 1.1	22.8 ± 1.6	23.4 ± 0.7	33.4 ± 2.8	25.8 ± 2.2	31.6 ± 1.9	26.8 ± 1.6	35.4 ± 1.2	34.9 ± 2.1
**3b**	20.9 ± 2.0	29.0 ± 2.2	19.7 ± 0.1	19.5 ± 1.8	19.9 ± 0.4	21.8 ± 1.2	36.3 ± 0.8	33.4 ± 0.8	23.3 ± 0.8	18.6 ± 1.3	35.0 ± 1.8	30.0 ± 2.9
**4a**	>200	>200	>200	>200	>200	>200	>200	>200	>200	>200	>200	>200
**4b**	>200	>200	>200	>200	>200	>200	>200	>200	>200	>200	>200	>200
**5b**	>200	>200	>200	>200	>200	>200	>200	>200	>200	>200	>200	>200
**6b**	>200	>200	>200	>200	>200	>200	>200	>200	>200	>200	>200	>200

## Data Availability

The supporting data report can be found in the Supplementary Materials.

## Supplementary Materials

The following supporting information can be downloaded at: https://www.mdpi.com/article/10.3390/molecules30214153/s1 and includes ^1^H, ^13^C{^1^H} and ^11^B{^1^H} NMR spectra, analysis of purity by High Performance Liquid Chromatography (HPLC), and viability curves of **3a**, **3b**, **4a**, **4b**, **5b**, and **6b**. Scheme S1: Attempted synthesis of ruthenium(II) complexes with pre-conjugated NSAIDs and hydroxyalkyl-*ortho*-carborane; Scheme S2: Attempted synthesis of ruthenium(II) complexes with carborane-hydroxyalkyl ligands; Figure S1: ^1^H NMR spectrum of **2a** in acetone-d_6_; Figure S2: ^11^B{^1^H} NMR spectrum of **2a** in acetone-d_6_; Figure S3: ^13^C{^1^H} NMR spectrum of **2a** in acetone-d_6_; Figure S4: ^1^H NMR spectrum of **2b** in acetone-d_6_; Figure S5: ^11^B{1H} NMR spectrum of **2b** in acetone-d_6_; Figure S6: ^13^C{^1^H} NMR spectrum of **2b** in acetone-d_6_; Figure S7: ^1^H NMR spectrum of **3a** in CDCl_3_; Figure S8: ^11^B{^1^H} NMR spectrum of **3a** in CDCl_3_; Figure S9: ^13^C{^1^H} NMR spectrum of **3a** in CDCl_3_; Figure S10: ^1^H NMR spectrum of **3b** in CDCl_3_; Figure S11: ^11^B{^1^H} NMR spectrum of **3b** in CDCl_3_; Figure S12: ^13^C{^1^H} NMR spectrum of **3b** in CDCl_3_; Figure S13: ^1^H NMR spectrum of **4a** in CDCl_3_; Figure S14: ^11^B{^1^H} NMR spectrum of **4a** in CDCl_3_; Figure S15: ^13^C{^1^H} NMR spectrum of **4a** in CDCl_3_; Figure S16: ^1^H NMR spectrum of **4b** in CDCl_3_; Figure S17: ^11^B{^1^H} NMR spectrum of **4b** in CDCl_3_; Figure S18: ^13^C{^1^H} NMR spectrum of **4b** in CDCl_3_; Figure S19: ^1^H NMR spectrum of **5b** in CDCl_3_; Figure S20: ^11^B{^1^H} NMR spectrum of **5b** in CDCl_3_; Figure S21: ^13^C{^1^H} NMR spectrum of **5b** in CDCl_3_; Figure S22: ^1^H NMR spectrum of **6b** in CDCl_3_; Figure S23: ^11^B{^1^H} NMR spectrum of **6b** in CDCl_3_; Figure S24: ^13^C{^1^H} NMR spectrum of **6b** in CDCl_3_; Figure S25: Comparison between ruthenium complexes **3a** and **4a**; Figure S26: Stability of compound **4a** over 72 h in DMSO (^1^H NMR spectroscopy); Figure S27: UV/Vis spectra of **4a**, **5b**, and **6b** at pH 4 and 7 at 0, 24 and 48 h; Figure S28: COX-2 inhibition data of **2b**; Figure S29: Count of particles of compound **4a**, **4b**, **5b**, and **6b** STD (200 nm) and solvent (DMSO + PBS); Figure S30: Comparison of CVs obtained for **4a**, **5b**, and **6b**; Figure S31: Cyclic voltammograms at different current of **4a**, **5b**, and **6b**; Figure S32: The effect of **2a** and **2b** on the viability of human cancer cell lines and primary transformed lung embryonal fibroblasts; Figure S33: The effect of **3a** and **3b** on the viability of human cancer cell lines and primary transformed lung embryonal fibroblasts; Figure S34: The effect of **4a**, **4b**, **5b**, and **6b** on the viability of human cancer cell lines and primary transformed lung embryonal fibroblasts; Table S1: Results of HPLC analyses of ibuprofen, fenoprofen, flurbiprofen, **2a**, **2b**, **3a**, **3b**, **4a**, **4b**, **5b**, and **6b** at 220 and 254 nm; Table S2: Total number concentration and particles concentration peak of compound **4a**, **4b**, **5b,** and **6b** particles (200 nm) and solvent (DMSO + PBS): Table S3: Stability test by UPLC-MS. Compounds, retention times and m/z.

## Abbreviations

The following abbreviations are used in this manuscript:

NSAIDs	Non-Steroidal Anti-Inflammatory Drugs
COX	Cyclooxygenase
NMR	Nuclear Magnetic Resonance
CV	Crystal Violet
CV	Cyclic Voltammetry
MTT	3-(4,5-Dimethylthiazol-2-yl)-2,5-diphenyltetrazolium bromide
HPLC	High-Performance Liquid Chromatography
HATU	*O*-(7-Azabenzotriazol-1-yl)-*N*,*N*,*N′*,*N′*-tetramethyluronium hexafluorophosphate
DIPEA	*N*,*N*-Diisopropylethylamine
DLS	Dynamic Light Scattering
PBS	Phosphate-Buffered Saline
PFA	Paraformaldehyde
TMS	Tetramethylsilane
BNCT	Boron Neutron Capture Therapy
TLC	Thin-Layer Chromatography
