# Supplementary material for: Novel Ruthenacarborane–NSAID Conjugates
Source: Molecules. 2025 Oct 22;30(21):4153. doi: 10.3390/molecules30214153 (PMC12608622; doi:10.3390/molecules30214153)
Supplement: Supplementary file 1 [file molecules-30-04153-s001.zip › molecules-3904222-supplementary.pdf]

Contents

1. Failed Attempts S4

Scheme S1: Attempted synthesis of ruthenacarborane-(η^6-*p*-cymene)−NSAID conjugates from *nido*-carborate–NSAID conjugates. S4

Scheme S2: Attempted synthesis of ruthenacarborane alcohols S4

2. Cesium 1-(1'-Hydroxymethyl)-*nido*-carborane (**2a**): S5

Figure S1. ^{1}H NMR spectrum of **2a** in acetone-d_6. .... S5

Figure S2. ^{11}B{^{1}H} NMR spectrum of **2a** in acetone-d_6 .... S5

Figure S3. ^{13}C{^{1}H} NMR spectrum of **2a** in acetone-d_6 .... S6

3. Cesium 1-(2'-Hydroxyethyl)-*nido*-carborane (**2b**) S7

Figure S4. ^{1}H NMR spectrum of **2b** in acetone-d_6. .... S7

Figure S5. ^{11}B{^{1}H} NMR spectrum of **2b** in acetone-d_6. .... S7

Figure S6. ^{13}C{^{1}H} NMR spectrum of **2b** in acetone-d_6. .... S8

4. [Ru(η^6-*p*-cymene)-1-(1′-hydroxymethyl)-*closo*-$C_2B_9H_{10}$] (**3a**) S8

Figure S7. ^{1}H NMR spectrum of **3a** in $CDCl_3$. S9

Figure S8. ^{11}B{^{1}H} NMR spectrum of **3a** in $CDCl_3$. S9

Figure S9. ^{13}C{^{1}H} NMR spectrum of **3a** in $CDCl_3$. S10

5. [Ru(η^6-*p*-cymene){1-(2′-hydroxyethyl)-*closo*-$C_2B_9H_{10}$}] (**3b**) S10

Figure S10. ^{1}H NMR spectrum of **3b** in $CDCl_3$. S11

Figure S11. ^{11}B{^{1}H} NMR spectrum of **3b** in $CDCl_3$. S11

Figure S12. ^{13}C{^{1}H} NMR spectrum of **3b** in $CDCl_3$ S12

6. Ruthenacarborane-(*p*-cymene)–NSAID Conjugates S13

[Ru(η^6-*p*-cymene)-1-{methyl (*R/S*)-2-[4-(2-fluorobiphenyl-4-yl)propanoic acid ester]-*closo*-$C_2B_9H_{10}$}] (**4a**) S13

Figure S13. ^{1}H NMR spectrum of **4a** in $CDCl_3$. S13

Figure S14. $^{11}B\{^1H\}$ NMR spectrum of **4a** in $CDCl_3$. S13

Figure S15. $^{13}C\{^1H\}$ NMR spectrum of **4a** in $CDCl_3$ S14

[Ru(η^6-*p*-cymene)-2-{ethyl (*R/S*)-2-[4-(2-fluorobiphenyl-4-yl)propanoic acid ester]-*closo*-$C_2B_9H_{10}$}] (**4b**) S14

Figure S16. ^{1}H NMR spectrum of **4b** in $CDCl_3$. S14

Figure S17. $^{11}B\{^1H\}$ NMR spectrum of **4b** in $CDCl_3$. S15

Figure S18. $^{13}C\{^1H\}$ NMR spectrum of **4b** in $CDCl_3$. S15

[Ru(η^6-*p*-cymene)-2-[ethyl-2-(3-phenoxyphenyl)propanoic ester]-*closo*-$C_2B_9H_{10}$] (**5b**): S16

Figure S19. ^{1}H NMR spectrum of **5b** in $CDCl_3$. S16

Figure S20. $^{11}B\{^1H\}$ NMR spectrum of **5b** in $CDCl_3$. S16

Figure S21. $^{13}C\{^1H\}$ NMR spectrum of **5b** in $CDCl_3$. S17

[Ru(η^6-*p*-cymene)-2-{ethyl (*R/S*)-2-[4-(2-propylphenyl)propanoic acid ester]-*closo*-$C_2B_9H_{10}$}] (**6b**) S17

Figure S22. ^{1}H NMR spectrum of **6b** in $CDCl_3$. S17

Figure S23. $^{11}B\{^1H\}$ NMR spectrum of **6b** in $CDCl_3$. S18

Figure S24. $^{13}C\{^1H\}$ NMR spectrum of **6b** in $CDCl_3$. S18

7. Comparison between ruthenacarborane (**3a**) and ruthenacarborane-(η^6-*p*-cymene)−NSAID conjugates with flurbiprofen (**4a**) S19

Figure S25. Comparison between ruthenium complexes **3a** and **4a**. S19

8. Stability Test S20

Figure S26. Stability of compound **4a** over 72 h in DMSO (^{1}H NMR spectroscopy). S20

9. UV-VIS Spectroscopy S20

Figure S27. UV/Vis spectra of **4a**, **5b** and **6b** at pH 4 and 7 at 0, 24 and 48 h. S20

10. Analysis of Purity by High-Performance Liquid Chromatography (HPLC) S21

Table S1. Results of HPLC analyses of ibuprofen, fenoprofen, flurbiprofen, **2a**, **2b**, **3a**, **3b**, **4a**, **4b**, **5b** and **6b** at 220 and 254 nm. S21

11. Evaluation for COX Inhibition S23

Figure S28. COX-2 inhibition data of **2b**. S23

12. Dynamic Light Scattering (DLS) Measurements S23

Figure S29: Count of particles of compound **4a**, **4b**, **5b** and **6b** STD (200 nm) and solvent (DMSO + PBS). S23

Table S2. Total number concentration and particles concentration peak of compound **4a**, **4b**, **5b** and **6b** particles (200 nm) and solvent (DMSO + PBS). S24

13. Cyclic Voltammetry (CV) S24

Figure S30. Comparison of CVs obtained for **4a**, **5b**, and **6b**. S24

Figure S31. Cyclic voltammograms at different current of **4a**, **5b**, and **6b**. S25
14. Cell Viability S26
Figure S32. The effect of **2a** and **2b** on the viability of human cancer cell lines and primary transformed lung embryonal fibroblasts... S26
Figure S33. The effect of **3a** and **3b** on the viability of human cancer cell lines and primary transformed lung embryonal fibroblasts... S27
Figure S34. The effect of **4a**, **4b**, **5b**, and **6b** on the viability of human cancer cell lines and primary transformed lung embryonal fibroblasts S28
15. Stability Test by UPLC-MS S29
Table S3: Stability test by UPLC-MS. Compounds, retention times and m/z. S29
Figure S35: Stability studies of compound **4a** in various media over 48 h. S30
Figure S36: Stability studies of compound **5b** in various media over 48 h. S31
Figure S37: Stability studies of compound **6b** in various media over 48 h. S32

1. Failed Attempts

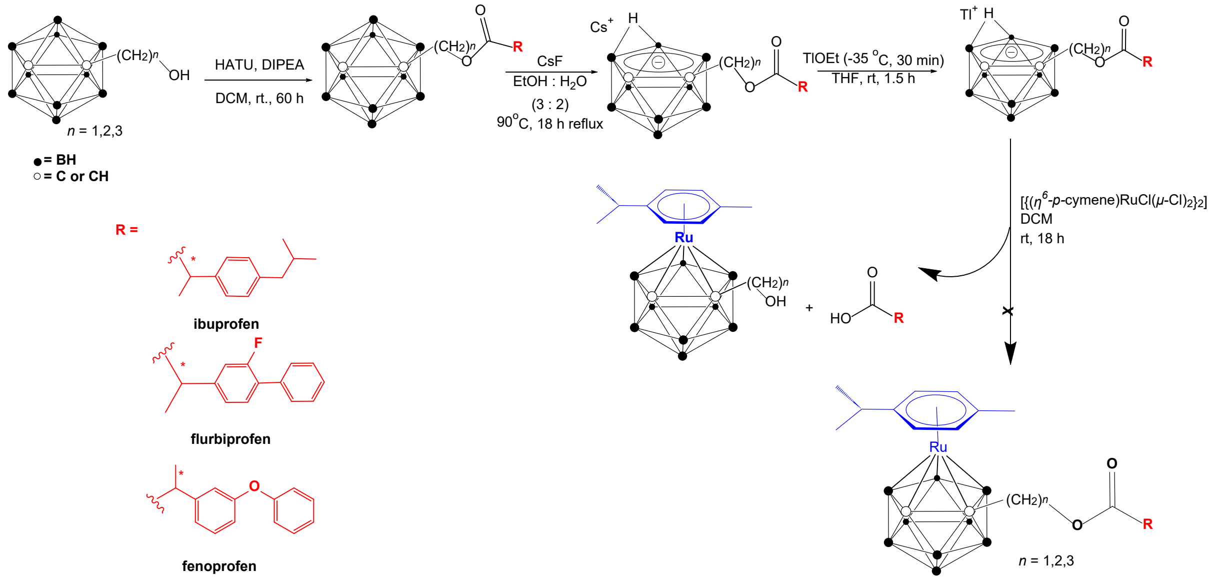

Scheme S1: Attempted synthesis of ruthenacarborane-(η^6-*p*-cymene)–NSAID conjugates from *nido*-carborate–NSAID conjugates.

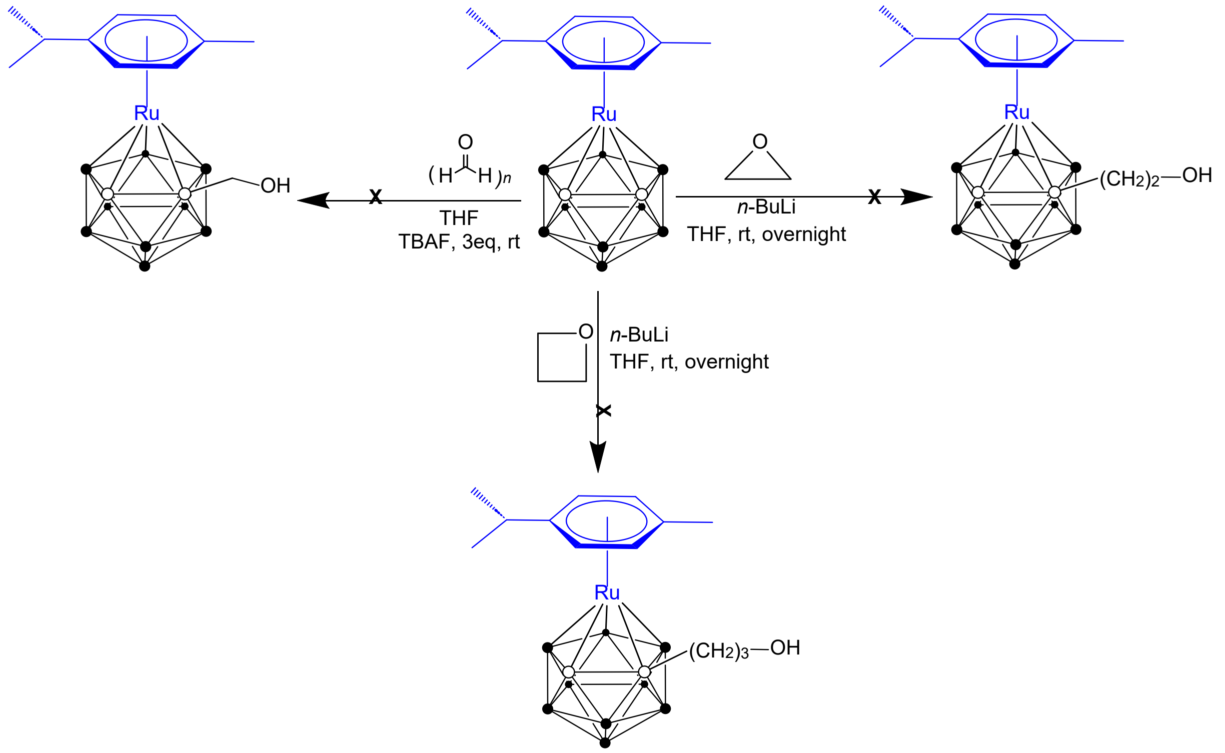

Scheme S2: Attempted synthesis of ruthenacarborane alcohols.

2. Cesium 1-(1’-Hydroxymethyl)-*nido*-carborane (**2a**)

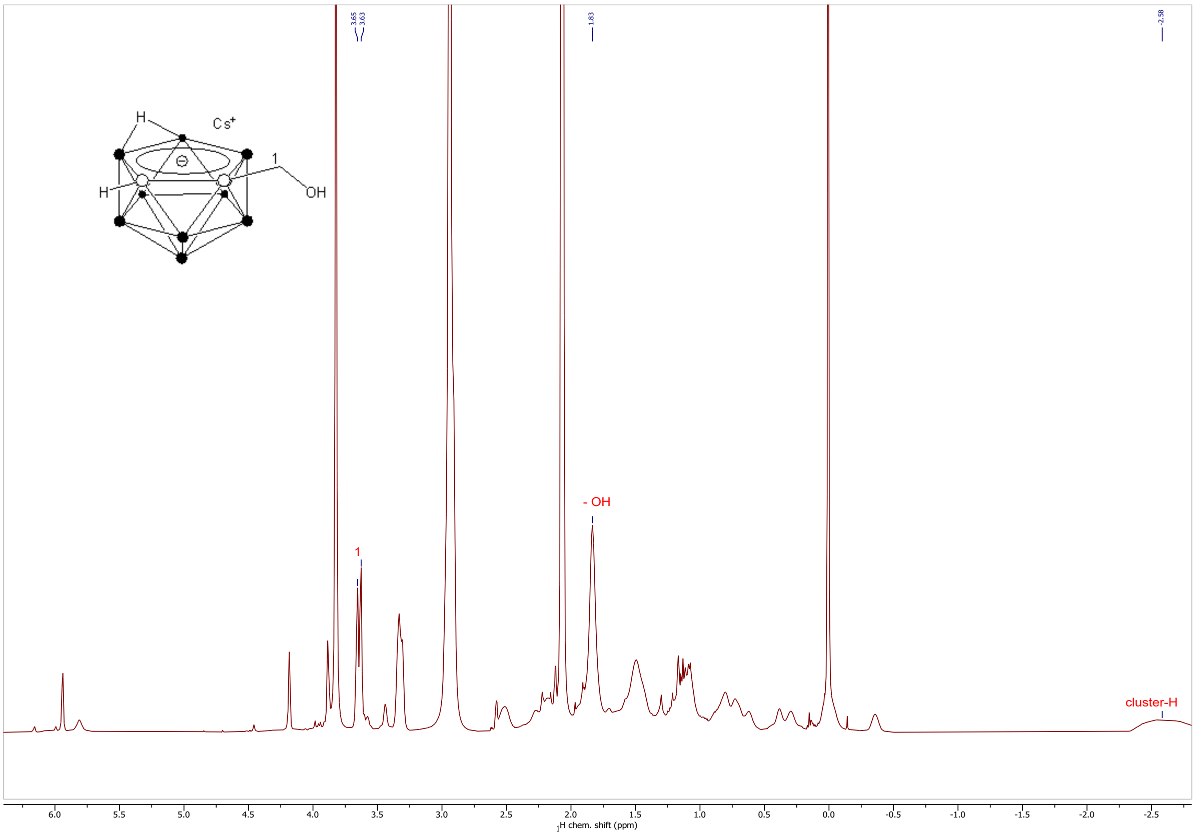

Figure S1. ^{1}H NMR spectrum of **2a** in acetone-d$_6$.

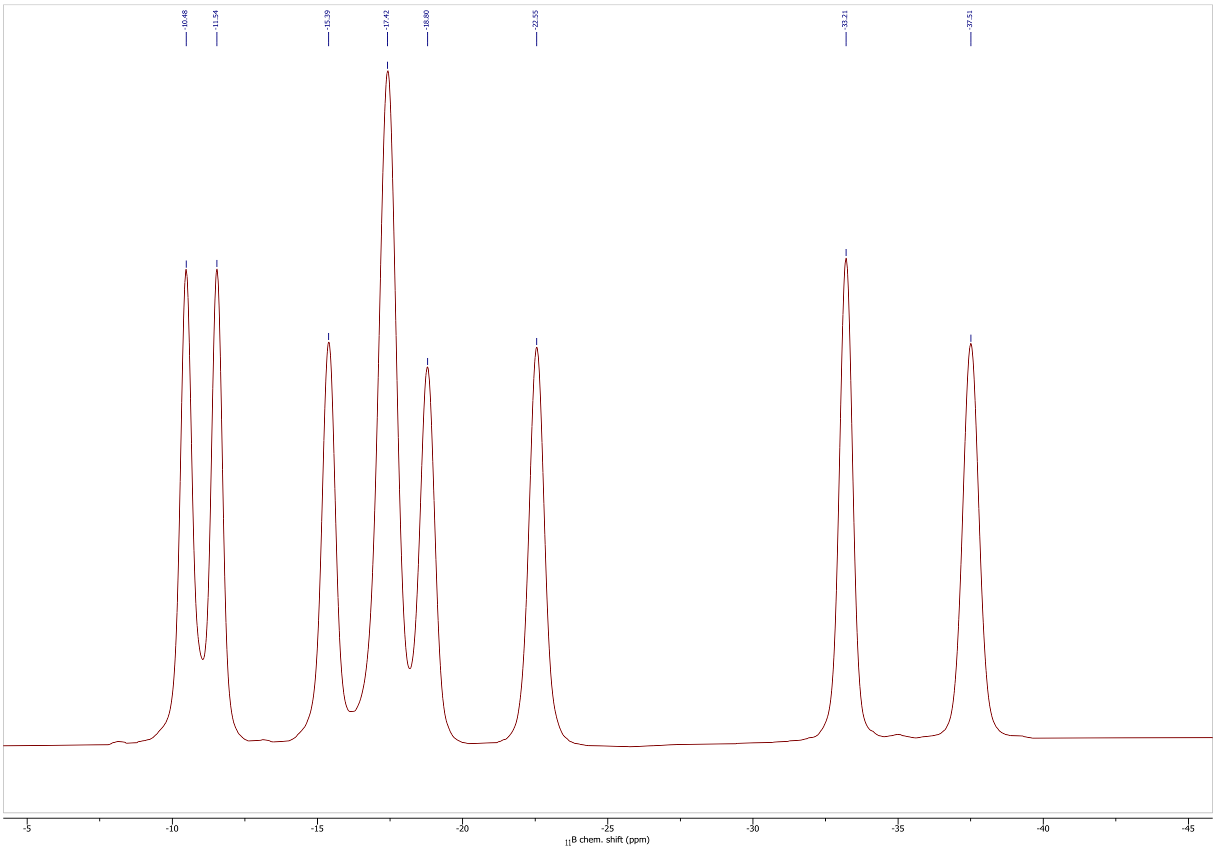

Figure S2. ^{11}B{^{1}H} NMR spectrum of **2a** in acetone-d$_6$.

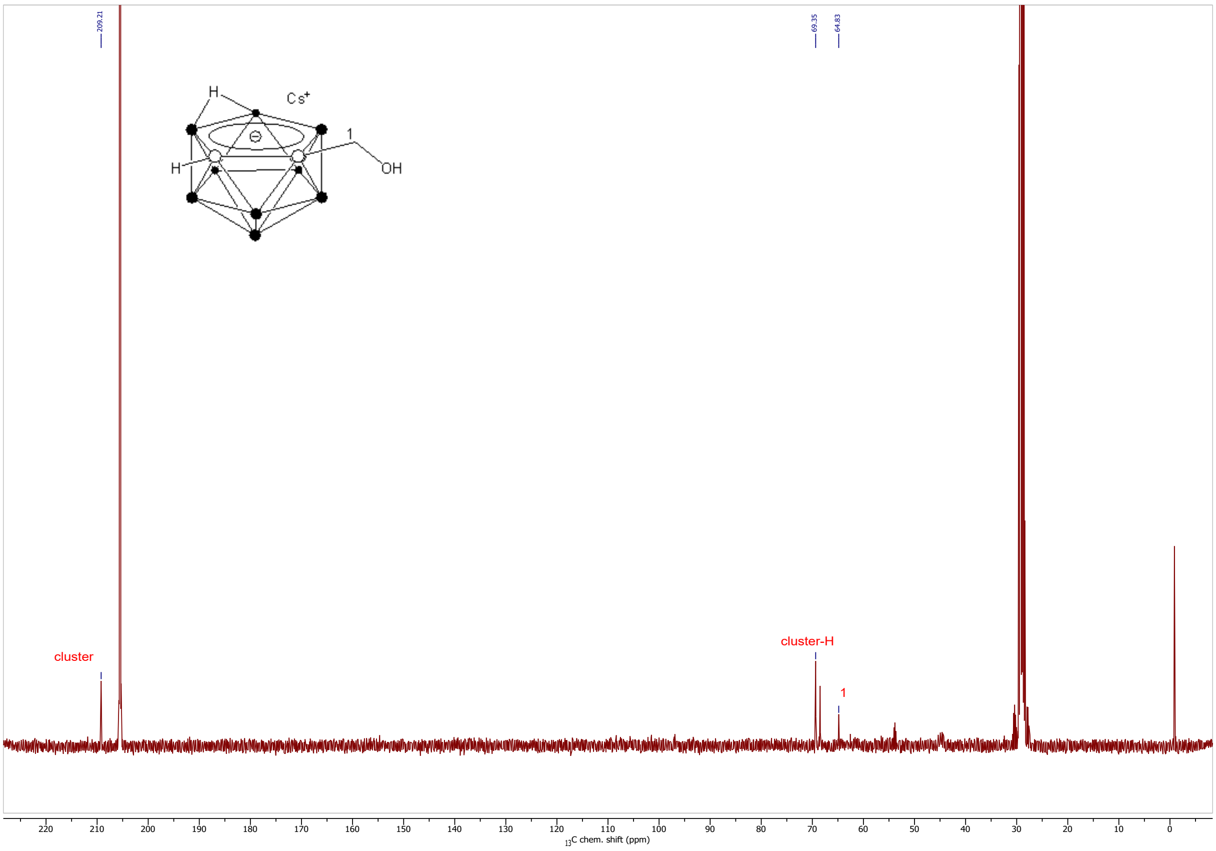

Figure S3. $^{13}C\{^{1}H\}$ NMR spectrum of **2a** in acetone-d_6.

3. Cesium 1-(2'-Hydroxyethyl)-*nido*-carborane (**2b**)

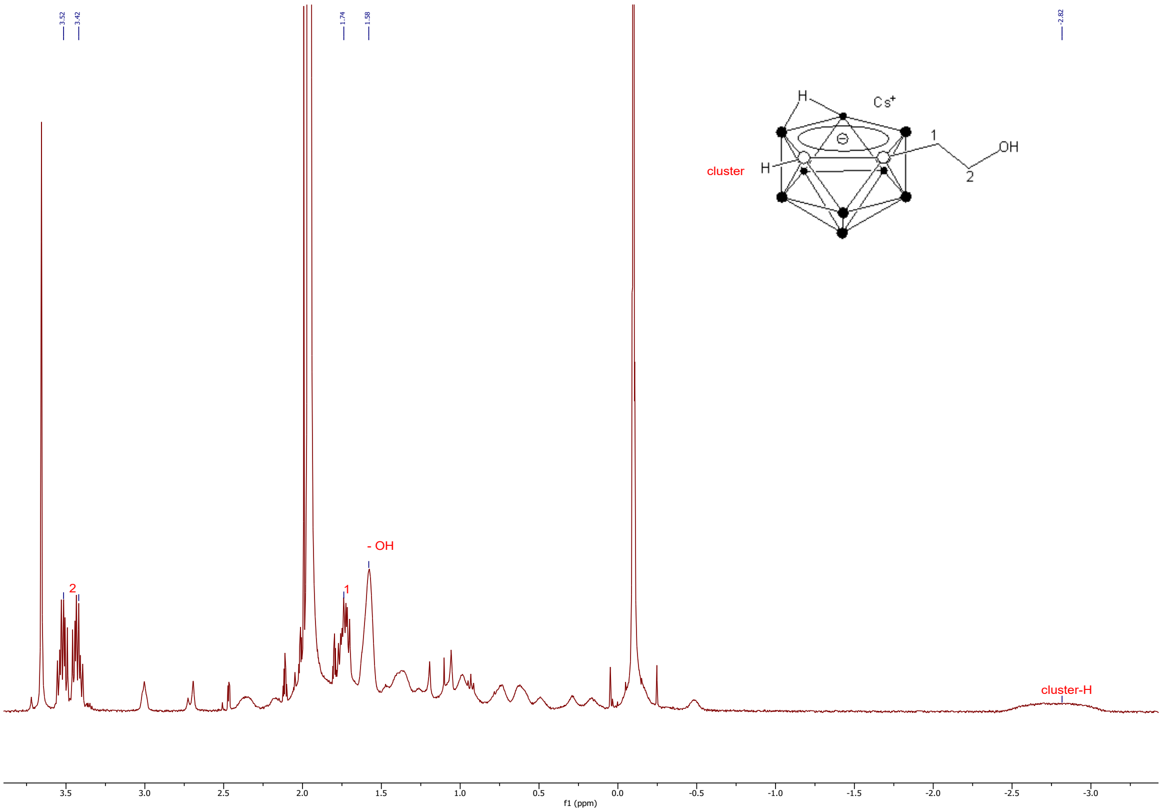

Figure S4. ^{1}H NMR spectrum of **2b** in acetone-d_6.

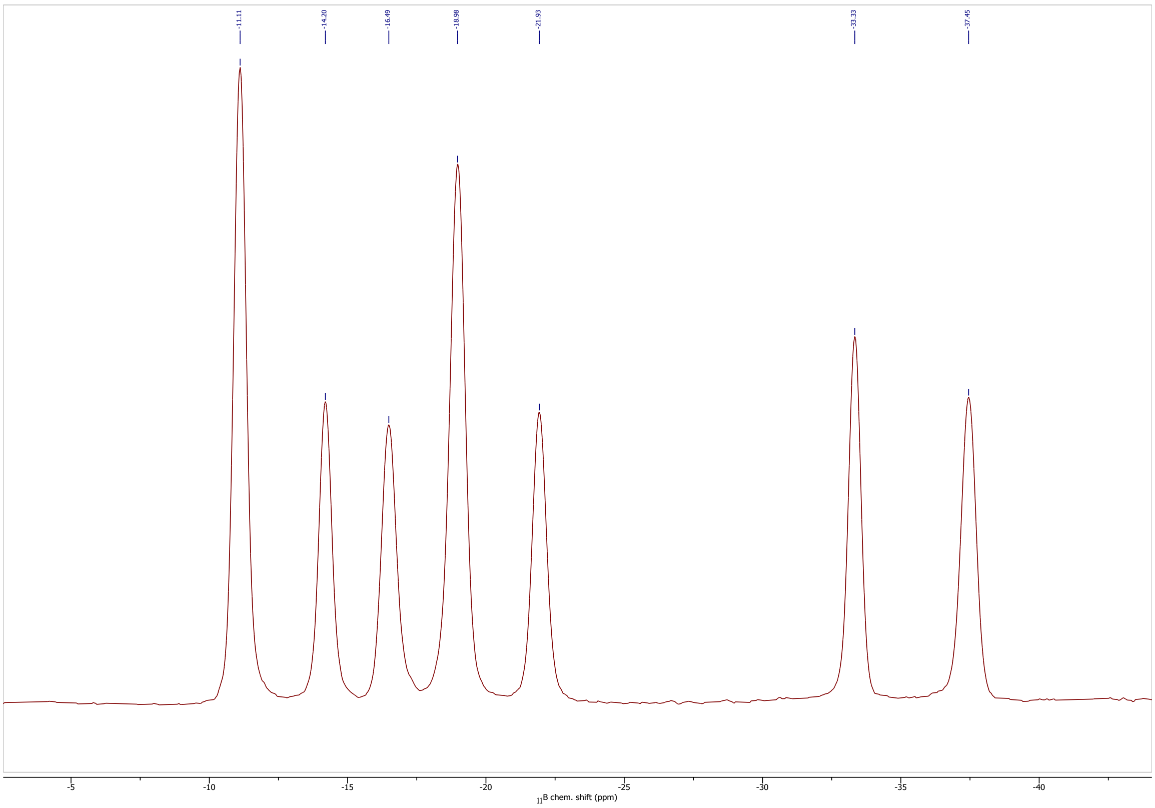

Figure S5. $^{11}B\{^{1}H\}$ NMR spectrum of **2b** in acetone-d_6.

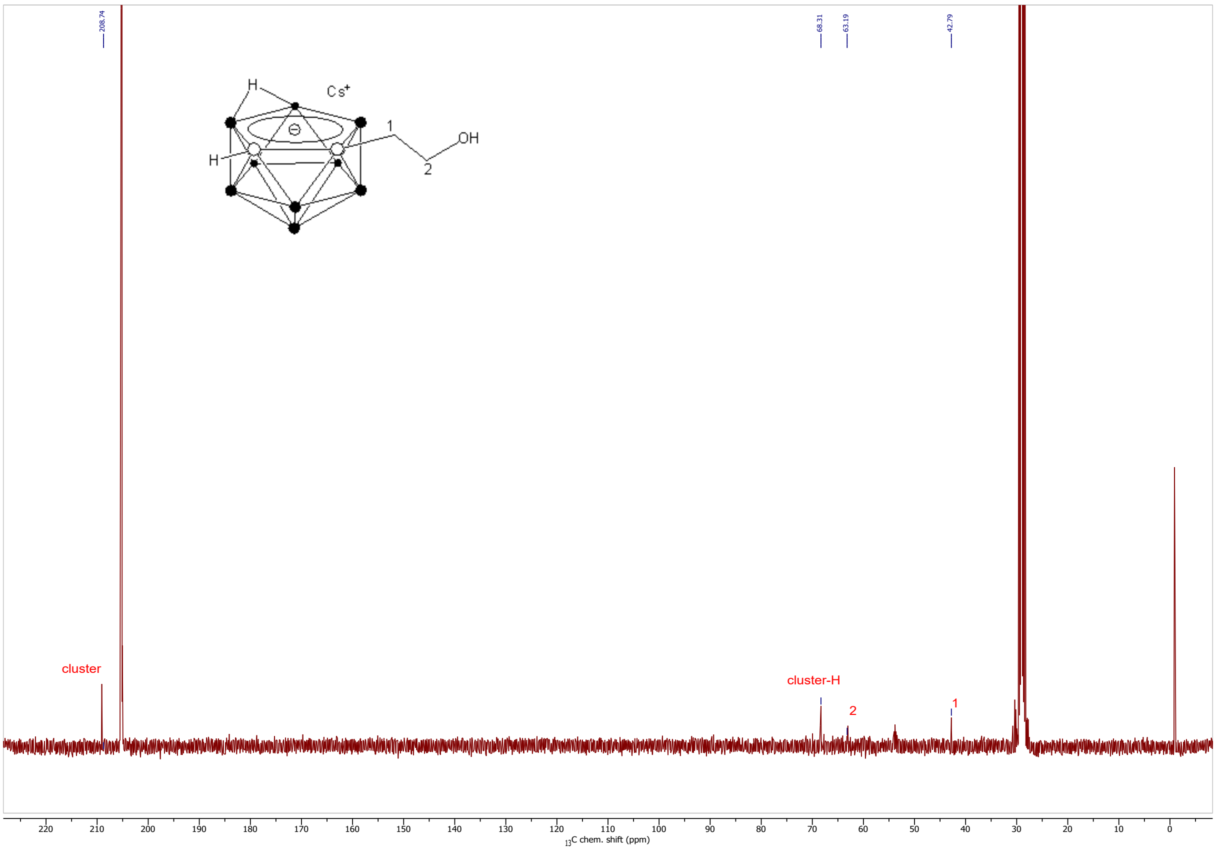

Figure S6. $^{13}C\{^{1}H\}$ NMR spectrum of **2b** in acetone-d$_6$.

4. [Ru(η^6-*p*-cymene){1-(1´-hydroxymethyl)-*closo*-$C_2B_9H_{10}$}] (**3a**):

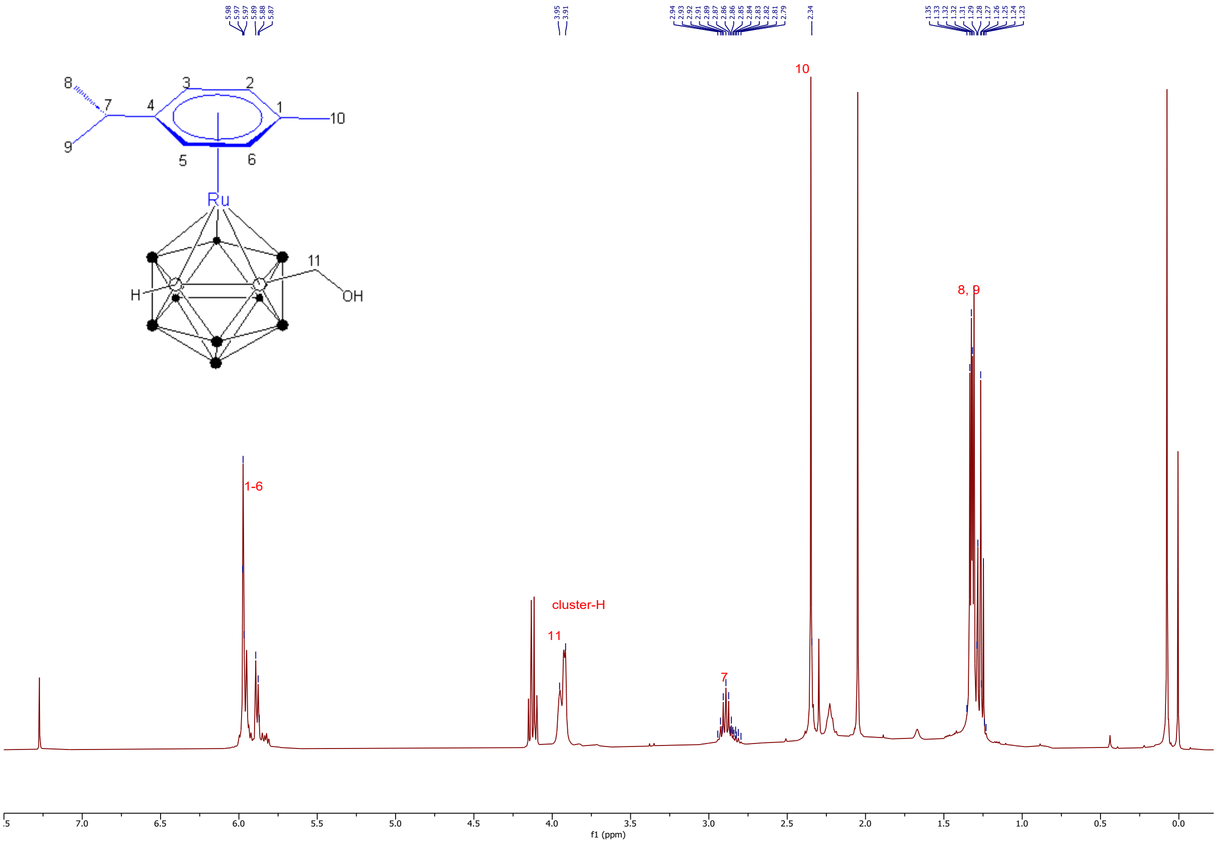

Figure S7. ^{1}H NMR spectrum of **3a** in $CDCl_3$.

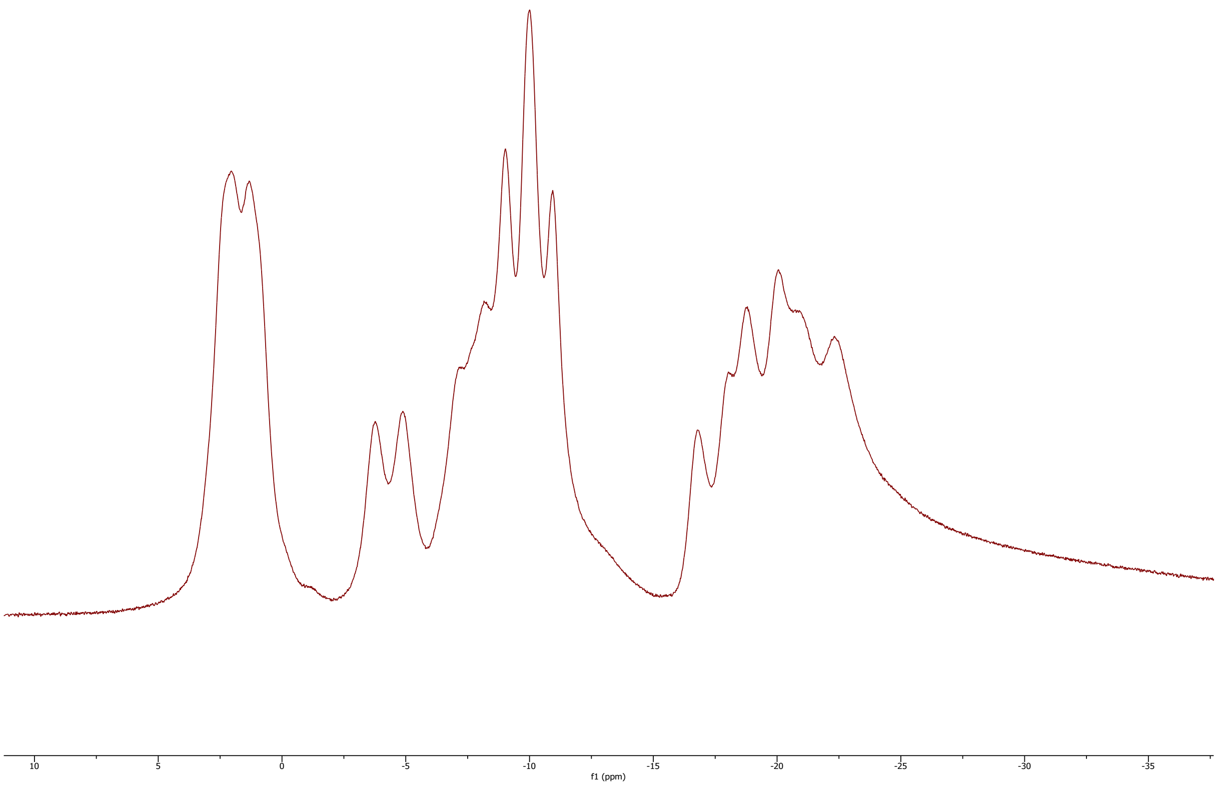

Figure S8. $^{11}B\{^{1}H\}$ NMR spectrum of **3a** in $CDCl_3$.

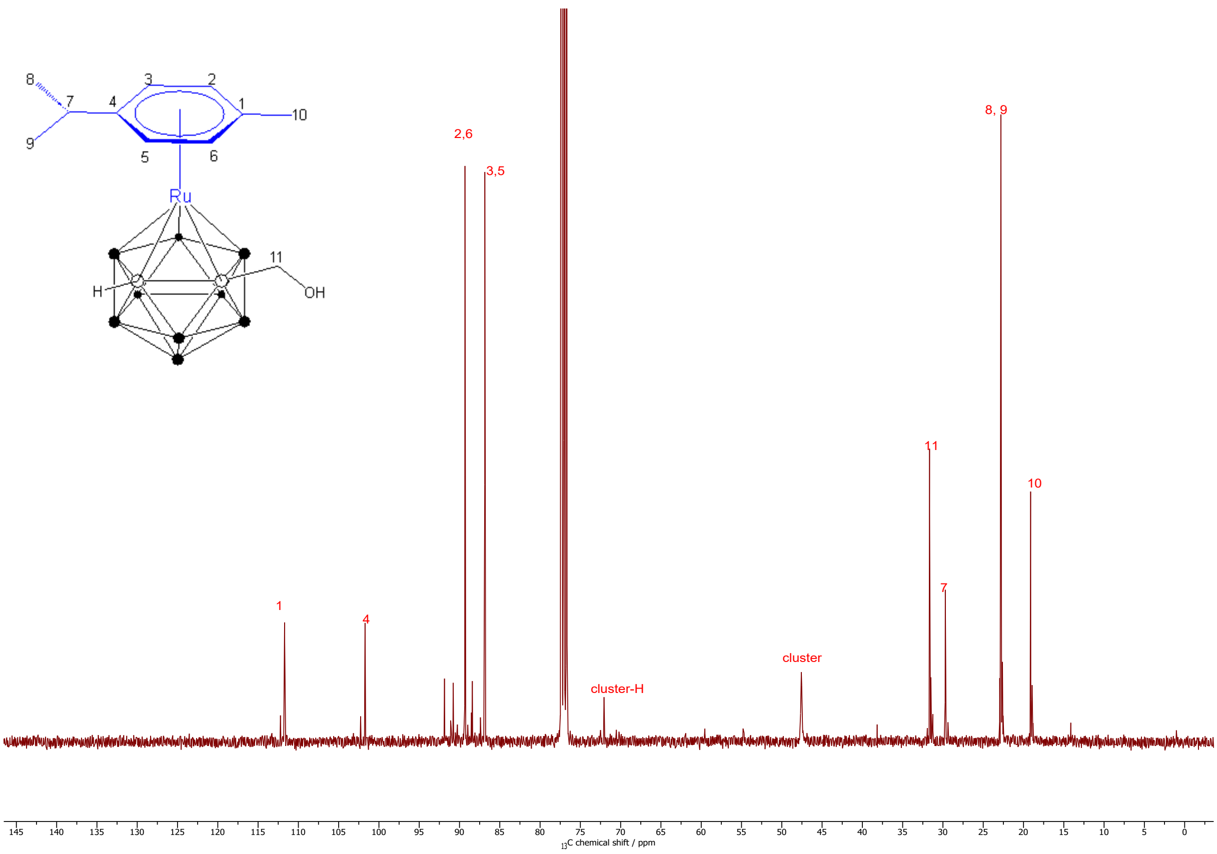

Figure S9. $^{13}C\{^{1}H\}$ NMR spectrum of **3a** in $CDCl_3$.

5. [Ru(η^6-*p*-cymene){1-(2´-hydroxyethyl)-*closo*-$C_2B_9H_{10}$}] (**3b**)

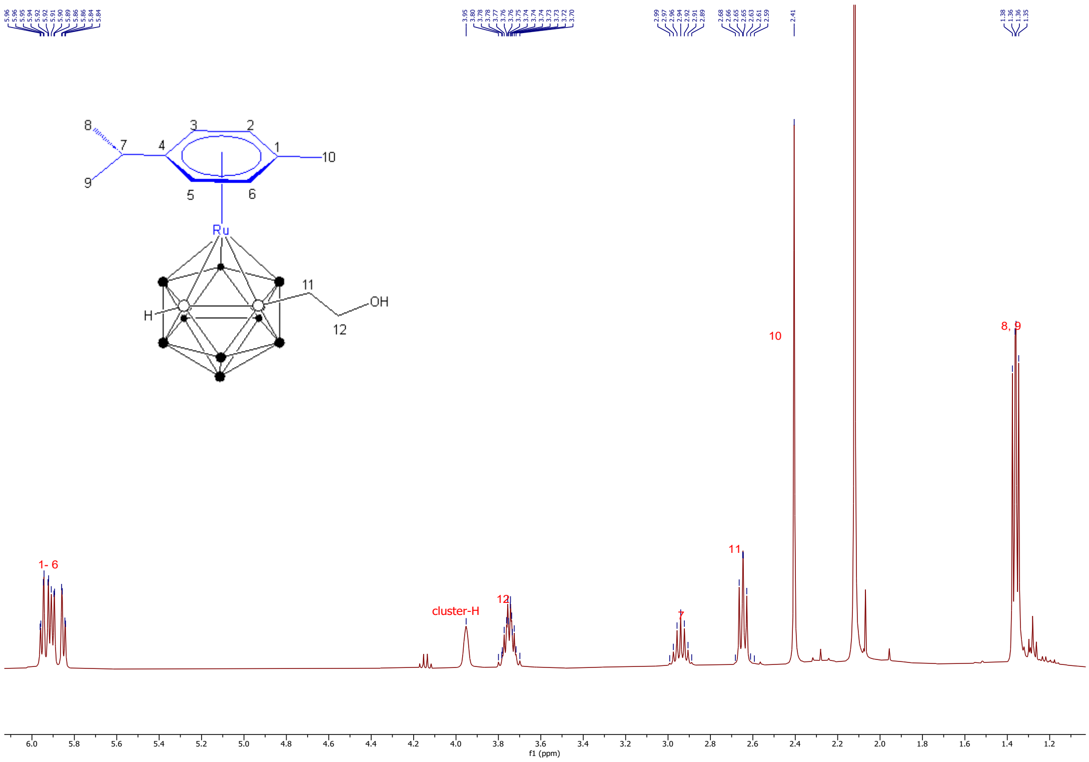

Figure S10. ^1H NMR spectrum of **3b** in $CDCl_3$.

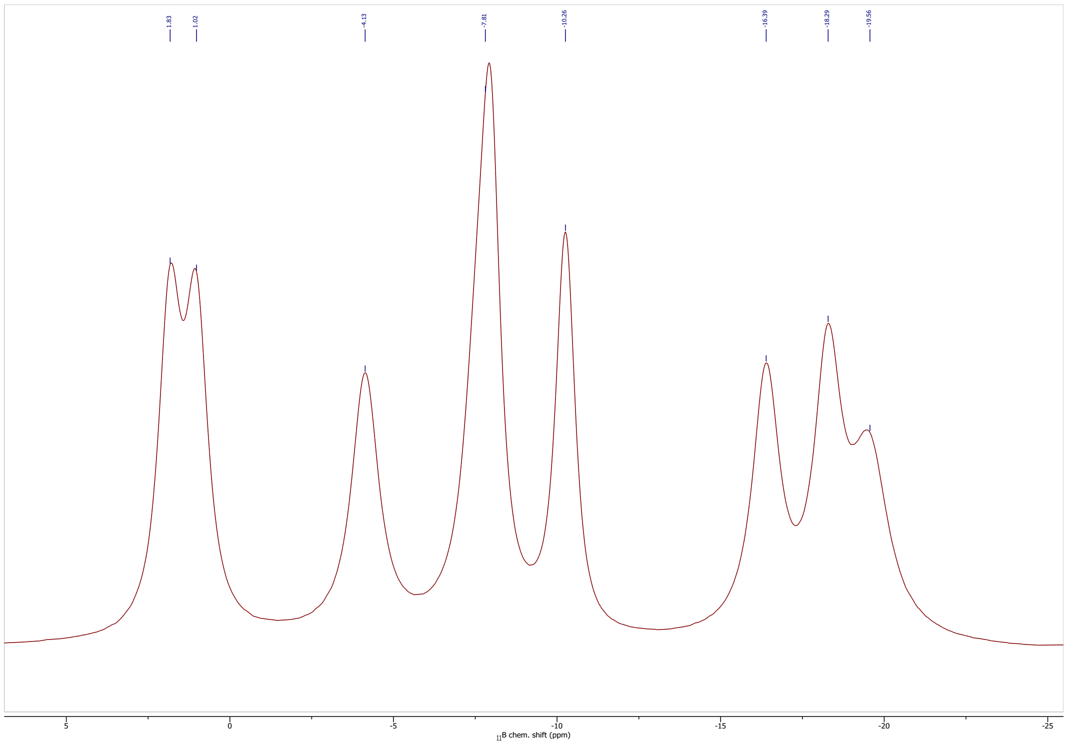

Figure S11. $^{11}B\{^1H\}$ NMR spectrum of **3b** in $CDCl_3$.

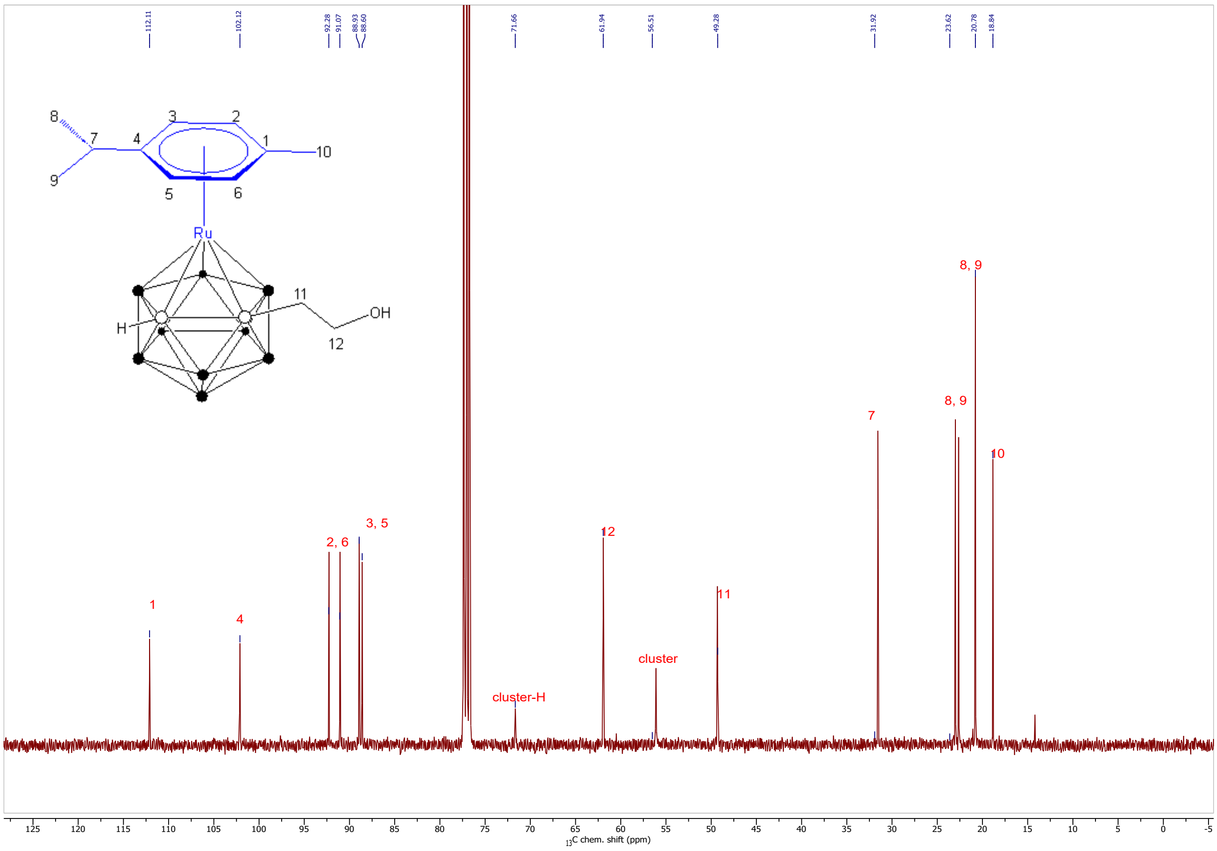

S

Figure S12. $^{13}C\{^1H\}$ NMR spectrum of **3b** in $CDCl_3$

6. Ruthenacarborane-(*p*-cymene)–NSAID Conjugates

[Ru(η^6-*p*-cymene){1-methyl (*R/S*)-2-[4-(2-fluorobiphenyl-4-yl)propanoic acid ester]-*closo*-$C_2B_9H_{10}$}] (**4a**):

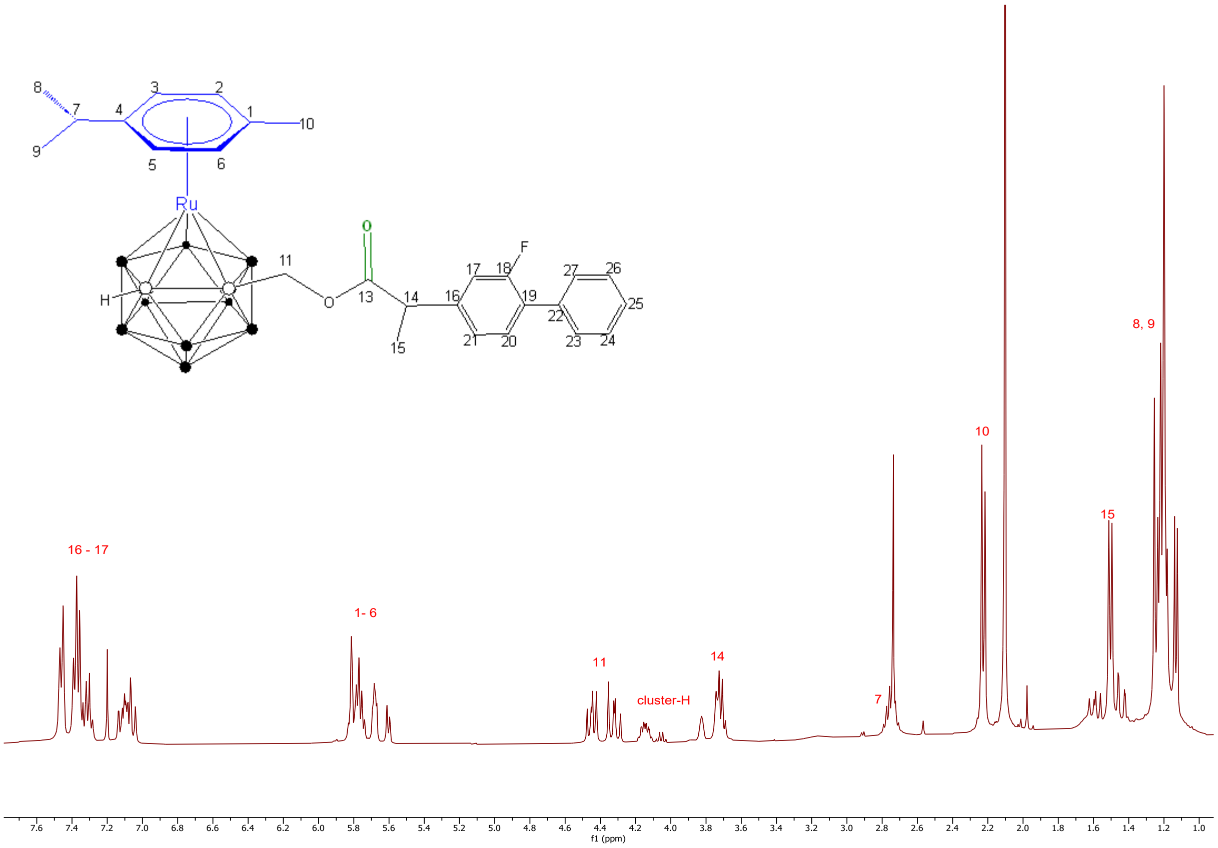

Figure S13. ^{1}H NMR spectrum of **4a** in $CDCl_3$.

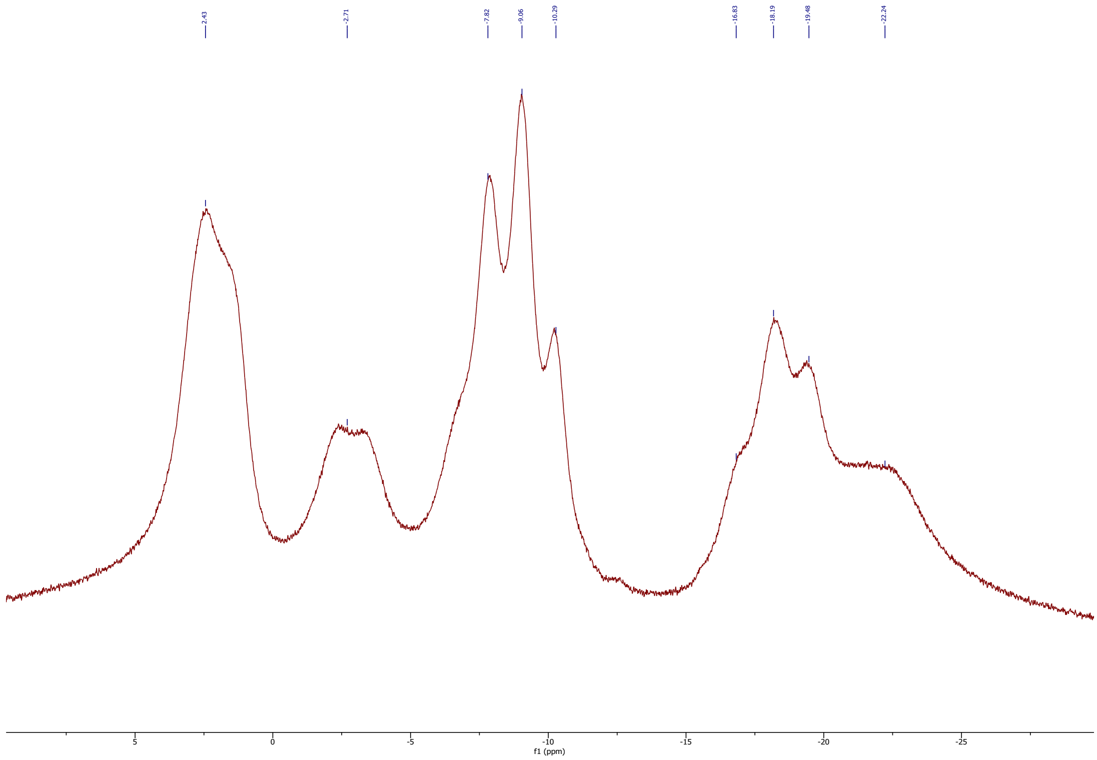

Figure S14. $^{11}B\{^1H\}$ NMR spectrum of **4a** in $CDCl_3$.

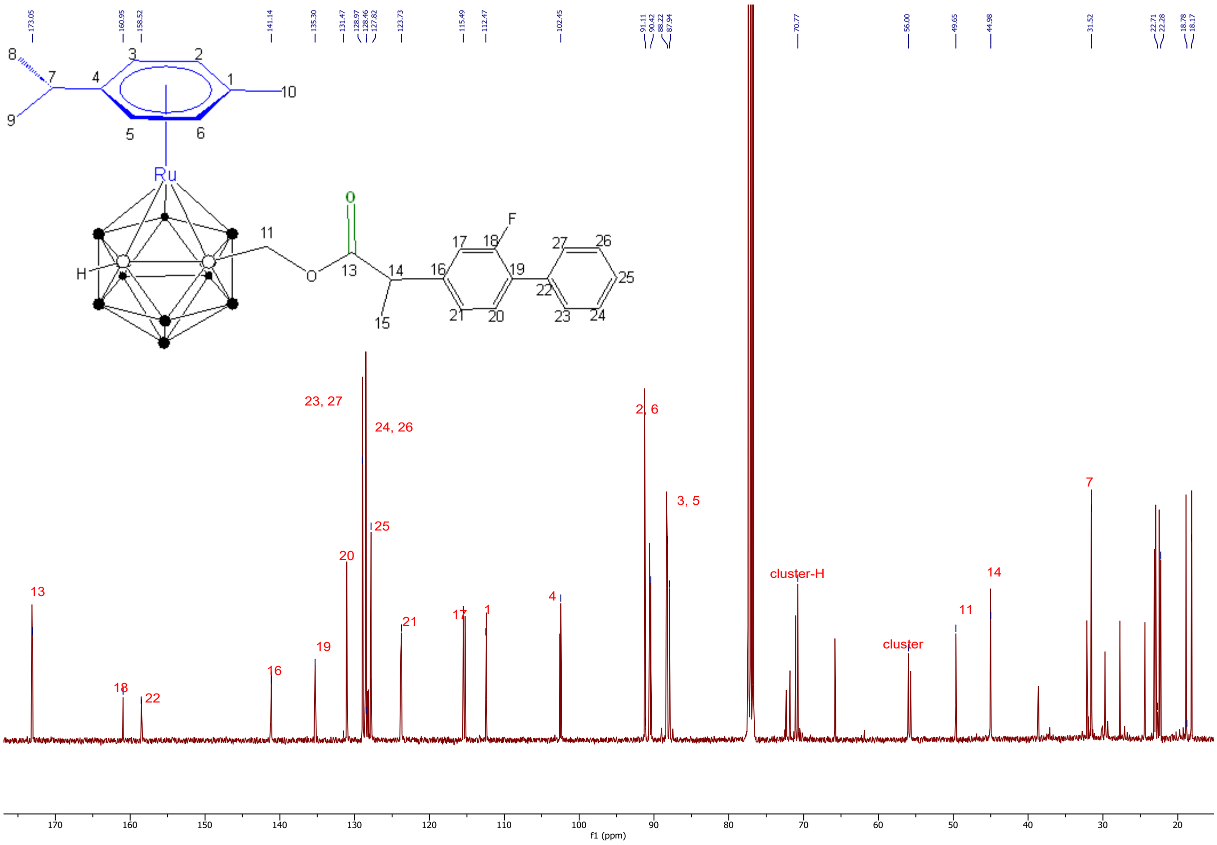

Figure S15. $^{13}C\{^{1}H\}$ NMR spectrum of **4a** in $CDCl_3$.

[Ru(η^6-*p*-cymene){2-ethyl (*R/S*)-2-[4-(2-fluorobiphenyl-4-yl)propanoic acid ester]-*closo*-$C_2B_9H_{10}$}] (**4b**):

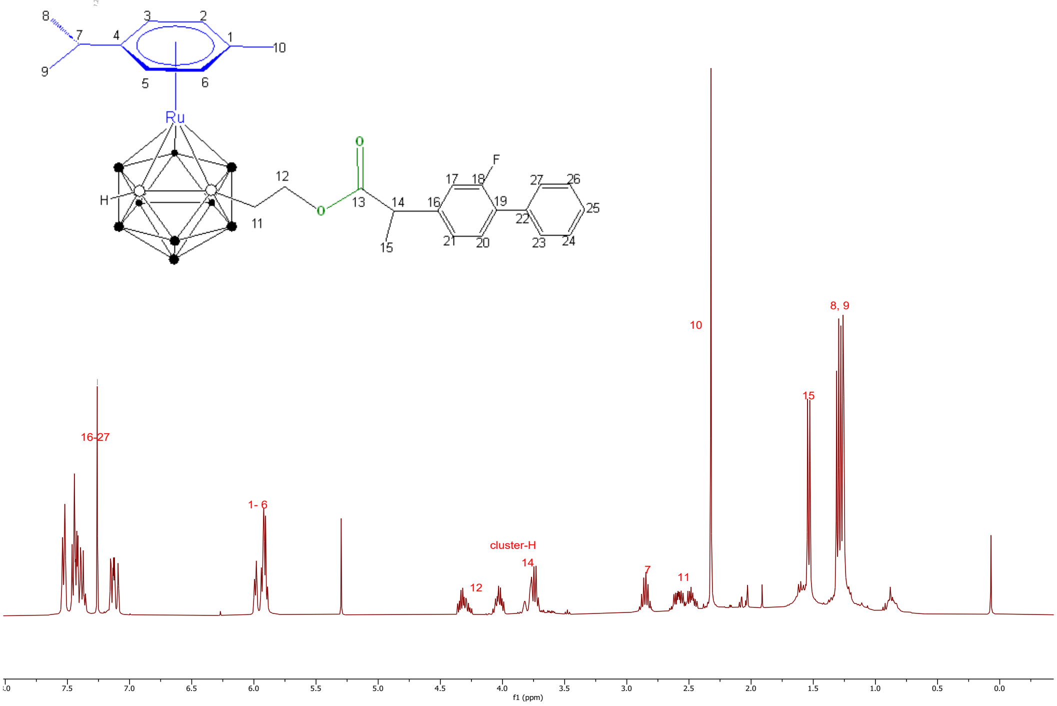

Figure S16. ^{1}H NMR spectrum of **4b** in $CDCl_3$.

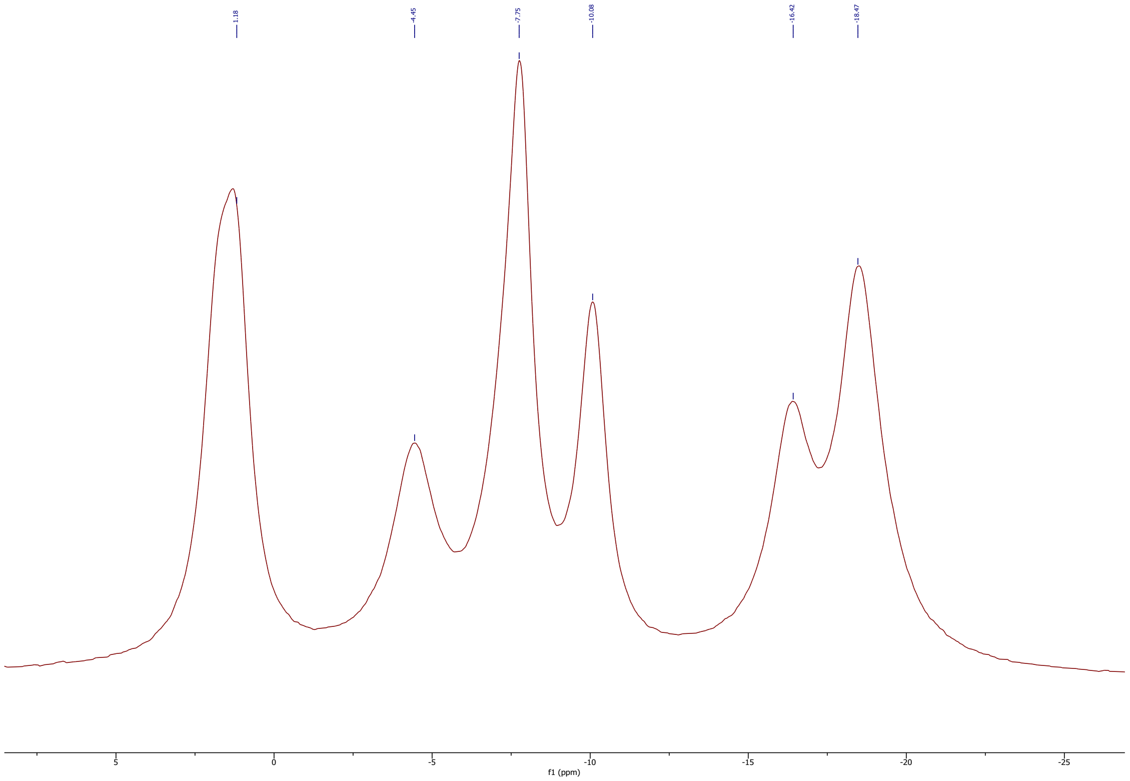

Figure S17. $^{11}B\{^{1}H\}$ NMR spectrum of **4b** in $CDCl_3$.

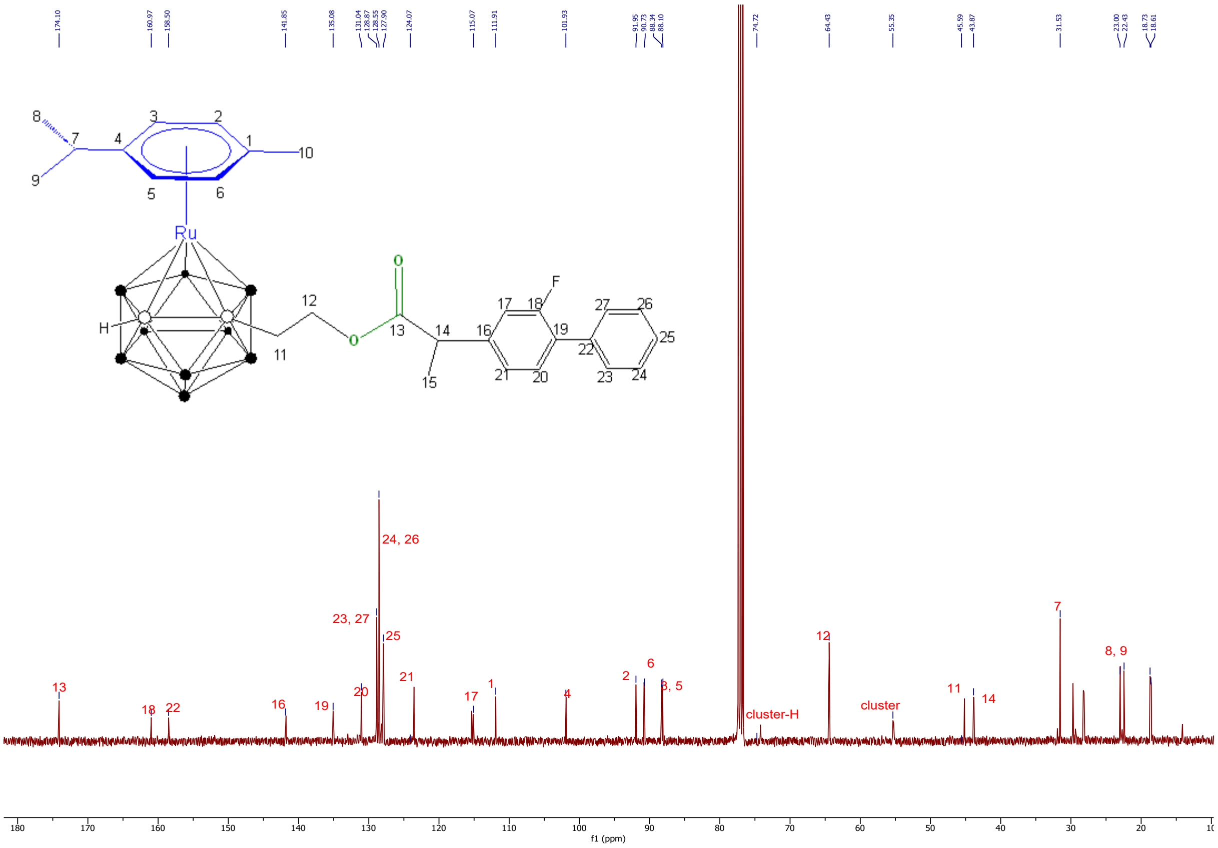

Figure S18. $^{13}C\{^{1}H\}$ NMR spectrum of **4b** in $CDCl_3$.

[Ru(η^6-*p*-cymene){2-ethyl [2-(3-phenoxyphenyl)propanoic acid ester]-*closo*-$C_2B_9H_{10}$}] (**5b**):

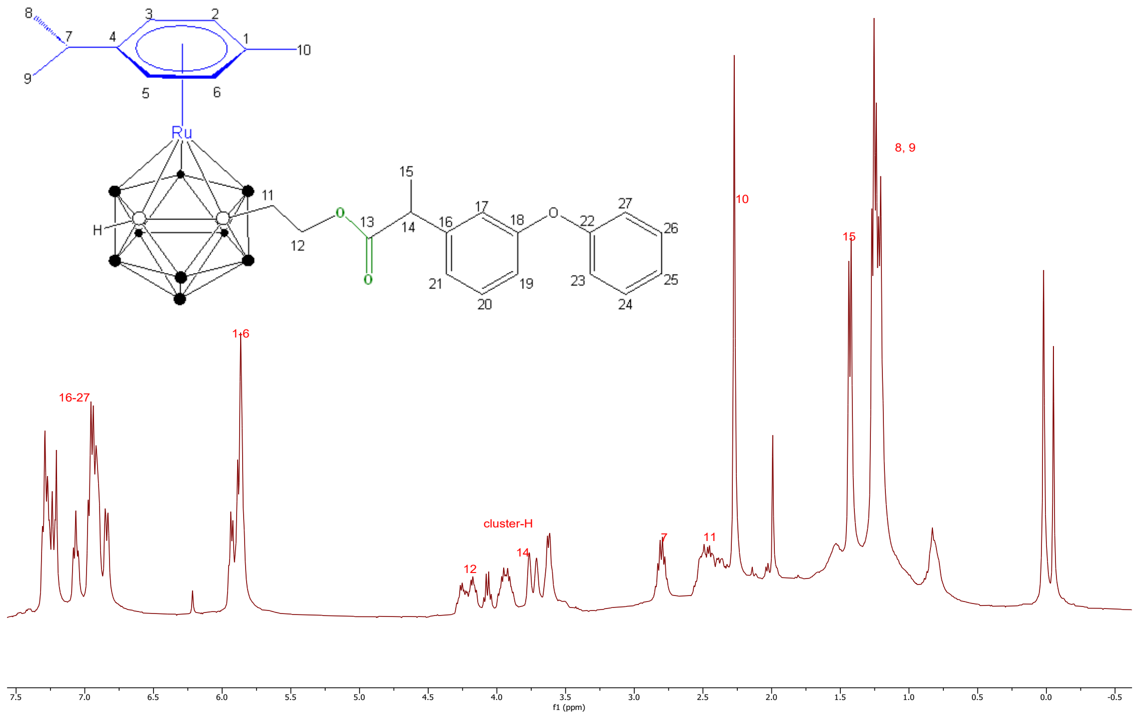

Figure S19. ^{1}H NMR spectrum of **5b** in $CDCl_3$.

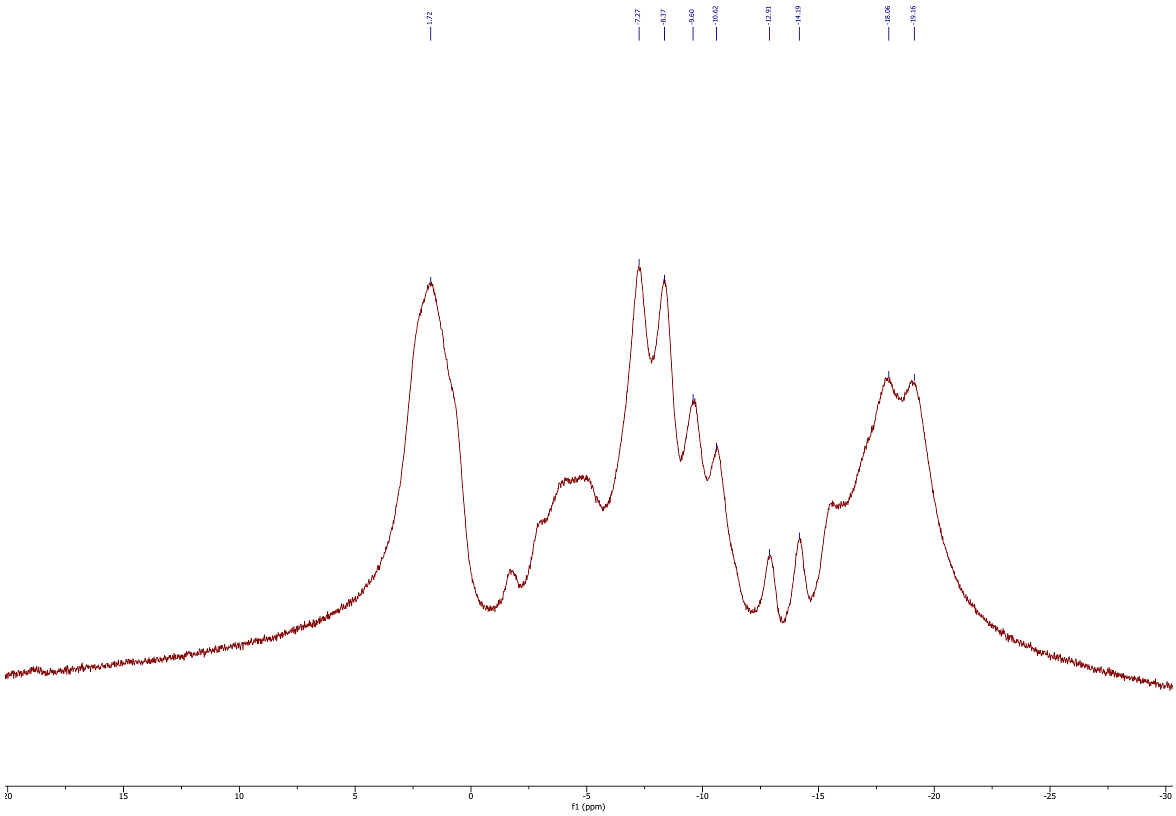

Figure S20. $^{11}B\{^1H\}$ NMR spectrum of **5b** in $CDCl_3$.

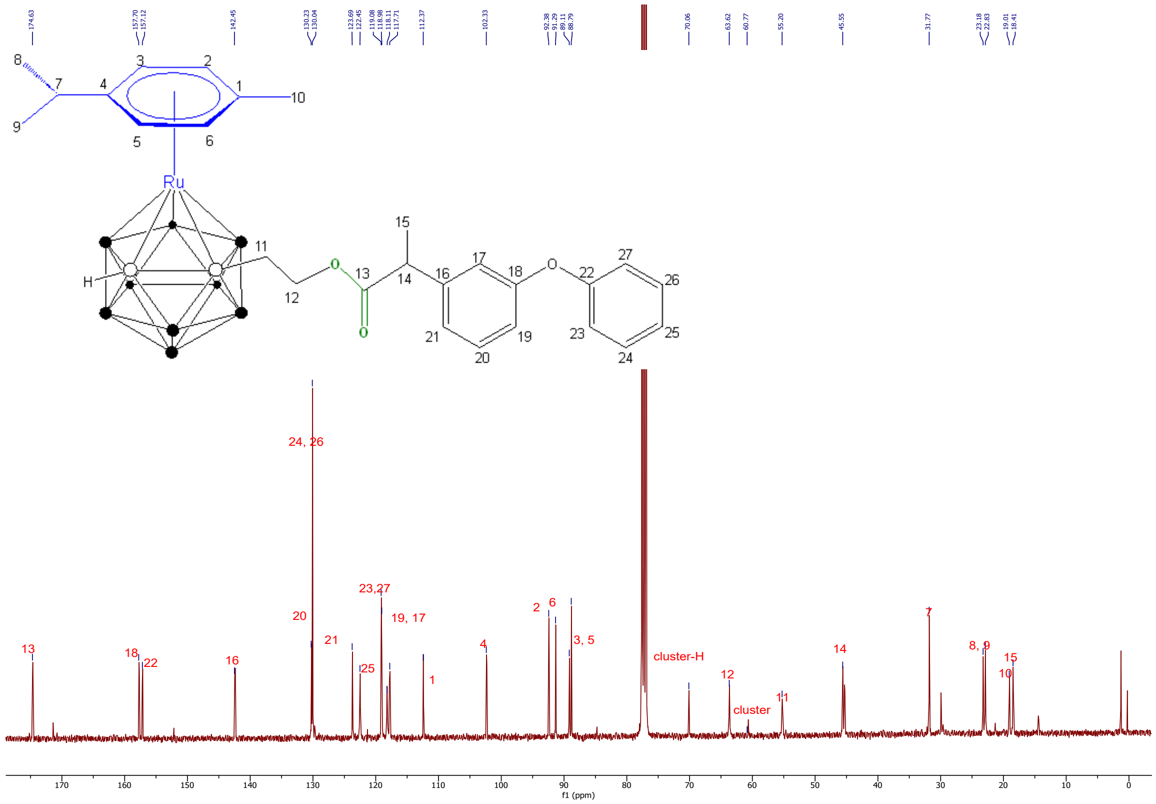

Figure S21. $^{13}C\{^{1}H\}$ NMR spectrum of **5b** in $CDCl_3$.

[Ru(η^6-*p*-cymene){2-ethyl (*R/S*)-2-[4-(2-propylphenyl)propanoic acid ester]-*closo*-$C_2B_9H_{10}$}] (**6b**):

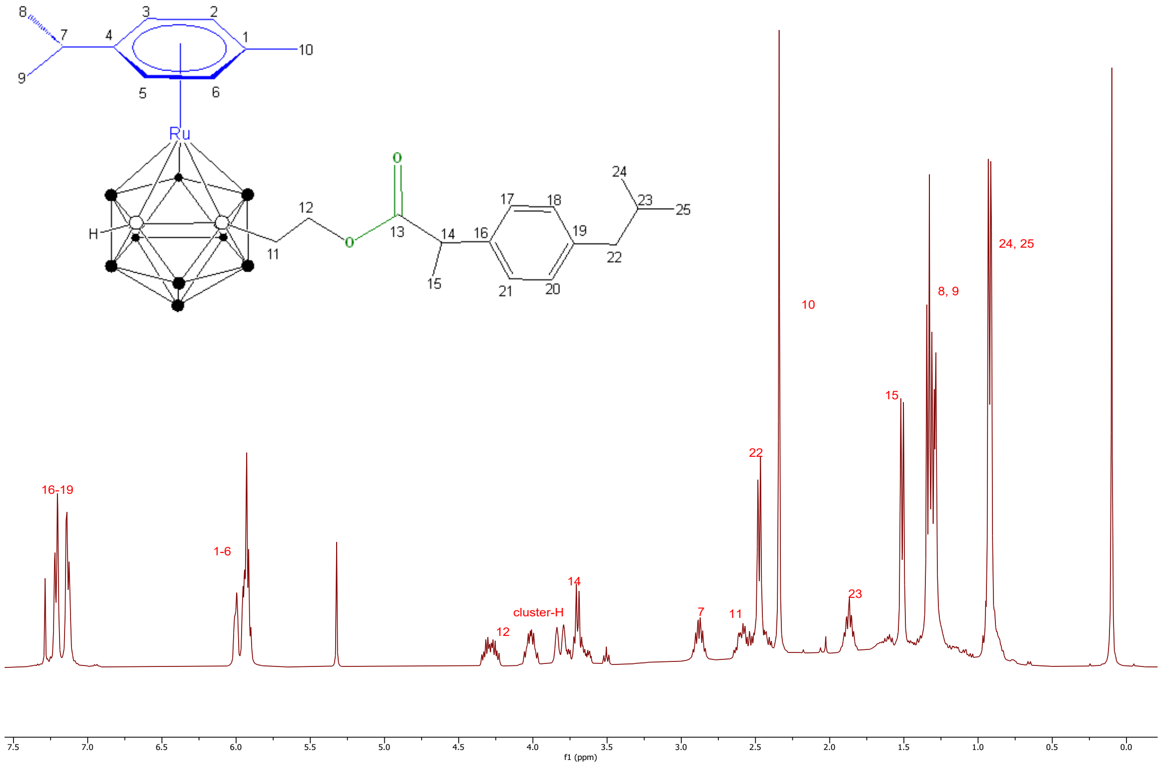

Figure S22. ^{1}H NMR spectrum of **6b** in $CDCl_3$.

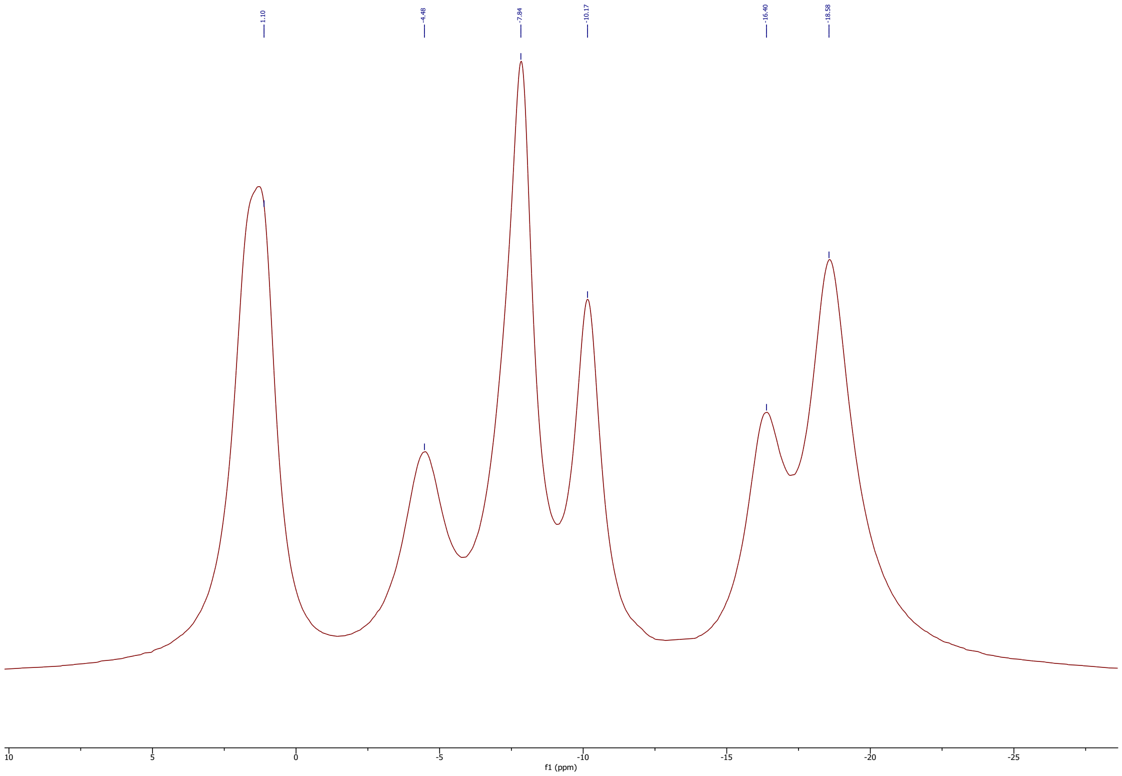

Figure S23. $^{11}B\{^{1}H\}$ NMR spectrum of **6b** in $CDCl_3$.

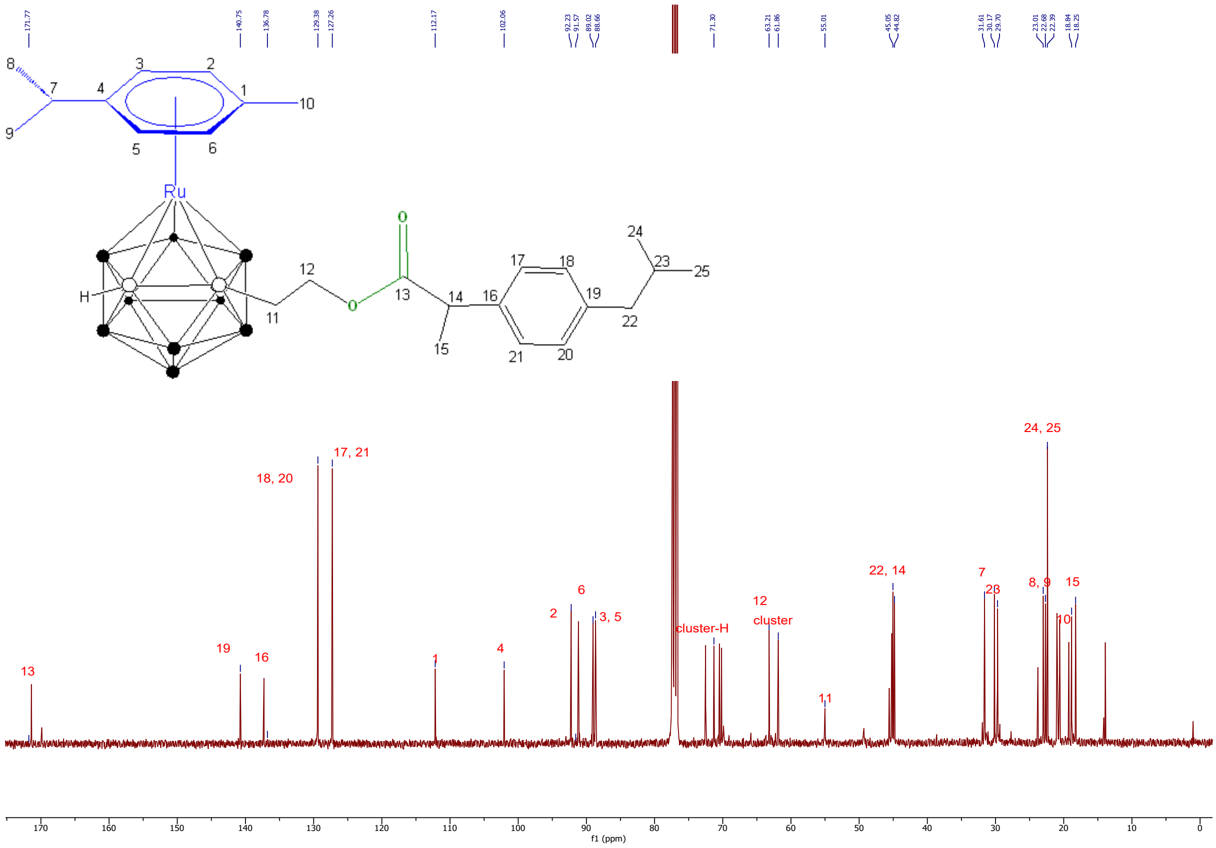

Figure S24. $^{13}C\{^{1}H\}$ NMR spectrum of **6b** in $CDCl_3$.

7. Comparison between ruthenacarborane (**3a**) and ruthenacarborane-(η^6-*p*-cymene)–NSAID conjugates with flurbiprofen (**4a**)

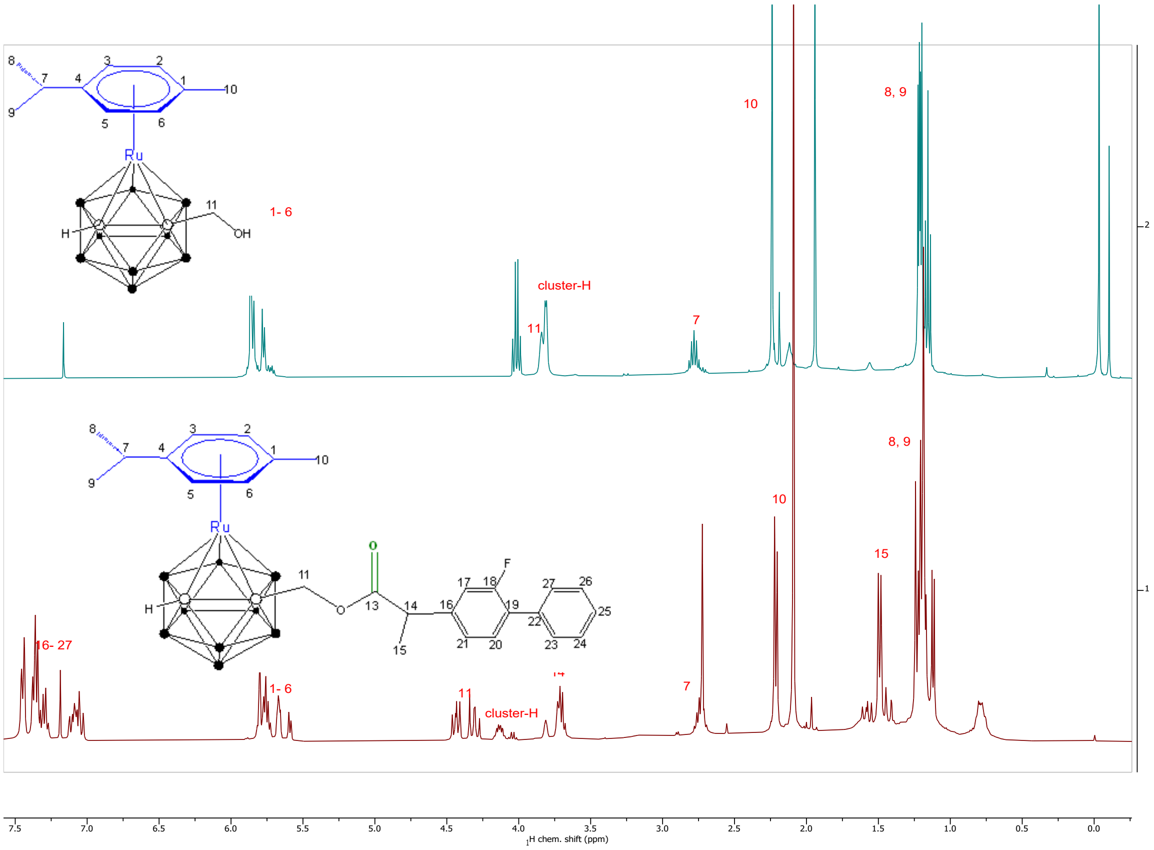

Figure S25. Comparison between ruthenium complexes **3a** (top) and **4a** (bottom).

8. Stability Test

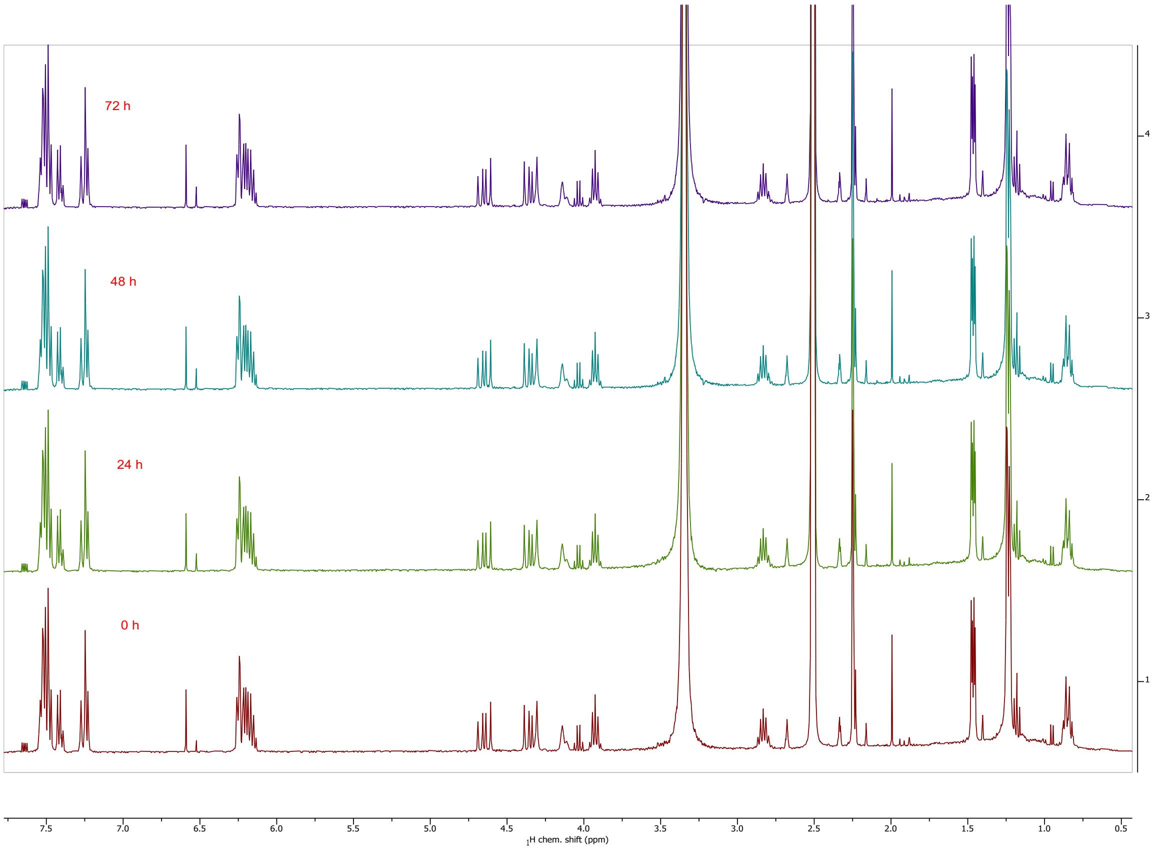

Figure S26. Stability of compound **4a** over 72 h in DMSO-d_6 (^{1}H NMR spectroscopy). Compound **4a** was dissolved in deuterated DMSO and stored at room temperature. ^{1}H NMR spectroscopy was performed at selected time intervals (0, 24, 48 and 72 h).

9. UV-VIS Spectroscopy

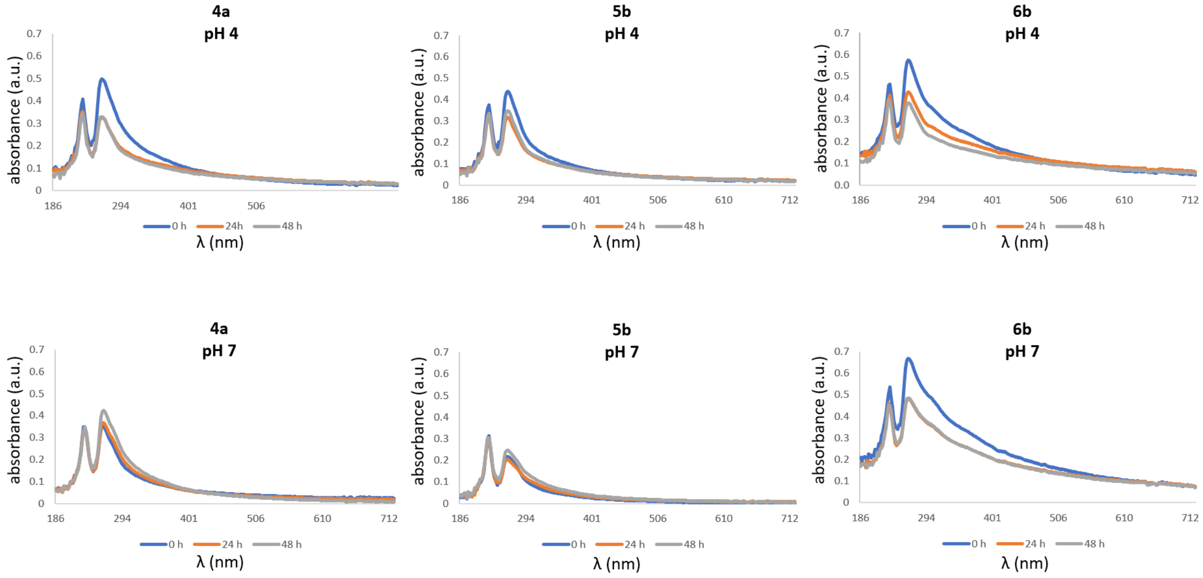

Figure S27. UV/Vis spectra of **4a**, **5b** and **6b** at pH 4 and 7 at 0, 24 and 48 h.

10. Analysis of Purity by High-Performance Liquid Chromatography (HPLC)

Table S1. Results of HPLC analyses of ibuprofen, fenoprofen, flurbiprofen, **2a**, **2b**, **3a**, **3b**, **4a**, **4b**, **5b**, and **6b** at 220 and 254 nm.

Ibuprofen

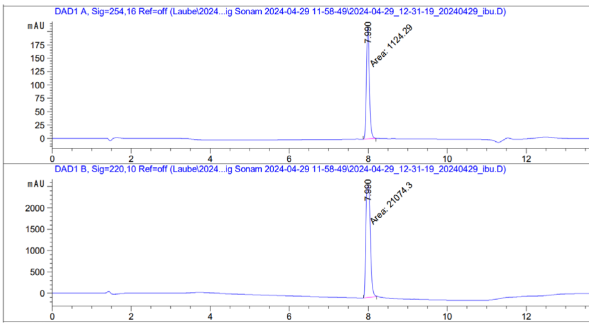

Signal 2: DAD1 A, Sig=254,16 Ref=off

Peak #	RetTime [min]	Type	Width [min]	Area [mAU*s]	Height [mAU]	Area %
1	7.990	MM	0.0890	1124.28564	210.52943	100.00[illegible]

Totals : 1124.28564 210.52943

Signal 3: DAD1 B, Sig=220,10 Ref=off

Peak #	RetTime [min]	Type	Width [min]	Area [mAU*s]	Height [mAU]	Area %
1	7.990	MM	0.1330	2.10743e4	2640.41724	100.00[illegible]

Totals : 2.10743e4 2640.41724

Fenoprofen

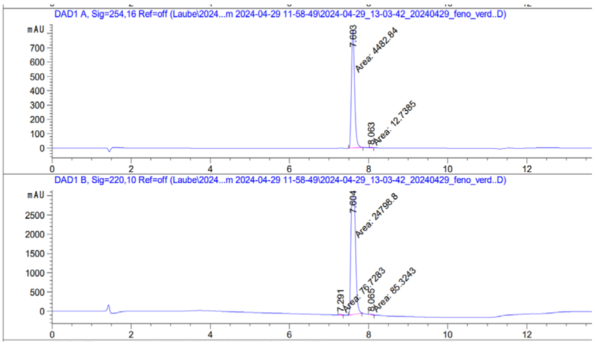

Signal 2: DAD1 A, Sig=254,16 Ref=off

Peak #	RetTime [min]	Type	Width [min]	Area [mAU*s]	Height [mAU]	Area %
1	7.603	MM	0.0905	4482.83789	825.55927	99.7166
2	8.063	MM	0.0762	12.73851	2.78574	0.2834

Totals : 4495.57640 828.34501

Signal 3: DAD1 B, Sig=220,10 Ref=off

Peak #	RetTime [min]	Type	Width [min]	Area [mAU*s]	Height [mAU]	Area %
1	7.291	MM	0.0811	76.72831	15.77770	0.3074
2	7.604	MM	0.1344	2.47988e4	3074.15088	99.3508
3	8.065	MM	0.0810	85.32430	17.55629	0.3418

Totals : 2.49609e4 3107.48487

Flurbiprofen

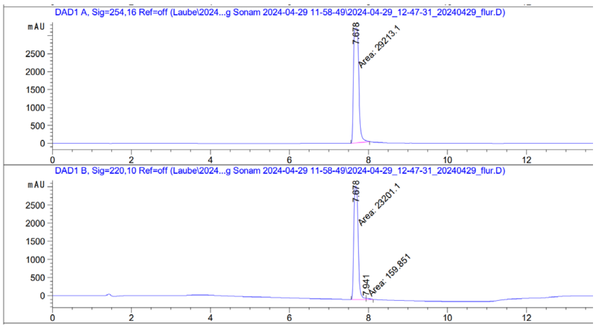

Signal 2: DAD1 A, Sig=254,16 Ref=off

Peak #	RetTime [min]	Type	Width [min]	Area [mAU*s]	Height [mAU]	Area %
1	7.678	MM	0.1520	2.92131e4	3202.78052	100.0000

Totals : 2.92131e4 3202.78052

Signal 3: DAD1 B, Sig=220,10 Ref=off

Peak #	RetTime [min]	Type	Width [min]	Area [mAU*s]	Height [mAU]	Area %
1	7.678	MF	0.1237	2.32011e4	3126.52539	99.3157
2	7.941	FM	0.0971	159.85115	27.43852	0.6843

Totals : 2.33610e4 3153.96391

2a

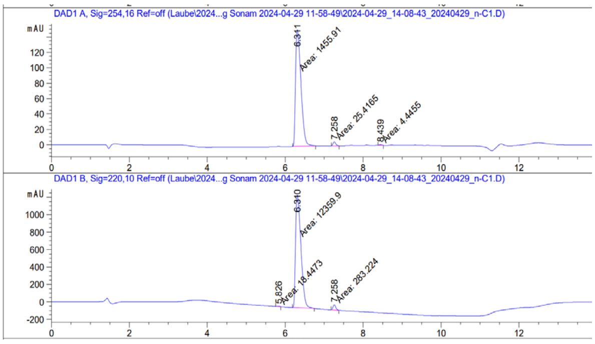

Signal 2: DAD1 A, Sig=254,16 Ref=off

Peak #	RetTime [min]	Type	Width [min]	Area [mAU*s]	Height [mAU]	Area %
1	6.311	MM	0.1597	1455.90918	151.98502	97.9901
2	7.258	MM	0.0808	25.41654	5.24392	1.7107
3	8.439	MM	0.0781	4.44550	9.48431e-1	0.2992

Totals : 1485.77122 158.17736

Signal 3: DAD1 B, Sig=220,10 Ref=off

Peak #	RetTime [min]	Type	Width [min]	Area [mAU*s]	Height [mAU]	Area %
1	5.826	MM	0.0761	18.44734	4.03816	0.1457
2	6.310	MM	0.1592	1.23599e4	1294.25122	97.6174
3	7.258	MM	0.0792	283.22430	59.61414	2.2369

Totals : 1.26616e4 1357.90352

2b

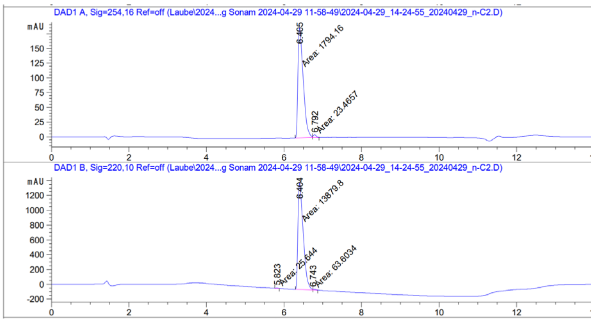

Signal 2: DAD1 A, Sig=254,16 Ref=off

Peak #	RetTime [min]	Type	Width [min]	Area [mAU*s]	Height [mAU]	Area %
1	6.405	MF	0.1594	1794.15588	187.58167	98.7090
2	6.792	FM	0.0845	23.46570	4.63022	1.2910

Totals : 1817.62159 192.21188

Signal 3: DAD1 B, Sig=220,10 Ref=off

Peak #	RetTime [min]	Type	Width [min]	Area [mAU*s]	Height [mAU]	Area %
1	5.823	MM	0.0738	25.64400	5.78967	0.1836
2	6.404	MF	0.1602	1.38798e4	1444.28040	99.3611
3	6.743	FM	0.0885	63.60336	11.97167	0.4553

Totals : 1.39690e4 1462.04174

3a

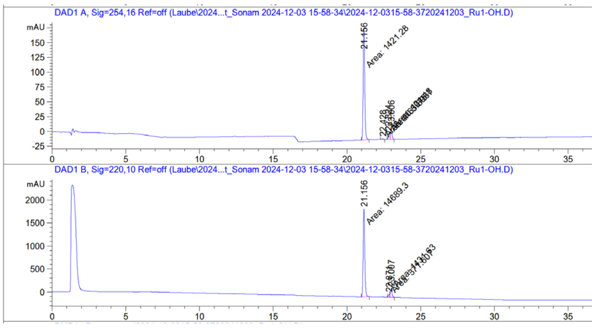

Signal 2: DAD1 A, Sig=254,16 Ref=off

Peak #	RetTime [min]	Type	Width [min]	Area [mAU*s]	Height [mAU]	Area %
1	21.156	MM	0.1248	1421.27747	189.77274	87.3583
2	22.428	MM	0.1407	10.59751	1.25527	0.6514
3	22.824	MF	0.1053	63.89671	10.10933	3.9274
4	23.006	FM	0.1266	131.17989	17.26437	8.0629

Totals : 1626.95157 218.40170

Signal 3: DAD1 B, Sig=220,10 Ref=off

Peak #	RetTime [min]	Type	Width [min]	Area [mAU*s]	Height [mAU]	Area %
1	21.156	MM	0.1285	1.46893e4	1905.62329	89.0663
2	22.871	MF	0.0993	371.60736	62.36855	2.2532
3	23.007	FM	0.1258	1431.63281	189.72269	8.6805

Totals : 1.64926e4 2157.71452

3b

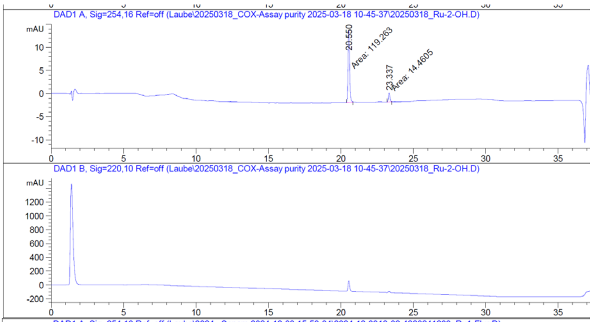

Signal 2: DAD1 A, Sig=254,16 Ref=off

Peak #	RetTime [min]	Type	Width [min]	Area [mAU*s]	Height [mAU]	Area %
1	20.550	MM	0.1259	119.26324	15.78806	89.1863
2	23.337	MM	0.1236	14.46047	1.94960	10.8137

Totals : 133.72372 17.73766

Signal 3: DAD1 B, Sig=220,10 Ref=off

4a

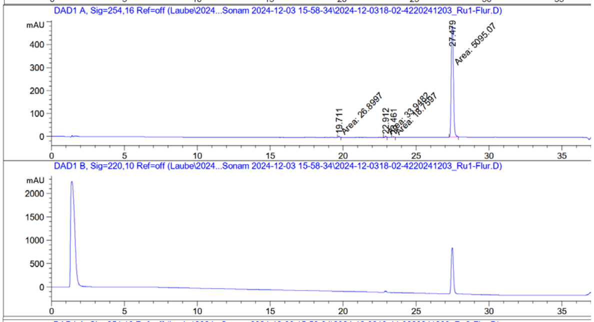

Signal 2: DAD1 A, Sig=254,16 Ref=off

Peak #	RetTime [min]	Type	Width [min]	Area [mAU*s]	Height [mAU]	Area %
1	19.711	MM	0.1192	26.89965	3.76102	0.5198
2	22.912	MM	0.1257	33.94823	4.50294	0.6560
3	23.461	MM	0.1277	18.75972	2.44882	0.3625
4	27.479	MM	0.1752	5095.06641	484.57080	98.4616

Totals : 5174.67401 495.28359

Signal 3: DAD1 B, Sig=220,10 Ref=off

4b

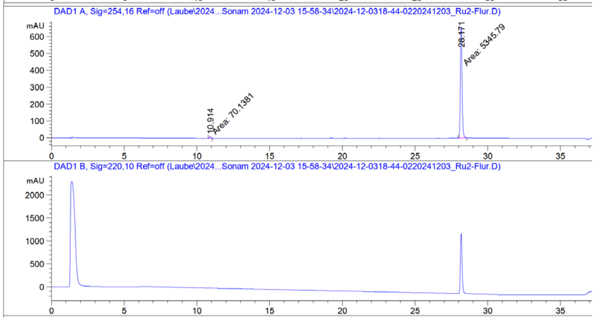

Signal 2: DAD1 A, Sig=254,16 Ref=off

Peak #	RetTime [min]	Type	Width [min]	Area [mAU*s]	Height [mAU]	Area %
1	10.914	MM	0.1183	70.13813	9.87747	1.2950
2	28.171	MM	0.1346	5345.78613	661.90588	98.7050

Totals : 5415.92426 671.78335

Signal 3: DAD1 B, Sig=220,10 Ref=off

5b

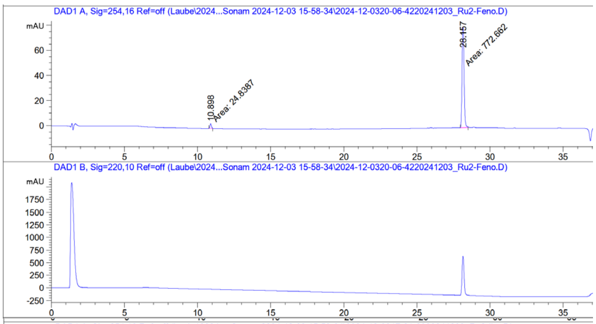

Signal 2: DAD1 A, Sig=254,16 Ref=off

Peak #	RetTime [min]	Type	Width [min]	Area [mAU*s]	Height [mAU]	Area %
1	10.898	MM	0.1141	24.83875	3.62736	3.1146
2	28.157	MM	0.1598	772.66199	80.60784	96.8854
Totals :				797.50073	84.23520	

Signal 3: DAD1 B, Sig=220,10 Ref=off

6b

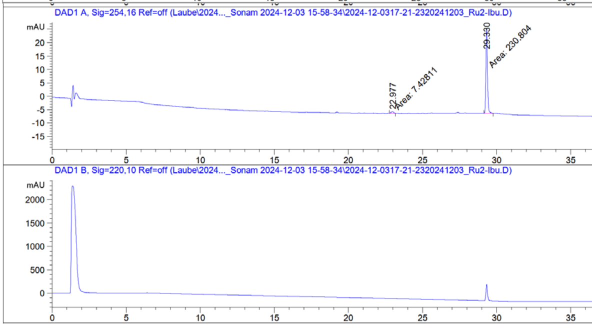

Signal 2: DAD1 A, Sig=254,16 Ref=off

Peak #	RetTime [min]	Type	Width [min]	Area [mAU*s]	Height [mAU]	Area %
1	22.977	MM	0.2084	7.42811	5.94062e-1	3.1180
2	29.330	MM	0.1206	230.80376	31.90118	96.8820
Totals :				238.23187	32.49524	

Signal 3: DAD1 B, Sig=220,10 Ref=off

11. Evaluation for COX Inhibition

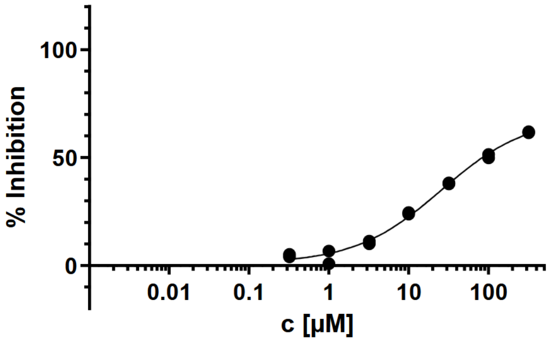

Figure S28. COX-2 inhibition data of **2b**.

12. Dynamic Light Scattering (DLS) Measurements

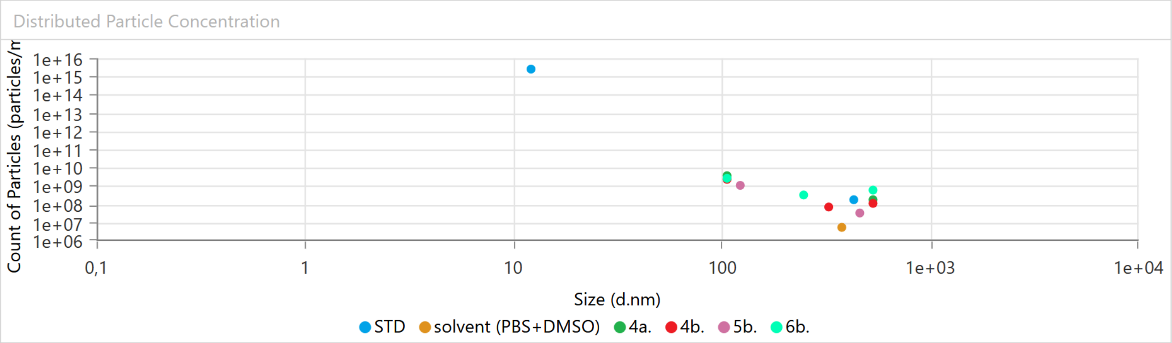

Figure S29: Count of particles of compound **4a**, **4b**, **5b**, and **6b** STD (200 nm) and solvent (DMSO + PBS).

Table S2. Total number concentration and particles concentration peak of compound **4a**, **4b**, **5b**, and **6b** particles (200 nm) and solvent (DMSO + PBS).

Parameter Table

	Sample Name	Particle Concentration Peak One (particles/ml)	Total Number Concentration (particles/ml)
1	STD	2,42E+15	2,42E+15
2	solvent (PBS+DMSO)	5,499E+06	5,499E+06
3	4a.	3,828E+09	4,015E+09
4	4b.	2,172E+09	2,351E+09
5	5b.	1,057E+09	1,093E+09
6	6b.	2,906E+09	3,772E+09

13. Cyclic Voltammetry (CV)

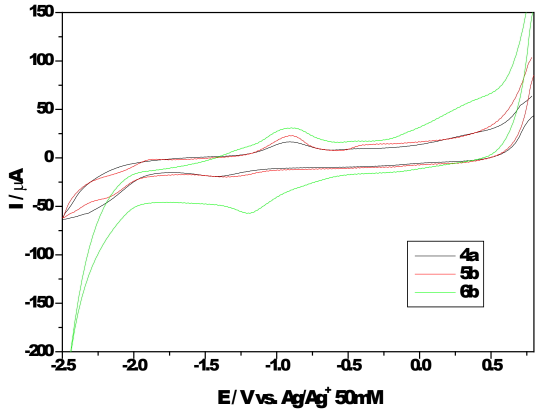

Figure S30. Comparison of CVs obtained for **4a**, **5b**, and **6b**.

Cyclic voltammograms obtained at a Pt electrode with ruthenacarborane-(η^6-*p*-cymene)–NSAID conjugates ($5x10^{-4}$ M). Experimental conditions: 0.1 M [*n*-Bu_4N][PF_6] electrolyte in DMSO solution; scan rate 0.1 V s^{-1}.

4a

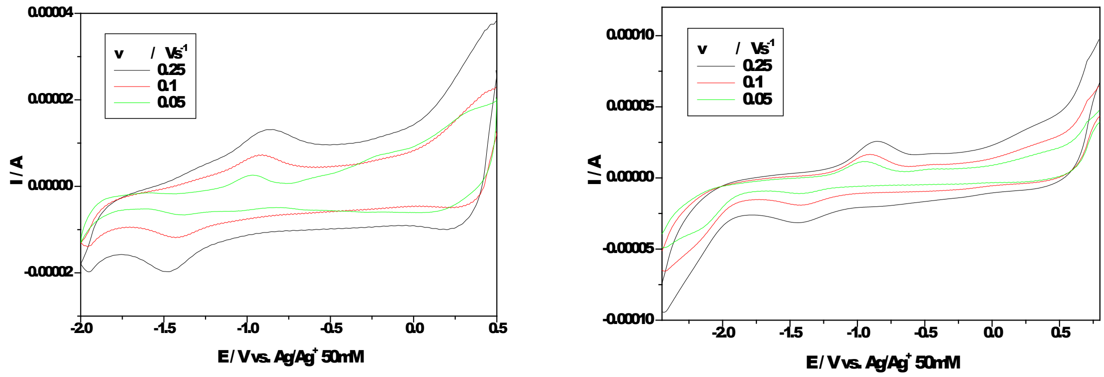

5b

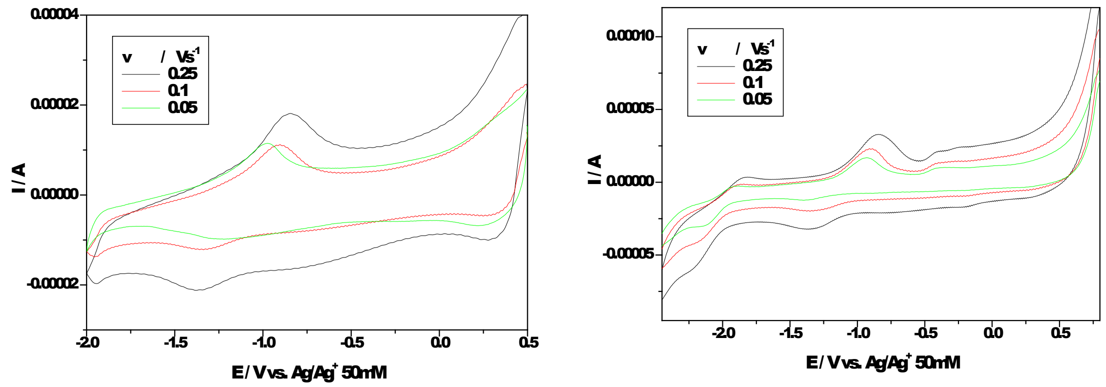

6b

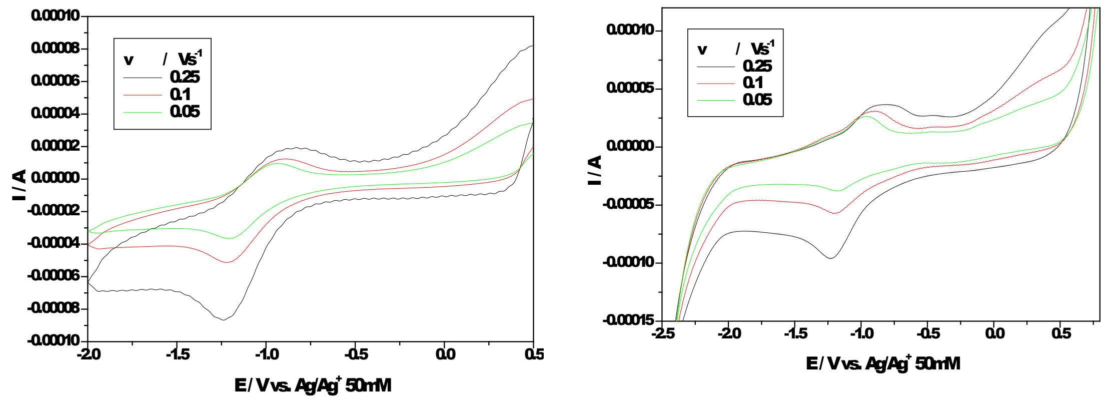

Figure S31. Cyclic voltammograms at different current of **4a**, **5b**, and **6b**.

Peak parameters:

All the potentials presented here are *vs.* Ag/$AgNO_3$ (50 mM) for all the ruthenacarborane-(η^6-*p*-cymene)–NSAID conjugates (in DMSO with 0.1M [*n*-Bu_4N][PF_6]).

The potential against other reference electrodes can be obtained using one of the following relations.

E[V vs NHE] = E[V vs Ag/Ag^+] +0.763

E[V vs Fc^+/Fc] = E[V vs Ag/Ag^+] +0.123

14. Cell Viability

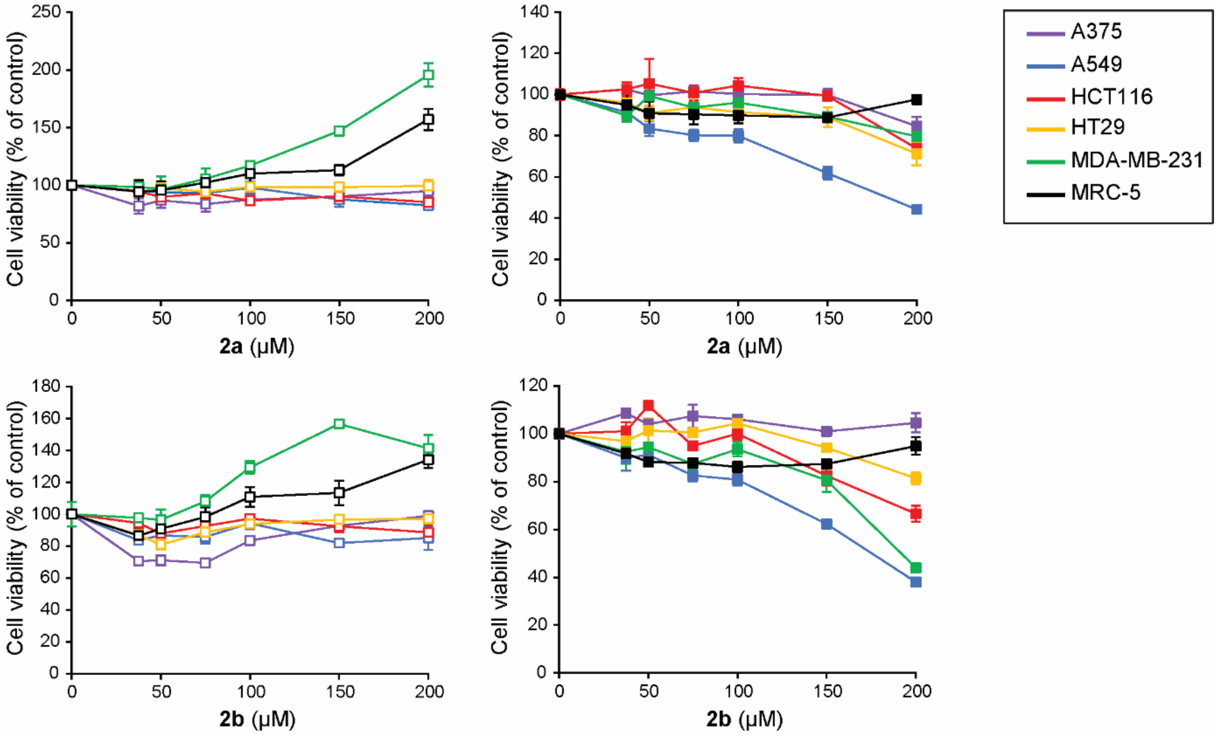

Figure S32. The effect of **2a** and **2b** on the viability of human cancer cell lines and primary transformed lung embryonal fibroblasts. A375 (4×10^3 cells/well), HCT116 (5×10^3 cells/well), HT29 (8×10^3 cells/well), A549 (2×10^3 cells/well), MDA-MB-231 (3×10^3 cells/well), and MRC5 (1×10^4 cells/well) were treated with wide range of concentrations of **2a** and **2b** for 72h, after which the viability was evaluated by MTT (empty squares) and crystal violet test (filled squares). The data are presented as mean ± SD from one representative of three independent experiments and are expressed as a percentage of the viability of untreated cells, which are considered as 100%.

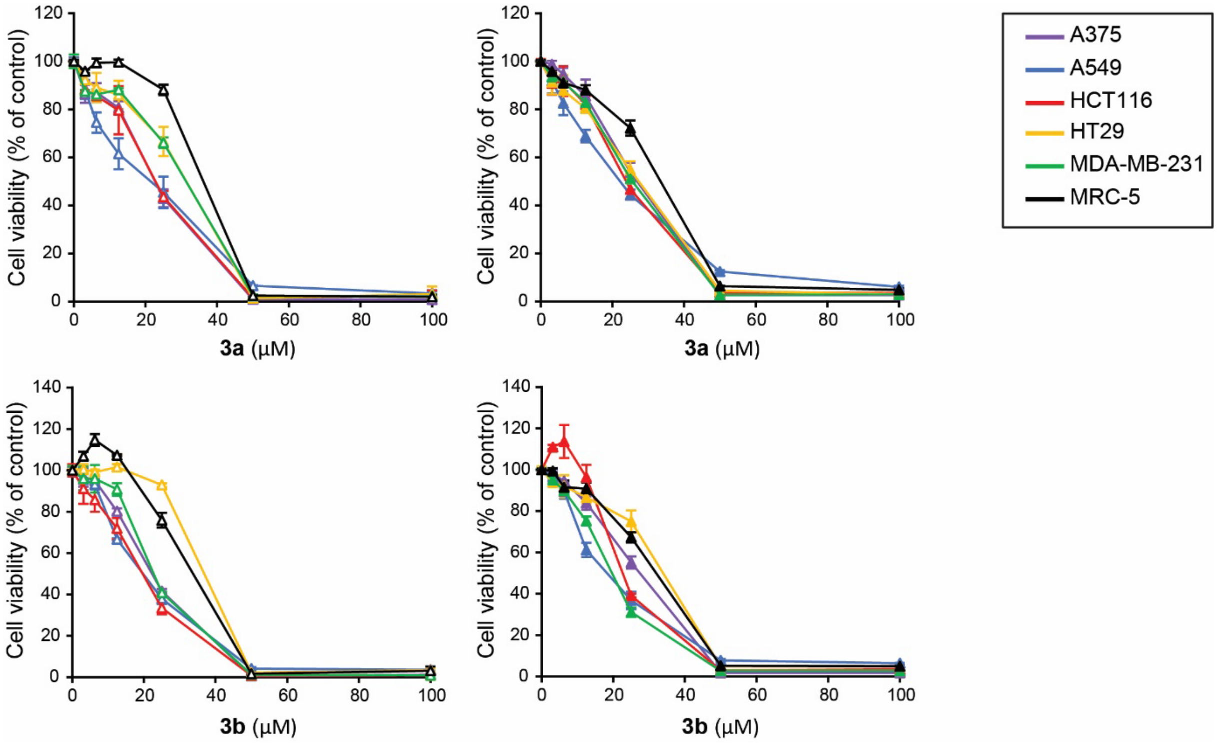

Figure S33. The effect of **3a** and **3b** on the viability of human cancer cell lines and primary transformed lung embryonal fibroblasts.

A375 (4×10^3 cells/well), HCT116 (5×10^3 cells/well), HT29 (8×10^3 cells/well), A549 (2×10^3 cells/well), MDA-MB-231 (3×10^3 cells/well), and MRC-5 (1×10^4 cells/well) were treated with wide range of concentrations of **3a** and **3b** for 72h, after which the viability was evaluated by MTT (empty triangles) and crystal violet test (filled triangles). The data are presented as mean ± SD from one representative of three independent experiments and are expressed as a percentage of the viability of untreated cells, which are considered as 100%.

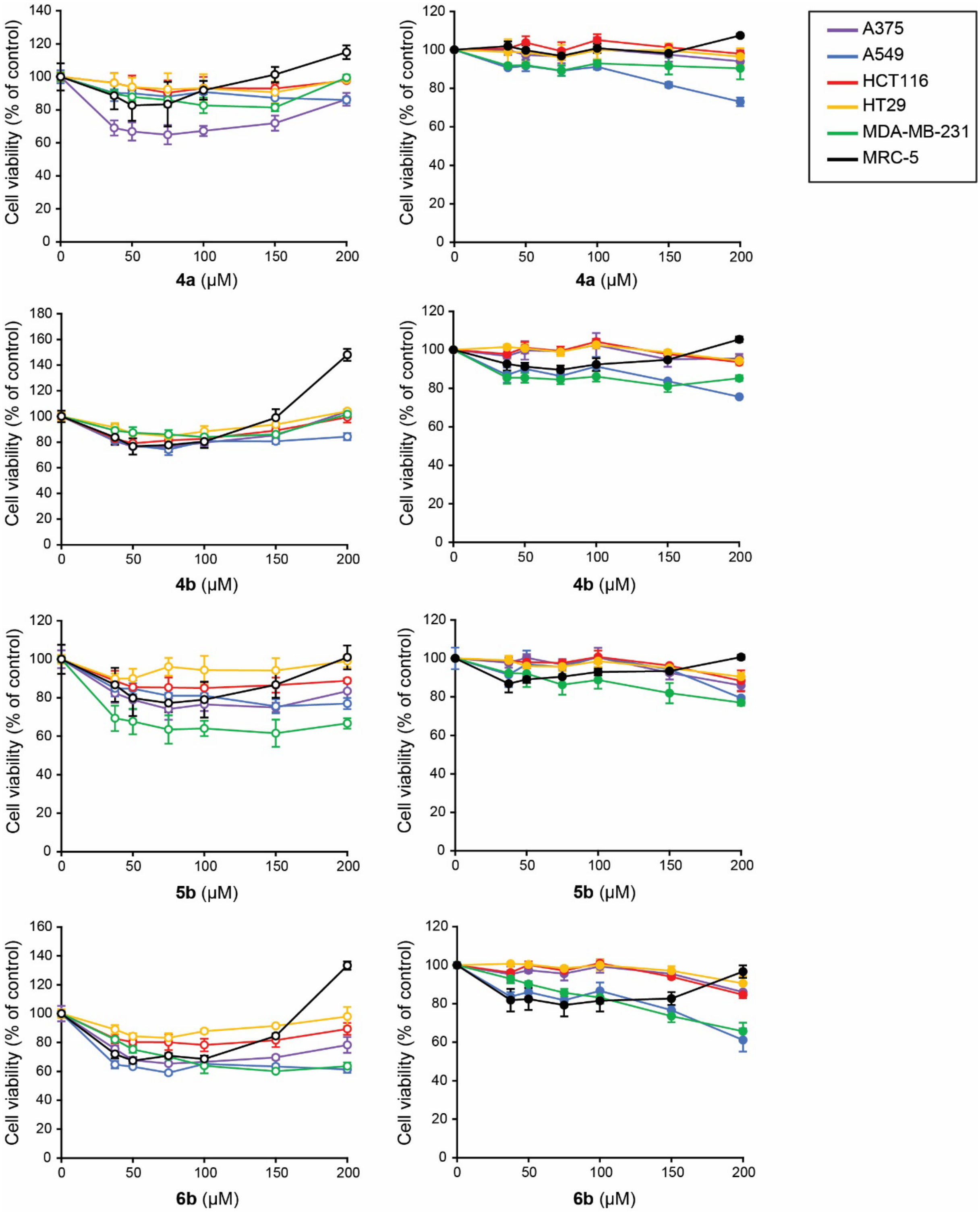

Figure S34. The effect of **4a**, **4b**, **5b**, and **6b** on the viability of human cancer cell lines and primary transformed lung embryonal fibroblasts

A375 (4×10^3 cells/well), HCT116 (5×10^3 cells/well), HT29 (8×10^3 cells/well), A549 (2×10^3 cells/well), MDA-MB-231 (3×10^3 cells/well), and MRC-5 (1×10^4 cells/well) were treated with wide range of concentrations of **4a**, **4b**, **5b**, and **6b** for 72 h, after which the viability was evaluated by MTT (empty circles) and crystal violet test (filled circles). The data are presented as mean ± SD from one representative of three independent experiments and are expressed as a percentage of the viability of untreated cells, which are considered as 100%.

15. Stability Test by UPLC-MS

Table S3: Stability test by UPLC-MS. Compounds, retention times and m/z.

Compound	t_R in min	m/z used for filter in Da	ion
**3a**	3.0–3.1	381	$[M + H - H_2O]^+$
**3b**	3.0–3.1	412	$[M + H]^+$
**4a**	5.1–5.2	643	$[M + H_2O + H]^+$
**5b**	5.3–5.4	636	$[M + H]^+$
**6b**	5.6–5.7	619	$[M + H_2O + H]^+$

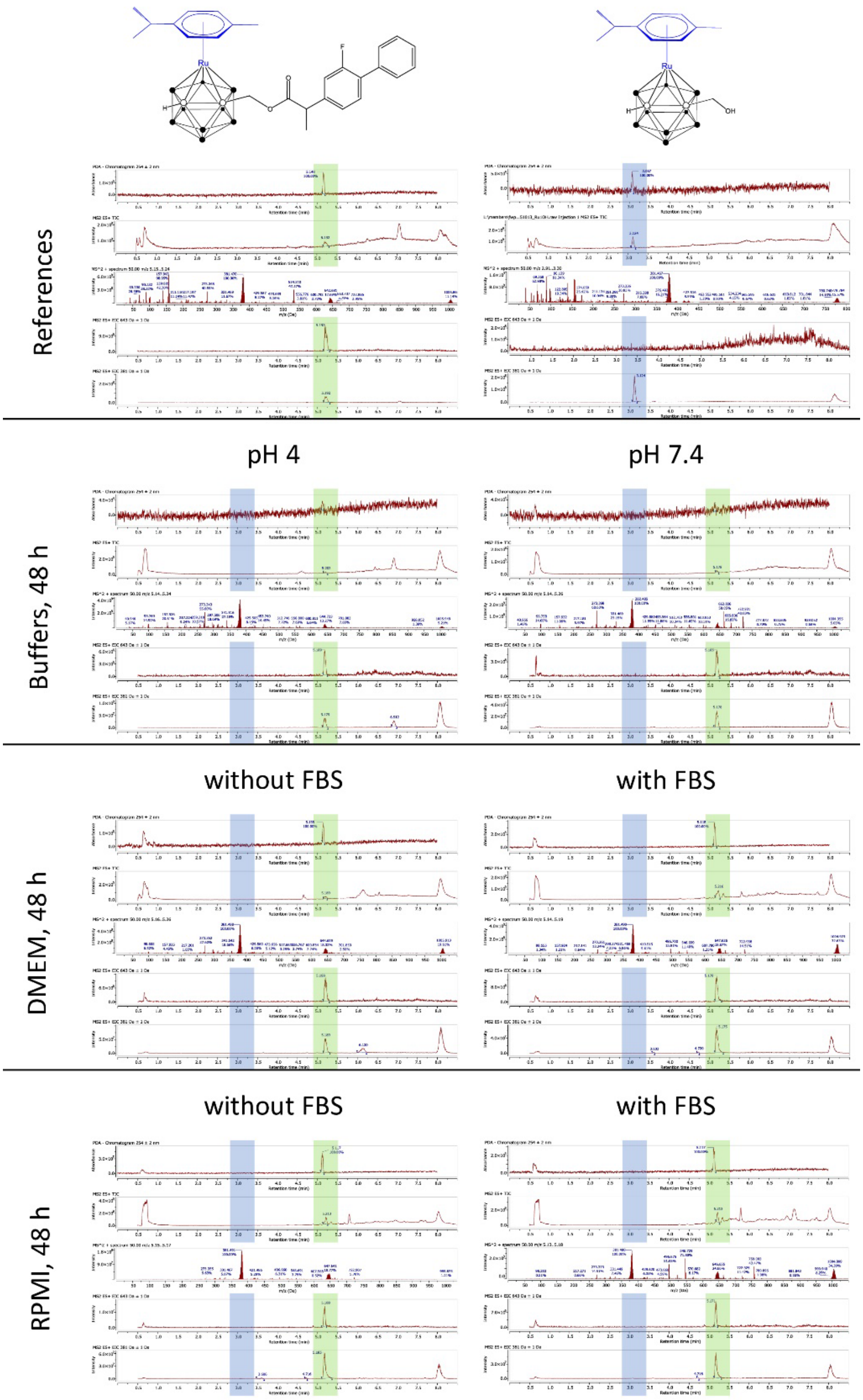

Figure S35: Stability studies of compound **4a** in various media over 48 h.

Each panel is the result of the injection of one sample and is made up of the following chromatograms and spectra (top to bottom): 1) PDA trace at 254 ± 2 nm, 2) total ion count (TIC) chromatogram with integrated peak of **3a** (top right panel) or **4a** (all others), 3) mass spectrum extracted from indicated peak in TIC, 4) TIC filtered for mass peak of **4a** (643 ± 1 Da), 5) TIC filtered for mass peak of **3a** (381 ± 1 Da). The retention times of **4a** (green) and **3a** (blue) are highlighted in all chromatograms. Panels are in the following order: top row) references **4a** (left) and **3a** (right), second row) samples from pH 4 (left) and pH 7.4 (right), third row) samples from DMEM without (left) and with (right) addition of 10 % FBS, bottom row) samples from RPMI without (left) and with (right) addition of 10 % FBS.

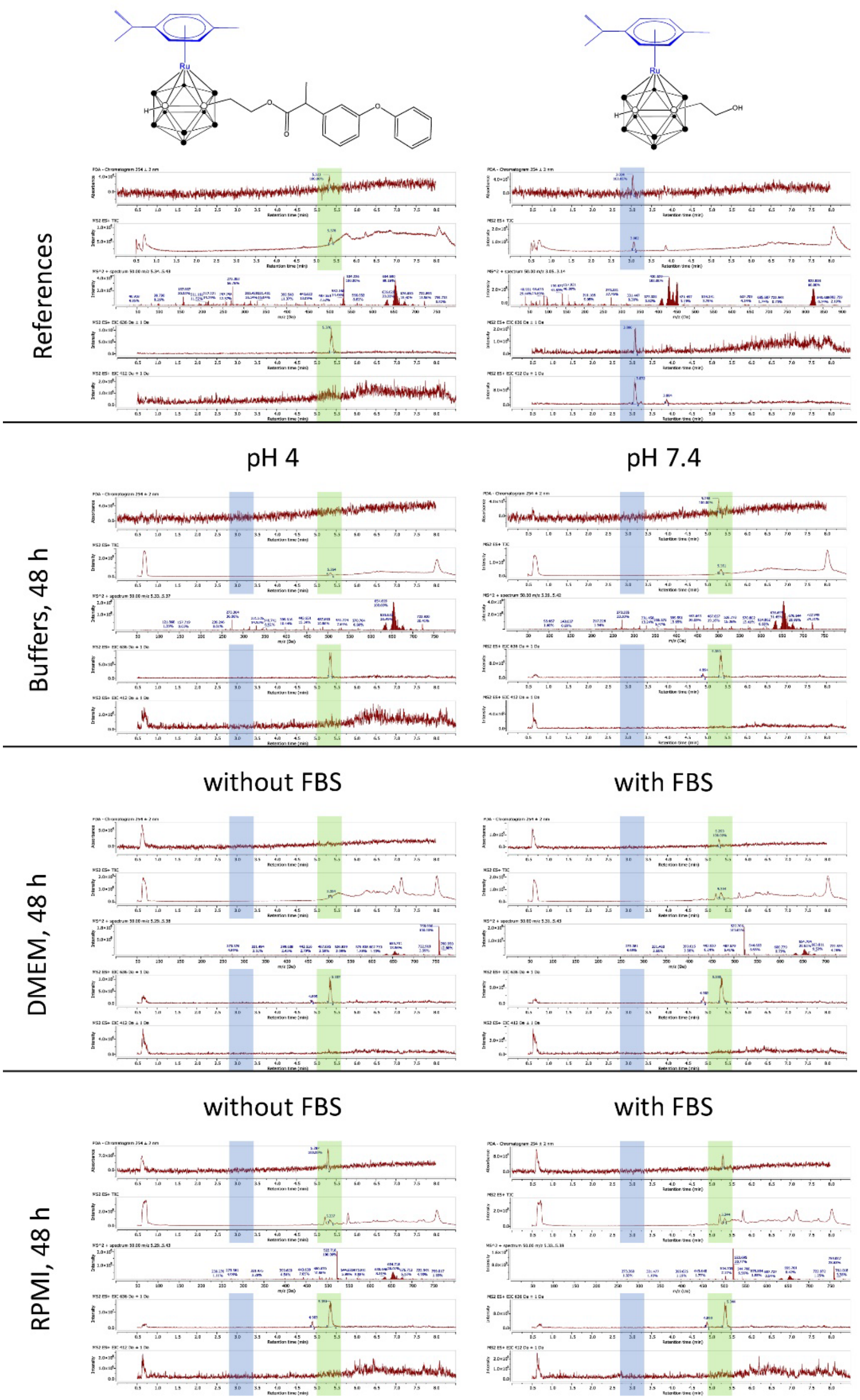

Figure S36: Stability studies of compound **5b** in various media over 48 h.

Each panel is the result of the injection of one sample and is made up of the following chromatograms and spectra (top to bottom): 1) PDA trace at 254 ± 2 nm, 2) TIC chromatogram with integrated peak of **3b** (top right panel) or **5b** (all others), 3) mass spectrum extracted from indicated peak in TIC, 4) TIC filtered for mass peak of **5b** (643 ± 1 Da), 5) TIC filtered for mass peak of **3b** (381 ± 1 Da). The retention times of **5b** (green) and **3b** (blue) are highlighted in all chromatograms. Panels are in the following order: top row) references **5b** (left) and **3b** (right), second row) samples from pH 4 (left) and pH 7.4 (right), third row) samples from DMEM without (left) and with (right) addition of 10 % FBS, bottom row) samples from RPMI without (left) and with (right) addition of 10 % FBS.

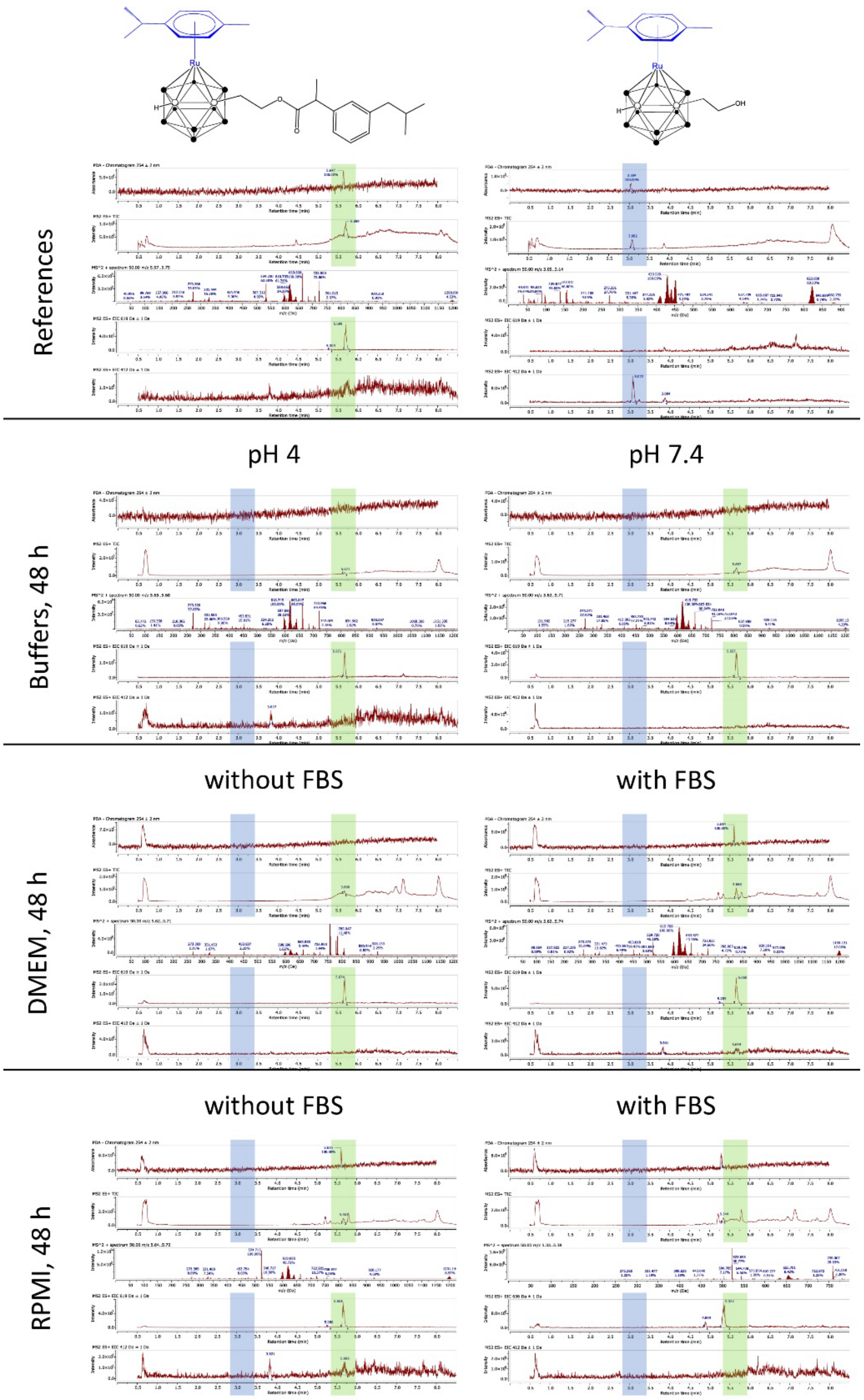

Figure S37: Stability studies of compound **6b** in various media over 48 h.

Each panel is the result of the injection of one sample and is made up of the following chromatograms and spectra (top to bottom): 1) PDA trace at 254 ± 2 nm, 2) TIC chromatogram with integrated peak of **3b** (top right panel) or **6b** (all others), 3) mass spectrum extracted from indicated peak in TIC, 4) TIC filtered for mass peak of **6b** (643 ± 1 Da), 5) TIC filtered for mass peak of **3b** (381 ± 1 Da). The retention times of **6b** (green) and **3b** (blue) are highlighted in all chromatograms. Panels are in the following order: top row) references **6b** (left) and **3b** (right), second row) samples from pH 4 (left) and pH 7.4 (right), third row) samples from DMEM without (left) and with (right) addition of 10 % FBS, bottom row) samples from RPMI without (left) and with (right) addition of 10 % FBS.
